# Cellular dynamics in tumour microenvironment along with lung cancer progression underscore spatial and evolutionary heterogeneity of neutrophil

**DOI:** 10.1002/ctm2.1340

**Published:** 2023-07-25

**Authors:** Haoxin Peng, Xiangrong Wu, Shaopeng Liu, Miao He, Chenshuo Tang, Yaokai Wen, Chao Xie, Ran Zhong, Caichen Li, Shan Xiong, Jun Liu, Hongbo Zheng, Jianxing He, Xu Lu, Wenhua Liang

**Affiliations:** ^1^ Department of Thoracic Oncology and Surgery, China State Key Laboratory of Respiratory Disease & National Clinical Research Center for Respiratory Disease the First Affiliated Hospital of Guangzhou Medical University Guangzhou China; ^2^ Deparment of Clinical Medicine Nanshan School Guangzhou Medical University Guangzhou China; ^3^ Department of Oncology Peking University Cancer Hospital & Institute Peking University Health Science Center, Peking University Beijing China; ^4^ Department of Oncology Shanghai Medical College, Fudan University Shanghai China; ^5^ Department of Computer Science Guangdong Polytechnic Normal University Guangzhou China; ^6^ Department of Artificial Intelligence Research Pazhou Lab Guangzhou China; ^7^ Deparment of Clinical Medicine Tongji University Shanghai China; ^8^ Department of Medical Oncology Shanghai Pulmonary Hospital & Thoracic Cancer Institute, Tongji University, School of Medicine Shanghai China; ^9^ Medical Department Genecast Biotechnology Co., Ltd Beijing China; ^10^ Department of Medical Oncology The First People's Hospital of Zhaoqing Zhaoqing China

**Keywords:** multiplex immunofluorescence, single‐cell RNA sequencing, tumour microenvironment, tumour‐associated neutrophil, tumour‐draining lymph node

## Abstract

**Background:**

The cellular dynamics in the tumour microenvironment (TME) along with non‐small cell lung cancer (NSCLC) progression remain unclear.

**Methods:**

Multiplex immunofluorescence test detecting 10 immune‐related markers on 553 primary tumour (PT) samples of NSCLC was conducted and spatial information in TME was assessed by the StarDist depth learning model. The single‐cell transcriptomic atlas of PT (*n* = 4) and paired tumour‐draining lymph nodes (TDLNs) (*n* = 5 for tumour‐invaded, *n* = 3 for tumour‐free) microenvironment was profiled. Various bioinformatics analyses based on Gene Expression Omnibus, TCGA and Array‐Express databases were also used to validate the discoveries.

**Results:**

Spatial distances of CD4+ T cells–CD38+ T cells, CD4+ T cells–neutrophils and CD38+ T cells–neutrophils prolonged and they were replaced by CD163+ macrophages in PT along with tumour progression. Neutrophils showed unique stage and location‐dependent prognostic effects. A high abundance of stromal neutrophils improved disease‐free survival in the early‐stage, whereas high intratumoural neutrophil infiltrates predicted poor prognosis in the mid‐to‐late‐stage. Significant molecular and functional reprogramming in PT and TDLN microenvironments was observed. Diverse interaction networks mediated by neutrophils were found between positive and negative TDLNs. Five phenotypically and functionally heterogeneous subtypes of tumour‐associated neutrophil (TAN) were further identified by pseudotime analysis, including TAN‐0 with antigen‐presenting function, TAN‐1 with strong expression of interferon (IFN)‐stimulated genes, the pro‐tumour TAN‐2 subcluster, the classical subset (TAN‐3) and the pro‐inflammatory subtype (TAN‐4). Loss of IFN‐stimulated signature and growing angiogenesis activity were discovered along the transitional trajectory. Eventually, a robust six neutrophil differentiation relevant genes‐based model was established, showing that low‐risk patients had longer overall survival time and may respond better to immunotherapy.

**Conclusions:**

The cellular composition, spatial location, molecular and functional changes in PT and TDLN microenvironments along with NSCLC progression were deciphered, highlighting the immunoregulatory roles and evolutionary heterogeneity of TANs.

## Graphical abstract

1. The cellular composition, spatial location, molecular and functional changes in primary tumour and paired tumour‐draining lymph node microenvironments along with non‐small cell lung cancer progression were comprehensively profiled.

2. Tumour‐associated neutrophils (TANs) showed unique spatial and temporal‐dependent prognostic effects.

3. The immunoregulatory roles and evolutionary heterogeneity of TANs during lymphatic metastasis were demonstrated.

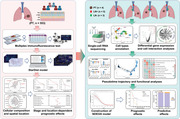

## 1 INTRODUCTION

Tumour metastasis is a leading reason of lung cancer (LC) death globally.^1^ Despite early‐stage non‐small cell lung cancer (NSCLC) patients with 5‐year overall survival (OS) rates of more than 80%, the occurrence rate of metastasis or postoperative relapse was up to 21.7%.^2^ Cancer cells metastasising to lymph nodes (LNs) is the early and crucial event of metastatic tumours, affecting treatment strategies and strongly predicting the poor prognosis.^3^

Tumour cells could acquire the competence of lymphatic metastasis in a passive or active manner. Some tumours, like breast cancer, were skewed to spreading by the lymphatic system and could passively enter it via the lymph vessels.^4^ Another mechanism is that tumour cells actively rebuild the microenvironment of the primary tumour (PT) and LNs, which include a range of molecular, cellular and structural alterations, to form the pre‐metastatic niche for their colonisation and growth. The lymphangiogenesis process is indispensable and vital in lymphatic invasion.^5^ Besides being the gateway for distant tumour dissemination, LNs also act as the immune foci that could orchestrate lymphocytes for coordinated adaptive immunity.^6^

The immunological roles of tumour‐draining lymph node (TDLN) have renewed scientists’ interest in the presence and advances of transcriptomic and proteomic techniques in late years.^7^, ^8^ For instance, Kim and colleagues have profiled the single‐cell transcriptomic atlas of LC in PT, LNs and distant metastatic lesions and revealed the depletion of T cells and the proliferation of macrophages along with tumour progression.^9^ Li et al.^10^ reported that cancer cells go through metabolic reprogramming in the TDLN, facilitating the release of lipids and other metabolites, which could shift cellular function and result in immune dysregulation. Recent studies also showed that TDLN serves as a critical element of immune checkpoint inhibitor (ICI) treatment efficiency,^11^ and targeting TDLN mediates a valid anti‐tumour immunity in PT of mouse model.^12^ And it underscores a potential treatment option for improving patients’ prognosis and calls for better characterisation of TDLN microenvironment for developing novel immunotherapy strategies.

As a vital element regulating cancer development in the tumour microenvironment (TME), the dual roles of neutrophils have been well demonstrated.^13^ Cell content,^14^ spatial location,^15^ activation status,^16^ and surrounding milieu^17^ in TME may account for such conflicting roles of neutrophils. For instance, Schürch and colleagues^18^ reported that infiltration of CD4+ PD‐1+ T cells in the neutrophil‐dominant cellular community predicted longer OS in colorectal cancer patients, implying the role of T cells‐neutrophils interactions in modulating immune reactivity. Concerning different tumour‐associated neutrophil (TAN) subsets, Salcher et al.^19^ recently reported four major subtypes in NSCLC by integration of single‐cell datasets, including TAN‐1 with activated neutrophil markers, TAN‐2 with AP feature, TAN‐3 with proinflammatory function and TAN‐4 with high expression levels of ribosomal genes. Nevertheless, most of the included samples in their study were collected from biopsies rather than the tissue block from lobe excision, thus may lose the information of sampling locations like TN and TS, which has been shown to affect the functionality of TANs due to different surrounding milieu. Moreover, the main analyses were conducted based on PT, while the immunomodulatory functions of TANs in TDLN, which is the pivot of immune surveillance and the initial site that determines anti‐tumour immunity, remain unclear.^20^ Moreover, systematic evidence concerning the changes in infiltrating and spatial patterns and the ontogeny of neutrophils during lymphatic metastasis procedure is still lacking.

In the present work, the cellular composition, spatial location, molecular and functional changes in PT and paired TDLN microenvironments along with NSCLC progression were deciphered by the integrated analyses of multiplex immunofluorescence (mIF) and single‐cell RNA sequencing (scRNA‐seq) data (Figure S1), highlighting the immunoregulatory roles and evolutionary heterogeneity of neutrophils.

## 2 METHODS

### 2.1 Study design and sample collection

Patients were eligible for inclusion if^1^: histologically confirmed stage IA∼IIIB NSCLC^2^, ^21^, ^22^; no prior systemic chemotherapy^3^; adequate resected tissues for mIF detection^4^; complete baseline and follow‐up data. We excluded patients who^1^: with in situ LC^2^; previously received preoperative neoadjuvant therapy. Disease‐free survival (DFS), the time from radical resection to relapse or death, was utilised as the endpoint. The last follow‐up date was set for 1 January 2018.

The current research was approved by the ethics committee of First Affiliated Hospital of Guangzhou Medical University and executed upon the Declaration of Helsinki.^23^ Informed consent was obtained per patient.

### 2.2 Workflow of mIF detection

The mIF detection was conducted at Genecast Biotechnology Co., Ltd. (Beijing, China). Ten immune‐related markers, including FOXP3, CD38, CD4, CD20 and CD66b in panel 1, and CD8, CD68, PD‐L1, CD163 and CD133 in panel 2 were detected.

A 4 μm thick section from formalin‐fixed and paraffin‐embedded NSCLC tissues was utilised for staining each panel. The sections underwent epitope extraction via boiling for 20 minutes (mins) in Tris–EDTA buffer at 97°C, subsequent to dewaxing and rehydrating. Then, endogenous peroxidase was blocked by incubating for 10 mins in antibody block/diluent while the protein was blocked in 0.05% Tween solution at room temperature for 30 mins. Subsequently, five antigens per panel were labelled by cyclic staining, including incubating primary (PAb) and secondary antibodies (SAb), amplifying and visualising tyramine signal (TSA) and removing the antibody–TSA compound in Tris–EDTA buffer by microwave process for 20 mins at 97°C.^24^ Each slice was counterstained with DAPI for 5 mins and mounted in Pro‐Long Diamond Antifade Mountant (Thermo Fisher) after cyclic dyeing. Information on the reagents used is available in Table S1.

Furthermore, PAb for CD4 and CD133 were incubated at 4°C for one night, while CD8, FOXP3, CD20, CD66b, CD38, CD163, PD‐L1 and CD68 were incubated for 1 h at 26°C. Details of PAb utilised are available in Table S2. For SAb, anti‐rabbit/mouse horseradish peroxidase antibodies were implemented and incubated at 37°C for 10 mins. Visualising TSA was realised via the Opal seven‐colour mIF Kit (NEL797B001KT; PerkinElmer), as we reported previously^25^, ^26^ (Table S3).

All sections were scanned through the PerkinElmer Vectra software. Both whole slide images (WSI) and high‐power fields (HPF) were analysed. A 4× objective lens (OL) to preview the full image of the slice was first used, followed by the 20× OL to capture more particulars. Images of individual fields were eventually spliced to get the full picture of the slice with high resolution (4028 × 3012 px). The HPF was set as 20× OL on the PerkinElmer Vectra system (Vectra 3.0.5; PerkinElmer), under which the tissue structure could be distinguished. A total of five HPFs were scanned per patient. Multispectral images were separated from spectral libraries by the inForm Advanced Image Analysis software. Information on the number of slides analysed per patient in the mIF test was provided in Table S4.

First, 25–30 mIF pictures in high resolution were randomly selected (Data S1). Second, a proficient pathologist (Dr. Bai Xuejuan) depicted the tumoural and stromal fields on these pictures to train the algorithm in the inForm software. Third, the inForm software was made competent for the detection and segmentation of tissue sections into tumour nest (TN) and tumour stroma (TS) upon the morphologies automatically (Figure S2A). Two practiced pathologists (Dr. Wang Xin and Dr. Bai Xuejuan) determined each marker's appropriate positive threshold ‘Z’ separately, and disagreements were solved by consensus. Z, 2Z and 3Z was regarded as the threshold of low, median and high fluorescence strength (FS), respectively. The histochemistry score (*H*‐score) was analysed as^25^:

$$\begin{array}{ccc} H-\mathrm{score} & = & \left(\mathrm{cells}\;\mathrm{with}\;\mathrm{low}\;\mathrm{FS}\right)\%\times 1\\ & & +\;\left(\mathrm{cells}\;\mathrm{with}\;\mathrm{median}\;\mathrm{FS}\right)\%\times 2\\ & & +\;\left(\mathrm{cell}\;\mathrm{with}\;\mathrm{high}\;\mathrm{FS}\right)\%\times 3 \end{array}$$

Information on annotating cell types via these 10 markers is available in Table S5.^27^

### 2.3 Identifying, segmentising and localising cells via deep learning model

Our dataset enrolled over 2700 WSIs from 553 NSCLC individuals. The workflow of the deep learning algorithm to identify, segment and locate cells is depicted in Figure S2B.

First, we separated the R, G and B colour channels and extracted the coordinates of fields with fluorescence (CF) of each pathological picture. Second, DAPI‐dyed slides were analysed using the StarDist deep learning model, the detailed depiction of which was previously reported,^15^ to gain the coordinates of the cell nuclei (CCN). Third, CF and CCN were intersected to gain the detailed coordinates of each cell with fluorescence after removing non‐specific fluorescence signal. Ultimately, the Delaunay triangulation approach was employed to connect cell nuclei and obtain the spatial relationships between different cell populations in TME.^28^

### 2.4 Extracting characteristics to profile the spatial associations among cells

The connection length between two cells was defined as the spatial distance between them. Because 10 markers were detected by mIF in the current study, a combination of 30 spatial variables was generated in TME for each patient:

$$\left[C_{5}^{2}+5\;=\;\frac{5!}{2!\;\times \;\left(5-2\right)!}+5\right]\times 2\;=\;30$$

The values of spatial variables were averaged if over one pathological section per patient was available.

### 2.5 Assessing the performance of the StarDist model

The threshold of intersection over union (IoU) of the StarDist model was set at 0.6, as Schmidt et al.^29^ previously suggested, which efficiently segments cells of pathological sections. Ground truth (GT) refers to the manual labelling of cell nuclei, which is the ‘truth value’ compared with the ‘predicted’ cell nuclei by the StarDist depth learning model.^30^ And nucleus was labelled ‘matched’ when IoU for GT nucleus > 0.6; otherwise, it was ‘unmatched’. Meanwhile, the proportion between the discriminated and GT nucleus was regarded as the detection coverage, assessed by F1‐score and pixel accuracy (PA), calculating as:

$$\mathrm{PA}=\frac{\mathrm{correctly}\;\mathrm{segmented}\;\mathrm{number}\;\mathrm{of}\;\mathrm{pixels}}{\mathrm{total}\;\mathrm{number}\;\mathrm{of}\;\mathrm{pixels}}$$

We first manually marked the cells and localised the nuclei of twenty randomly selected mIF images. Then the StarDist model automatically segmented cells of the same batch of mIF images. Eventually, we analysed the false positive (FP), false negative (FN) and true positive (TP) values based on GT nuclei and the nuclei predicted by the StarDist model. The recall and precision of segmenting cell nuclei were also counted to analyse the F1‐score^31^:

$$F1\;=\;2\times \frac{\mathrm{precision}\times recall}{\mathrm{precision}+\mathrm{recall}}=2\times \left(\frac{\frac{\mathrm{TP}}{\mathrm{TP}+\mathrm{FP}}\times \frac{\mathrm{TP}}{\mathrm{TP}+\mathrm{FN}}}{\frac{\mathrm{TP}}{\mathrm{TP}+\mathrm{FP}}+\frac{\mathrm{TP}}{\mathrm{TP}+\mathrm{FN}}}\right)$$

### 2.6 Evaluation of cellular composition and spatial distribution in the TME

The cellular composition was estimated by the percentage (%/sight) of them on the mIF image, while the spatial relationships were evaluated by the physical length between two cell lineages. Considering proximity between two cells is requisite for direct and indirect signalling transduction between them, a shorter distance between two cell populations suggests closer interplay, whereas a longer distance indicates the opposite. Eventually, 30 spatial features and 66 cell content features were generated.

### 2.7 scRNA‐seq implementation

Four PT and eight TDLN samples, including five tumour‐invaded TDLNs and 3 tumour‐free TDLNs, were extracted from fresh surgical resections and cryopreserved in ice‐cold H1640 culture media (Gibco, Life Technologies). Before digesting the tissue with 0.25% trypsin, phosphate‐buffered saline (PBS) was applied to rinse the specimens which were cut into 1 mm^3^ cubic pieces. Specimens were added with dispase (0.6 U/ml) and 10 mL of digestion medium with collagenase IV (100 U/mL) after being terminated by culture media supplemented with 10% foetal bovine serum. With the cells being maintained on the ice, the entire process of collecting live cells was completed in less than 1 h, which involved filtering the digested samples through a 70 μm nylon mesh, centrifuging the filtered cells at 4°C and 120×*g* for 5 mins, re‐suspending the cell pellets with ice‐cold red blood cell lysis buffer, filtering the cells through a 40‐μm nylon mesh, collecting the cells with PBS and calculating the number of live cells with an automatic cell counter. Using the Chromium Single Cell 3′ Library, Gel Bead & Multiplex Kit and Chip Kit (10× Genomics), the single cell suspensions were converted to separate barcoded scRNA‐seq libraries according to the manufacturer's protocol. Eventually, with a paired‐end 150–base pair (PE150) reading strategy (CapitalBio Technology), the scRNA‐seq libraries were built up by the Single Cell 3′ Library Gel Bead Kit V3 and thereby sequenced on the NovaSeq 6000 platform (Illumina).

### 2.8 In‐depth sequencing data procession and cell cluster annotation

Gene expression matrices were generated using Cell Ranger (10× Genomics) based on the GRCh38 build of the human reference genome and further processed via R. The Seurat package was employed to analyse the output‐filtered gene expression matrices. We used the following quality control steps to classify low‐quality cells: (i) cells expressing >5000 or <200 genes, (ii) >10% unique molecular identifiers (UMI) or (iii) <500 UMIs derived from the mitochondrial genome. Subsequent analyses were conducted upon the standard workflow of Seurat.

After normalisation and auto‐scaling, the expression matrix of cells was first summarised by principal component analysis, followed by Uniform Manifold Approximation and Projection approach, thereby generating cell clusters sharing similar characteristics. For each cluster, a final list of ranked marker genes was obtained using the FindAllMarkers function, which ranked the gene expression of each pairwise comparison by log2 fold change.^32^ Subsequently, manual annotation was employed based on classical and widely accepted markers of the corresponding cell types.^33^, ^34^ The entropy‐based indicator, namely ROGUE, was utilised to evaluate the purity of the identified cell populations.^35^

### 2.9 Intercellular and intracellular communication analyses

To investigate the putative intercellular receptor–ligand interactome, the CellChat algorithm in R was employed. The Cell‐Cell Contact and Secreted Signaling databases within CellChatDB^36^ were selected for the interaction analyses. The computeCommunProb and netVisual_bubble functions were implemented to visualise and compare the predicted interactions mediated by ligand–receptor pairs from a certain cell group to another. Only interactions occurring in all clusters were considered. To determine the dominant function of certain cell types in the TME, ligand–receptor interaction networks were ranked based on the information flows using the rankNet function. The iTALK approach, which can capture the strongly upregulated or downregulated ligand–receptor gene pairs,^37^ was also utilised to validate the findings of intercellular networks analysed by the CellChat algorithm. The iTALK method categorised the ligand–receptor pairs into four types: immune checkpoints, growth factors, cytokines and others. The NicheNet analysis, which could predict the target genes of the intracellular ligand–receptor communications, was conducted to further profile the intercellular gene regulation effects and signal transductions.^38^

### 2.10 Gene set enrichment and variation analysis

Differential gene expression analysis (DGEA), gene set enrichment analysis (GSEA) and gene set variation analysis (GSVA) were performed to explore the functional and pathway differences among different cell lineages.^39^ Pairwise DGEA for each cell type was performed using the limma package based on the statistical threshold: log2 (fold change) > 1 and Bonferroni‐corrected *p*_adj_< 0.01.^40^ A comprehensive list of Kyoto Encyclopedia of Genes and Genomes and gene ontology (GO) gene sets were used for GSVA and GSEA. Pathways distinguished by a Bonferroni‐corrected *p*_adj_< 0.05 were considered significant according to the Euclidean or cosine distance metric.^41^

### 2.11 Revelation of cell developmental trajectory

The differentiation trajectory of cells was inferred via the Monocle^42^ with branch points according to gene expression profiles, which demonstrate that different clades correspond to cell types with distinct differentiation states. Trajectory analysis was also validated by the partition‐based graph abstraction (PAGA) approach via the SCANPY package.^43^ After the cell trajectories were constructed, GSEA was performed to assess the functional enrichment of cells in different states. By comparing expression profiles, differential analysis was performed along the pseudotime, and state‐specific genes were identified.

### 2.12 External datasets utilised for analyses

The scRNA‐seq data of NSCLC were obtained from the Gene Expression Omnibus (https://www.ncbi.nlm.nih.gov/geo/) and Array‐Express (https://www.ebi.ac.uk/arrayexpress/) database, including the GSE200563,^44^ GSE123904^41^ and E‐MTAB‐6149^45^ cohorts. The clinicopathologic characteristics, somatic mutation and bulk RNA‑seq data of lung adenocarcinoma (LUAD) in the TCGA–LUAD cohort were extracted from the UCSC Xena (https://xena.ucsc.edu/) database.

### 2.13 Evaluation of cell composition and abundance by bulk RNA‑seq data

CIBERSORT, an algorithm that assessed the abundance of 22 kinds of immune cell lineages, including T cell, B cell, NK cell, monocyte, macrophage, dendritic cell (DC), mast cell, eosinophil, neutrophil and their subtypes, was adopted to estimate the immune infiltration patterns by bulk RNA‐seq data.^46^

### 2.14 Predicting immunotherapy response by gene expression data

Tumour mutational burden (TMB), defined as the number of somatic mutations per megabase of genome interrogated, is a well‐established biomarker to predict immunotherapy responses across solid tumours, and a higher TMB predicts better ICI response of NSCLC generally.^47^, ^48^ The TMB was calculated for each patient in the TCGA–LUAD cohort via maftools in R based on their somatic mutation data.^49^

### 2.15 Statistical analysis

The Mann–Whitney *U* test and Wilcoxon *t*‐test were implemented for two‐group data comparison, while multiple comparisons were evaluated by the Kruskal–Wallis single‐factor analysis of variance. The survival curves were generated via the Kaplan–Meier method, and the log‐rank test was employed to compare significant differences. Prognostic factors were analysed by univariate and multivariate Cox regression analyses. The predictive performance of the risk model was assessed by the receiver operating characteristic curve and time‐dependent area under the curve (AUC). To confirm the best cutoff point, the X‐tile software, which is outcome‐based and can select the optimal division of the data by selecting the highest *χ*2 value, was employed.^50^ Plots and statistical analyses were completed in GraphPad Prism, R, SPSS and X‐tile. *p* < 0.05 was defined as significant across statistical approaches.

## 3 RESULTS

### 3.1 Clinicopathologic features of the included patients

Surgically resected PT samples from 553 NSCLC patients were collected from 2009 to 2011, with LUAD as the majority (69.1%) (Table 1). A total of 126 cases (32.9%) in the early‐stage (IA–IIA) and 114 cases (67.1%) in the mid‐to‐late‐stage (IIB–IIIB) relapsed throughout the follow‐up time, and the median DFS of which was 1780 days and 803 days, respectively. Additionally, patients in the mid‐to‐late‐stage were more likely to harbour vascular tumour emboli (VTE) (*p* < 0.001).

**TABLE 1:** Clinicopathologic features of patients enrolled in the present study.

Characteristics	IA–IIA (*n* = 383)	IIB–IIIB (*n* = 170)	^*^ *p* Value
Age			0.838
≤60	188 (49.1%)	96 (40.0%)	
>60	195 (50.9%)	144 (60.0%)	
Gender			0.092
Male	224 (58.5%)	101 (59.4%)	
Female	159 (41.5%)	69 (40.6%)	
Histology			0.218
Lung adenocarcinoma	269 (70.2%)	113 (66.5%)	
Squamous cell lung cancer	92 (24.0%)	39 (22.9%)	
Others	22 (5.8%)	18 (10.6%)	
Vascular tumour emboli			<0.001
Yes	214 (55.9%)	45 (26.5%)	
No	169 (44.1%)	125 (73.5%)	
Visceral pleural invasion			0.103
PL0	157 (41.0%)	49 (28.8%)	
PL1	194 (50.7%)	117 (68.8%)	
PL2	32 (8.3%)	4 (2.4%)	
Dissected lymph nodes			0.001
0–14	130 (33.9%)	37 (21.8%)	
≥15	253 (66.1%)	133 (78.2%)	

* *p* Values of Mann–Whitney *U* test.

### 3.2 Segmenting performance of the StarDist algorithm

The F1‐score at object‐level precision and PA at pixel‐level precision was 81.39 and 99.996%, respectively. Consequently, utilising the StarDist algorithm to identify, segment and locate the cell nuclei was accurate and effective.

### 3.3 Dynamic changes in cellular composition and spatial location along with cancer progression

**FIGURE 1:**
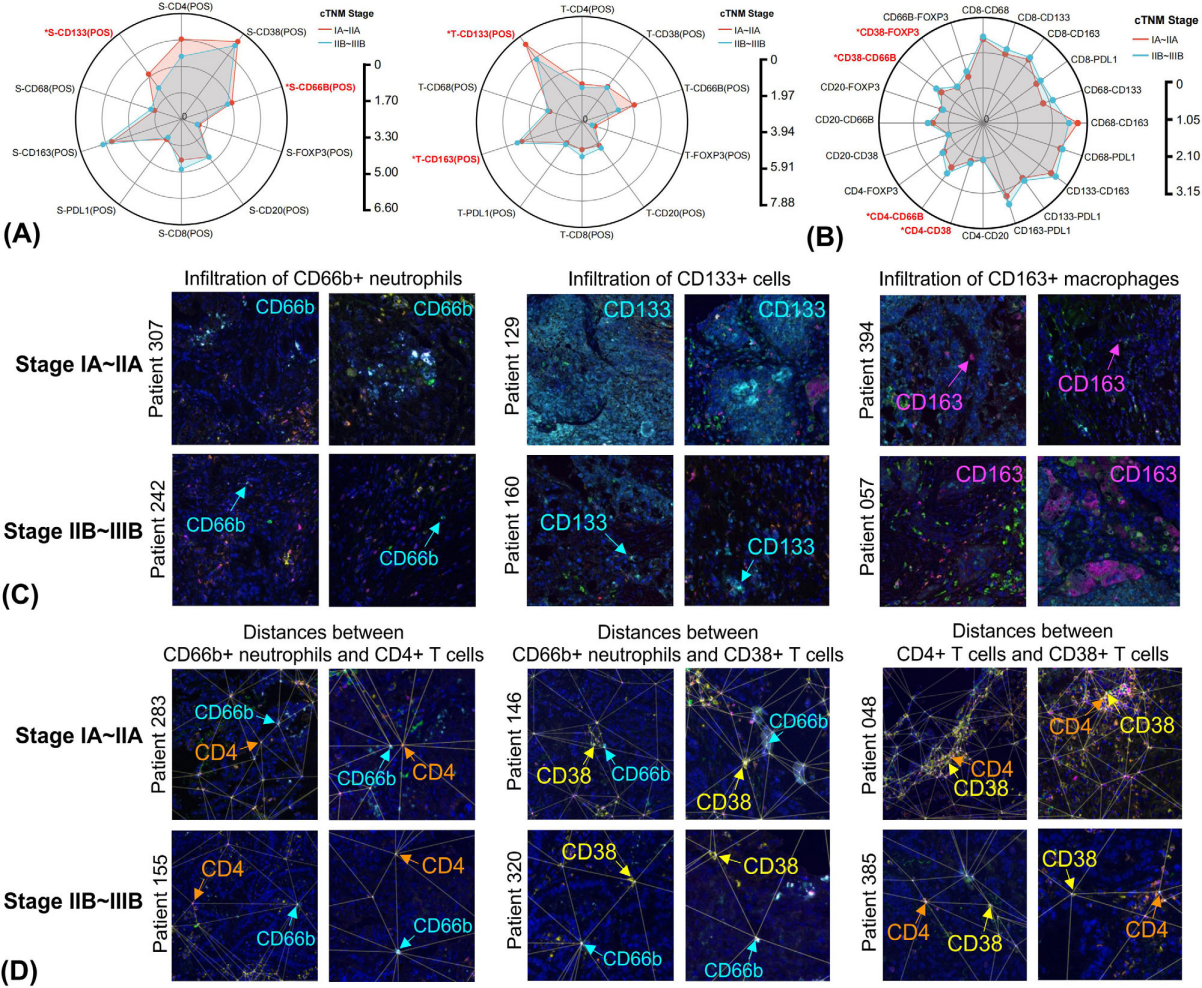
Dynamic changes of cellular composition and spatial location in the tumour microenvironment along with non‐small cell lung cancer (NSCLC) progression. Radar map comparing the cellular composition (A) and spatial distribution patterns (B) between early and middle‐to‐late‐stage NSCLC by Wilcoxon *t*‐test, highlighting significant findings. Representative multiplex immunofluorescence graphs depicting the changes in infiltration patterns (C) of CD66b+ neutrophils, CD133+ cells and CD163+ macrophages and spatial distances (D) of CD4+ T cells–CD66b+ neutrophils, CD38+ T cells–CD66b+ neutrophils and CD4+ T cells–CD38+ T cells between early and middle‐to‐late‐stage NSCLC within two fields from one tissue section. *p < 0.05.

From IA–IIA to IIB–IIIB stage, infiltration of neutrophils and CD133+ cancer stem cells (CSCs) was significantly reduced in TS (Figures 1A and C). Analogously, the content of CD133+ CSCs was also remarkably reduced in TN. Conversely, the abundance of CD163+ macrophages significantly increased in TN in the mid‐to‐late‐stage. Despite without statistical significance, both stromal and intratumoural CD4+ T cells, CD8+ T cells and CD20+ B cells showed a downward trend in infiltration with tumour progression, implying impaired anti‐tumour immune responses (Figures S3A–B).

Longer spatial distances of neutrophils‐CD4+ T cells, neutrophils‐CD38+ T cells, CD4+ T cells‐CD38+ T cells and CD38+ T cells‐regulatory T cells (Tregs) were observed in the mid‐to‐late‐stage than the early‐stage (Figures 1B and D). Distances of CD4+ T cells–CD20+ B cells and CD8+ T cells–CD8+ T cells also demonstrated an insignificant upward tendency (Figure S3C). Taken together, these results indicated that interactions among immunologic effector cells were attenuated, and they were gradually replaced by tumour‐associated macrophages (TAMs) with tumour progression.

### 3.4 Prognostic effects of infiltrating cellular contents

Multivariate Cox analysis adjusting for sex, age, T stage, visceral pleural invasion status and VTE status was conducted to evaluate the prognostic effects of different cell clusters. Higher infiltrating levels of CD8+ T cells in the early‐stage, irrespective of location, were associated with better prognosis (intrastromal: HR 0.890, 95%CI 0.812–0.976, *p* = 0.013; intrastromal: HR 0.808, 95%CI 0.703–0.928, *p* = 0.003), whereas such effects were insignificant in the mid‐to‐late‐stage (Figure S4A). Moreover, higher intratumoural Tregs infiltrates predicted unfavourable prognosis in the mid‐to‐late‐stage (HR 1.039, 95%CI 1.006–1.074, *p* = 0.022).

Subgroup analyses were also conducted based on different histological subtypes of NSCLC, including lung squamous cell carcinoma (LUSC) (Figure S4B) and LUAD (Figure S4C). Apart from showing similar findings in the overall group, higher intrastromal CD20+ B cell infiltrates in the early‐stage (HR 0.956, 95%CI 0.918–0.996, *p* = 0.032) predicted favourable DFS, whereas higher intratumoural CD68+ macrophages (HR 1.022, 95%CI 1.010–1.035, *p* < 0.001) infiltrates predicted the opposite in the LUAD subgroup. Interestingly, higher infiltrating levels of intratumoural M2 macrophages in the mid‐to‐late‐stage were associated with longer DFS in the LUSC group (HR 0.902, 95%CI 0.817–0.996, *p* = 0.042).

A higher ratio of intrastromal Tregs/CD4+ T cells predicted better prognosis both in the overall group (HR 0.171, 95%CI 0.044–0.660, *p* = 0.010) and the early‐stage (HR 0.039, 95%CI 0.003–0.449, *p* = 0.009) subgroup (Figure S4E). Subgroup analyses based on different histological types showed similar trends whereas without statistical significance (Figure S4D).

### 3.5 Neutrophils showed stage and location‐dependent prognostic effects

**FIGURE 2:**
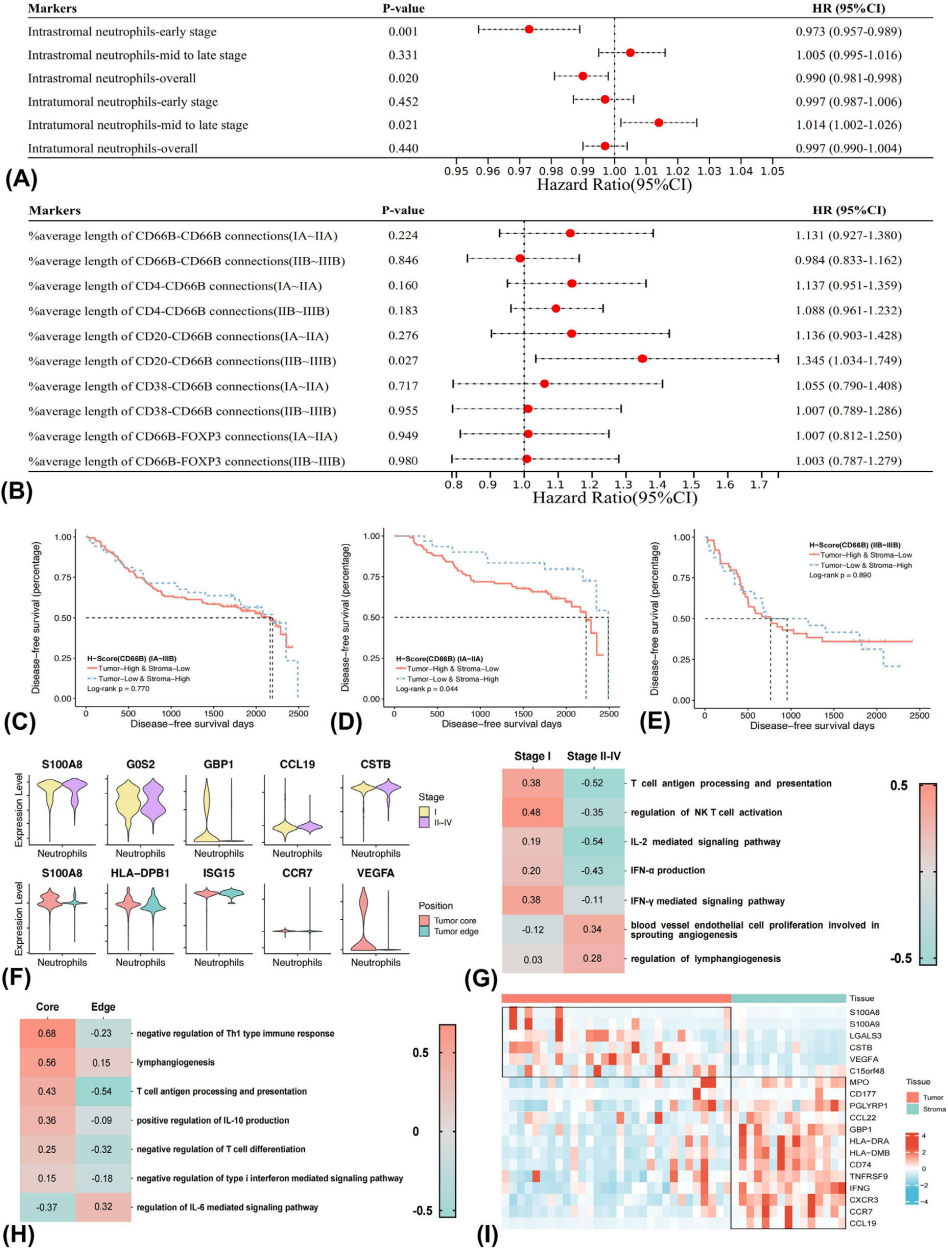
Neutrophils showed unique stage and location‐dependent prognostic effects in the tumour microenvironment. Forest plots showing the prognostic effects of infiltrating levels of neutrophils (A) and spatial relationships between neutrophils and other cell types (B), as evaluated by multivariate Cox regression analyses. Kaplan–Meier curves and log‐rank test demonstrating the disease‐free survival differences between tumour‐low and stroma‐high and tumour‐high and stroma‐low neutrophil infiltrates in the overall group (C), IA–IIA (D) and IIB–IIIB (E) subgroups. Different expressing levels of representative genes in neutrophils between different cTNM stages (F) and spatial location (G) based on re‐analyses of GSE123904, GSE200563 and E‐MTAB‐6149 datasets. Gene set variation analyses estimating the pathway activity of neutrophils in different cTNM stages (stage II–IV vs. stage I) (H) and spatial location (tumour core vs. tumour edge) (I).

We then sought to evaluate the prognostic effects of cells whose composition or spatial location notably changed along with cancer progression. Meta‐analysis with the fixed‐effects model was employed to pool the prognostic effects of neutrophils with different FS. We found that higher intrastromal neutrophil infiltrates (HR 0.990, 95%CI 0.981−0.998, *p* = 0.020), particularly in early‐stage (HR 0.973, 95%CI 0.957−0.989, *p* = 0.001), predicted significantly better prognosis (Figures 2A and S4F). In contrast, higher intratumoural neutrophil infiltrates in the mid‐to‐late stage (HR 1.014, 95%CI 1.002−1.026, *p* = 0.021) correlated with unfavourable prognosis. Neither intratumoural neutrophil infiltrates in the early‐stage nor intrastromal neutrophil infiltrates in the mid‐to‐late stage significantly correlated with prognosis. Moreover, a significantly better prognosis of patients with tumour‐low and stroma‐high than tumour‐high and stroma‐low neutrophil infiltrates in the early‐stage was observed (Log‐rank *p* = 0.044) (Figure 2D). A trend of better prognosis in patients with tumour‐low and stroma‐high neutrophil infiltrates was also found in the overall (Figure 2C) and the mid‐to‐late‐stage groups (Figure 2E), whereas without statistical significance. In subgroup analyses, high neutrophil infiltrates in the mid‐to‐late‐stage predicted poor prognosis both in LUSC (Figure S4G) and LUAD (Figure S4H). The prognostic effects of infiltrating macrophages (Figure S3D) and CD133+ CSCs (Figure S3E) did not significantly correlate with cellular location or cTNM stages.

Longer distances between neutrophils and CD20+ B cells predicted unfavourable DFS in the mid‐to‐late‐stage both in the overall (Figure 2B) and the LUAD (Figure S4J) but not LUSC (Figure S4I) subgroup. The prognostic significance of spatial distances of CD38+ T cells‐Tregs and CD4+ T cells‐CD38+ T cells did not display evident relationships with disease stages (Figure S3F). Overall, neutrophils showed unique stage and location‐dependent prognostic effects.

We then were interested in investigating the possible mechanisms underneath the spatial and temporal‐dependent prognostic effects of neutrophils based on publicly available scRNA‐seq datasets of GSE123904, GSE200563 and E‐MTAB‐6149 (Figures S5A and B and Tables S6 and 7). We found that expression of interferon (IFN)‐stimulated gene (e.g., GBP1) of neutrophils was significantly down‐regulated in the mid‐to‐late‐stage (stages II–IV) than early‐stage (stage I) (Figure 2F). GSVA further showed that differentially expressed genes (DEGs) of neutrophils between early and late stages were enriched into antigen presentation (AP) and IFN‐stimulated pathways in the early stage (Figure 2G). On the contrary, the DEGs were associated with angiogenesis and lymphangiogenesis processes in the late stages. Moreover, the expression of VEGFA was significantly higher in the TN, whereas the IFN‐stimulated gene (e.g., ISG15) was higher in the tumour edge. GSVA further indicated that DEGs of neutrophils between the tumour core and tumour edge correlated with negative regulation of Th1 type immune response and lymphangiogenesis pathways in the tumour core (Figure 2H). In contrast, the IL‐6 mediated signalling pathways were enriched in the tumour edge.

Spatial transcriptomic analyses based on the GSE200563 dataset indicated that classical marker genes of neutrophils expressed both in TN (e.g., S100A8 and S100A9) and TS (e.g., CD177 and PGLYRP1). However, the expression of genes participating in AP (e.g., HLA‐DRA/DMB and CD74) and anti‐tumour immunity (e.g., GBP1, IFNG and TNFRSF9) were significantly higher in the TS, while genes involved in angiogenesis (e.g., VEGFA) was significantly higher in TN (Figure 2I).

Collectively, findings from large‐scale mIF study and functional analyses based on scRNA‐seq datasets highlighted the spatial and temporal modulation of neutrophils along with cancer progression, and the distinct prognostic effects of neutrophils might drive by IFN production and angiogenesis pathways.

### 3.6 Microenvironment landscape in PT and paired TDLNs

**FIGURE 3:**
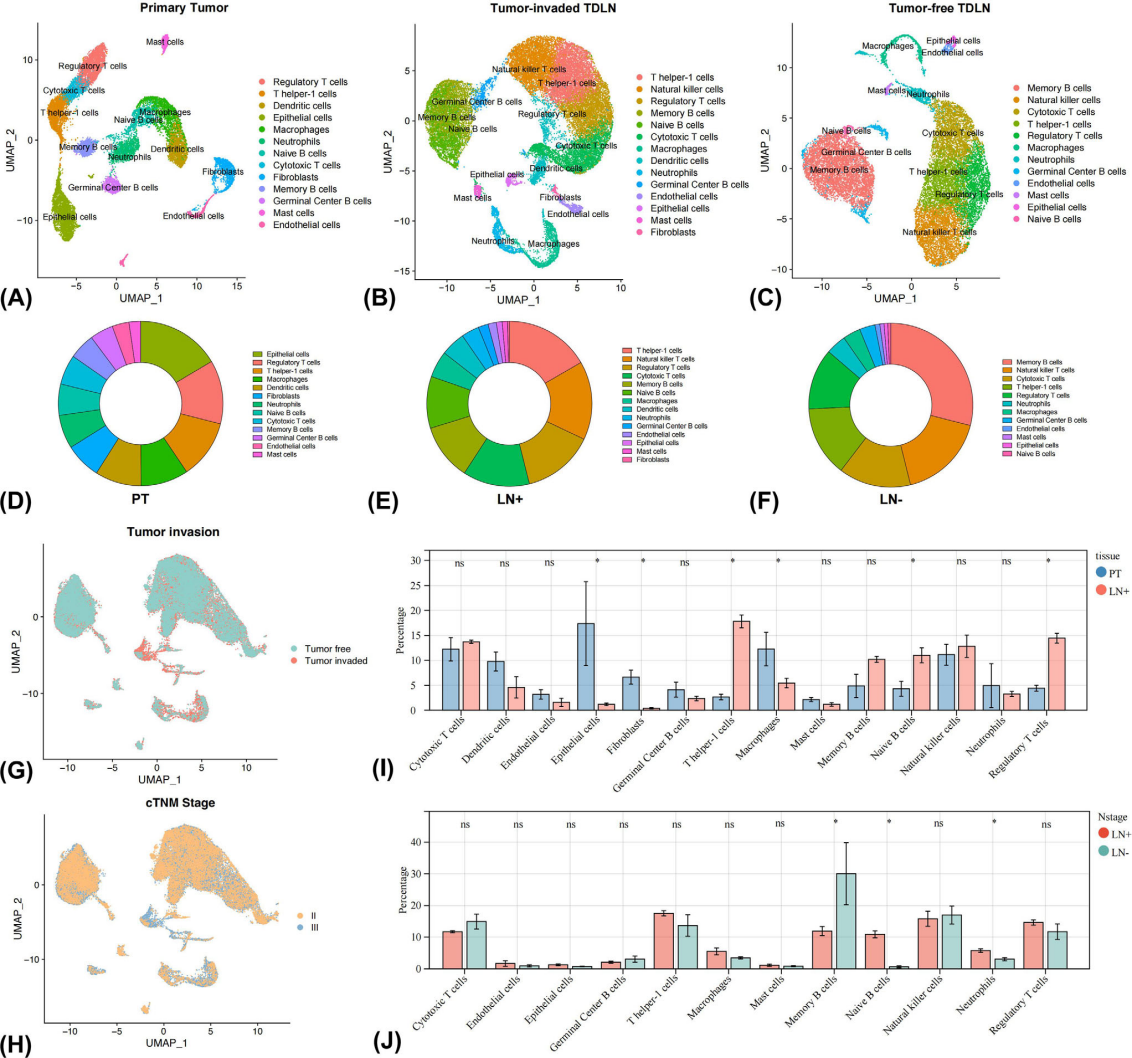
Microenvironment landscape in the primary tumour (PT) and tumour‐draining lymph node (TDLN) of non‐small cell lung cancer. UMAP plots demonstrated the identified cell lineages and cellular composition in PT (A and D) and paired positive (B and E) and negative (C and F) TDLN microenvironment. Cells in TDLNs were coloured upon the tumour invasion status (G) and cTNM stage (H). Comparison of cell contents in PT and paired TDLN microenvironment by Wilcoxon *t*‐test (I and J). *p < 0.05; ns, non‐significant.

Cancer cells metastasising to LNs is the common and early manifestation along with tumour progression. To profile the molecular and functional changes of cells and their roles in modulating cancer development, scRNA‐seq on PT and paired TDLNs was conducted (Figures 3A–C). After quality filtering, approximately 33.8 million unique transcripts from 16 459 cells in PT, 31.5 million unique transcripts from 19 975 cells in tumour‐free TDLN, and 48.0 million unique transcripts from 28 352 cells in tumour‐invaded TDLN were obtained, respectively. Cells could be classified into fourteen major lineages upon their canonical markers, including cytotoxic T cells (CTL), T helper‐1 (Th1) cells, Tregs, memory B cells, germinal centre (GC) B cells, naive B cells, natural killer (NK) cells, DC, macrophages, neutrophils, epithelial cells (EC), endothelial cells, fibroblasts and mast cells (Figure S5C–G and Table S8). The ROGUE values of all identified cell clusters in the PT, positive and negative TDLNs were greater than 0.6, and particularly neutrophils were greater than 0.7, suggesting relatively high purity (Figures S6A–C).

The most abundant cell types were EC, Th1 cells and memory B cells in PT, tumour‐invaded TDLN and tumour‐free TDLN, respectively (Figures 3A–F). Compared with tumour‐invaded TDLN, infiltration of EC, fibroblasts and macrophages were higher in PT, whereas Th1 cells were reduced (Figures 3G–I). Neutrophils and naïve B cells were more abundant, while memory B cells were reduced in tumour‐invaded TDLN than in tumour‐free TDLN (Figures 3H–J). We then explored the activity differences of these cell types between PT and TDLN microenvironments.

### 3.7 Molecular and functional reprogramming in TME with tumour progression

DGEA indicated that 16 genes were up‐regulated while three were down‐regulated of Th1 cells in tumour‐invaded TDLN compared with PT (Figure S7A). Higher expression levels of IFN‐γ, GZMA and CXCL13 of Th1 cells in PT than positive and negative TDLN were observed, suggesting an active anti‐tumour immune response in PT (Figure S7B).^51^ In contrast, STAT1, a crucial factor for Th1 maturation and differentiation,^52^ was more highly expressed in negative TDLN than in positive TDLN and PT. GSVA of significant genes showed higher pathway activity in negatively regulating immune activation (e.g., down‐regulation of interleukin (IL)−2 and type‐1 IFN signalling pathway) in tumour‐invaded TDLN (Figure S7C). GO results further unveiled the DEGs between positive TDLN and PT were enriched into negatively regulating T cell activation (Figure S7D).

Tregs expressed higher levels of inhibitory markers, such as HAVCR2/TIM3 and TIGIT in PT and tumour‐invaded TDLN (Figures S7E and F). GSVA results showed that down‐regulated genes of Tregs in tumour‐invaded TDLN versus PT were enriched in negatively regulating IL‐18 production, which is an important factor augmenting anti‐cancer immunity (Figure S7G).^53^ Moreover, significant genes in PT and tumour‐invaded TDLN correlated with transforming growth factor‐β1 (TGF‐β) production (Figure S7H). Collectively, suppressed Th1 activity and enhanced immunosuppressive functions of Treg were discovered in tumour‐invaded TDLN.

Activated GC B cell genes like CD83 and CD40 were highly expressed in tumour‐free TDLN, whereas the expression level of immature B cell markers like CD27 was higher in PT (Figures S7I and J). LAPTM5, a negative regulator of B cell maturation, was up‐regulated in tumour‐invaded TDLN than PT. Further, DEGs of B cells in tumour‐invaded TDLN and PT were associated with negatively modulating B cell differentiation and activation (Figure S7K). GO enrichment findings also implied that altered genes were enriched in regulating B cell activation process (Figure S7L). Briefly, the formation of GC B cells was attenuated in tumour‐invaded TDLN and PT.

DC intimately communicated with other immune effectors in TME, and a remarkably different transcriptomic spectrum was found between PT and tumour‐invaded TDLN (Figure S8A), underscoring functional divergence. Expression of AP genes like HLA‐DPA1 and HLA‐DPB1 was higher in PT. In contrast, LAMP3, which showed regulatory function on lymphocytes, was higher in tumour‐invaded TDLN (Figure S8B).^54^ A significant down‐regulating T and B lymphocytes proliferation pathway and up‐regulating lymphangiogenesis pathway of DC were elucidated in tumour‐invaded TDLN than PT (Figure S8C). GO enrichment analysis further suggested that the AP term was enriched (Figure S8D). Collectively, the AP capability of DC was attenuated, whereas lymphangiogenesis was augmented in tumour‐invaded TDLN, contributing to immune incompetence and tumour metastasis.

Fibroblastic reticular cells are the major and vital component in LN, which facilitate transporting, priming and activating of immune cells.^55^ Enhanced pathway activity in remodelling blood vessel and lymphangiogenesis was observed in tumour‐invaded TDLN, indicating active remodelling of stromal structure (Figure S8E–G). Regarding EC, 40 up‐regulated and 26 down‐regulated genes were identified in tumour‐invaded TDLN than PT and were analysed via the GSVA analysis (Figure S8H). Epithelial to mesenchymal transition and positive regulating migration pathway activity was higher in tumour‐invaded TDLN, supported by GO enrichment analysis (Figures S8I and J). To sum up, the invasion and malignant transformation ability of EC were enhanced in tumour‐invaded TDLN.

Sixty‐two up‐regulated and thirty‐eight down‐regulated genes of mast cells in tumour‐invaded TDLN than PT were identified (Figure S8K), and significant genes were enriched in activating T cells, B cells and NK T cells pathways in tumour‐free TDLN (Figure S8L). GO enrichment analysis further showed that positively regulating cell adhesion term was enriched (Figure S8 M). Collectively, mast cells may regulate intercellular adhesion and stimulate immune response in tumour‐free TDLN.

**FIGURE 4:**
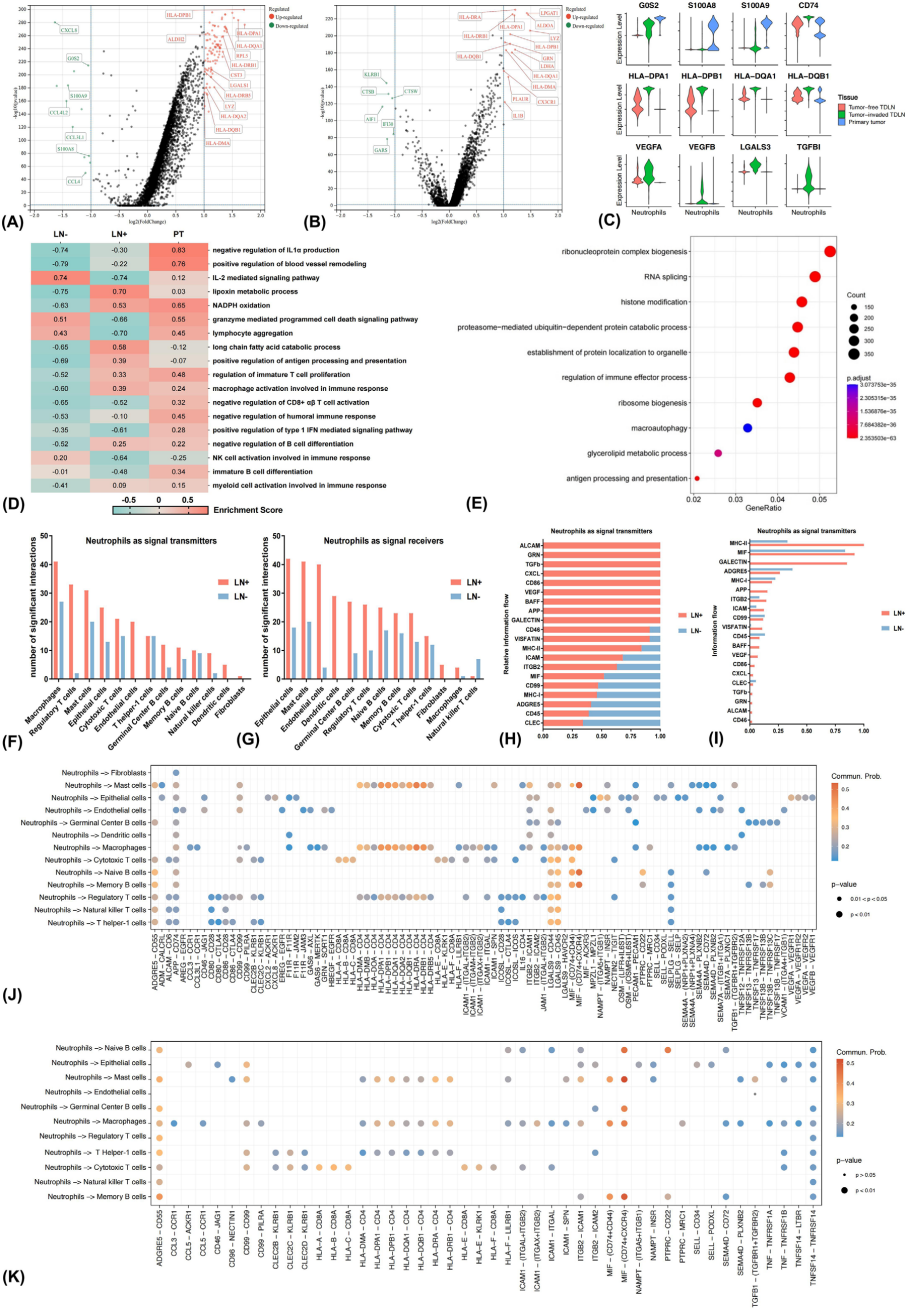
Differences in the transcriptomic atlas and interaction networks between primary tumour (PT) and tumour‐draining lymph node (TDLN) based on single‐cell RNA sequencing data of neutrophils. Volcano plots displaying differentially expressed genes (DEGs) of neutrophils in tumour‐invaded TDLN than PT (A) and tumour‐free TDLN (B). Violin plots demonstrating the differences in expression of representative function genes (C). Gene set variation analyses comparing pathway activity among PT, positive and negative TDLN by enrichment scores (D). Gene ontology analysis showing enriched biological process terms of DEGs in tumour‐invaded TDLN than PT (E). The numbers of remarkable receptor‐ligand communications between neutrophils as signal transmitters (F) or receivers (G) and other cell populations in positive and negative TDLN microenvironments. Comparing the relative (H) and overall (I) information flow of each signal pathway with neutrophils as signal transmitters between tumour‐invaded and tumour‐free TDLNs. Depiction of interaction probabilities with neutrophils as signal transmitters and other cell types as signal receivers mediated by ligand–receptor pairs in positive (J) and negative (K) TDLN.

A significant different transcriptomic spectrum of neutrophils was demonstrated between PT and TDLNs. Neutrophils in tumour‐invaded TDLN highly expressed AP (CD74 and HLA‐DPA1/DQA1), pro‐angiogenesis (VEGFA) and immunosuppressive (TGFBI)‐related genes (Figures 4A–C). Accordingly, a unique functional distinction that both high activity in immune‐stimulating pathways (e.g., positively regulating AP) and immunosuppressive pathways (e.g., negatively regulating type 1 IFN‐mediated pathway and NK cell activation) was observed (Figure 4D). GO enrichment findings further illustrated the glycerolipid metabolic process was enriched in positive TDLN (Figure 4E). Consequently, neutrophils displayed dual roles along with tumour progression in tumour‐invaded TDLN. Going further, we sought to explore the differences in communication networks mediated by neutrophils between negative and positive TDLN.

### 3.8 Neutrophils showed intimate interplay with Th1 cells and macrophages in tumour‐free and tumour‐invaded TDLN, respectively

With neutrophils as signal transmitters, macrophages and Tregs manifested significantly stronger interactions with neutrophils in tumour‐invaded TDLN. In contrast, intimate crosstalk between neutrophils and mast cells, Th1 cells and CTL were observed in tumour‐free TDLN (Figure 4F). With neutrophils as signal receivers, ECs and endothelial cells closely communicated with neutrophils in positive TDLN, while memory B cells and mast cells showed closer interactions with neutrophils in negative TDLN (Figure 4G).

We then evaluated the signal pathway activity between tumour‐free and tumour‐invaded TDLN. The relative information flow of CXCL, TGF‐β and VEGF was dramatically higher in positive TDLN (Figure 4H). Moreover, the leading signal pathways with neutrophils as signal transmitters were MHC‐II, MIF, GALECTIN in positive TDLN, and MIF, ADGRE5 and MHC‐II in negative TDLN, respectively (Figure 4I). We further assessed the crosstalk strength mediated by ligand–receptor pair from neutrophils to other cell types. In positive TDLN, neutrophils could recruit macrophages through CCL3/CCL3L1–CCR1 axes and may promote angiogenesis by VEGFA–VEGFR1/2 through interactions with endothelial cells (Figure 4J). Signals that inhibited the activation of T and B lymphocytes, such as LGALS9–CD44/45 and PTPRC–CD22 axes, were also augmented. In negative TDLN, neutrophils mainly communicated with Th1 cells (HLA−DRA/DQB−CD4) and CTL (HLA−A/B/C−CD8A) by AP process (Figure 4K).

By the iTALK method, intimate communications between neutrophils and endothelial cells through growth factors like VEGFA–ITGB1 and cytokines like CXCL12–CXCR4 pairs were observed in the tumour‐invaded TDLNs, involving in angiogenesis signal pathways (Figure S9A).^56^ Neutrophils may also hinder the activation of Th1 cells and CTL via immune checkpoints like CD86–CD28 and CD86–CTLA4 pairs in tumour‐invaded TDLN. On the contrary, closer interactions between neutrophils and Th1 cells and CTL through B2M–CD3D and B2M–KLRD1 pairs, involving in the AP pathway were observed in the tumour‐free TDLN (Figure S9B and C).^57^

The NicheNet method showed that, with neutrophils as signal transmitters in the tumour‐invaded TDLNs, CCL3/CCL4–CCR1 pairs were also dominant in mediating the interactions between neutrophils and macrophages, consistent with the findings of CellChat analysis (Figures S9D and F). Furthermore, the target genes of neutrophils‐macrophages/endothelial cells interactions were correlated with angiogenesis (e.g., VEGFA, VCAM1 and MMP14) and epithelial–mesenchymal transition (e.g., PDGFD, CXCL12 and IGF1) processes (Figures S9E and 9G).

Collectively, the CellChat, iTALK and NicheNet algorithms were utilised to investigate the intercellular interaction networks and intracellular gene regulation effects. Briefly, in negative TDLNs, neutrophils mainly communicated with Th1 cells and CTL through the AP pathway and may stimulate anti‐tumour immune reactivity consequently. On the contrary, intimate interactions between neutrophils and endothelial cells/macrophages correlated with angiogenesis and epithelial–mesenchymal transition processes, which may promote tumour progression.

### 3.9 TANs in tumour‐invaded TDLN consisted of a heterogeneous population

**FIGURE 5:**
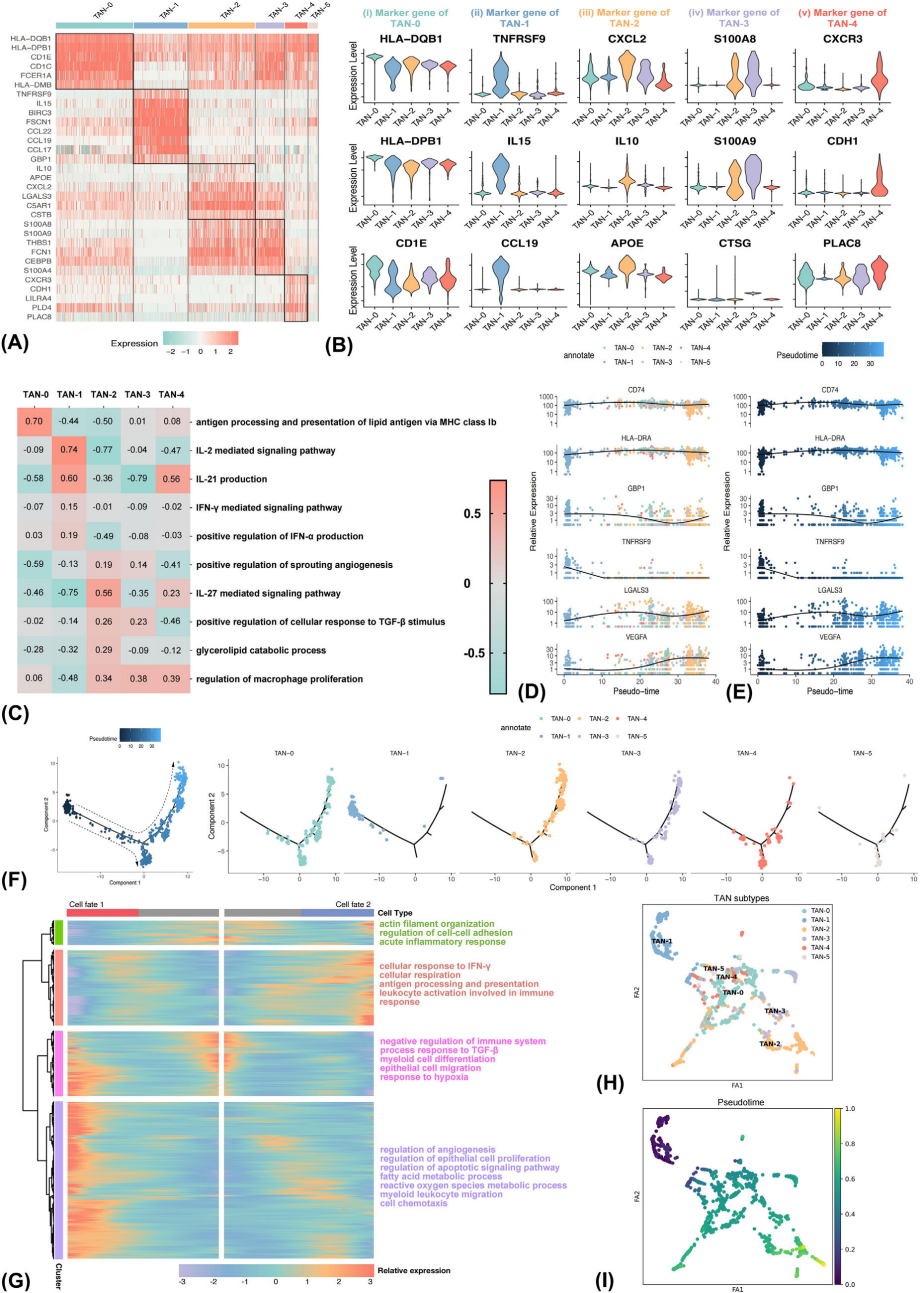
Tumour‐associated neutrophils (TAN) consist of phenotypically and functionally different subsets in the positive tumour‐draining lymph node microenvironment. Heatmap (A) and violin (B) plot showing the differential transcriptome spectrums of different TAN subsets. Gene set variation analyses comparing pathway activity among different TAN subtypes by enrichment scores (C). Two‐dimensional plots demonstrating the dynamic expressing levels of antigen presentation‐relevant genes (CD74 and HLA‐DRA), interferon‐γ stimulated gene (GBP1), costimulatory molecular‐related gene (TNFRSF9), immune regulation‐relevant gene (LGALS3) and angiogenesis related‐gene (VEGFA) along pseudotime trajectory (D and E). The trajectory of TANs along pseudotime in a two‐dimensional space was evaluated by the Monocle approach, with each point corresponding to a single cell (F). Heatmap displaying genes with dynamic expression levels along pseudotime, among which the differentially expressed genes could be hierarchically clustered into four groups with distinct enriched pathways (G). The SCANPY method validated the pseudotime analysis findings of TANs by the Monocle approach (H and I).

To dissect the heterogeneity of neutrophils, six major clusters were classified upon their distinctive transcriptomic profiles in tumour‐invaded TDLN (Figures 5A and B). The ROGUE values of these six subpopulations were all greater than 0.8, implying high purity (Figures S6D–F). TAN‐0 highly expressed AP‐related genes, like CD1E and HLA–DPB1. TAN‐1 exhibited strong expression levels of IL15 and CCL19, and costimulatory markers like TNFRSF9. TAN‐2 was characterised by higher expression levels of IL10 and APOE. TAN‐3 could be recognised as the classical subset for its strong expression of canonical markers of neutrophils, including S100A8/9 and CTSG. TAN‐4 comprised a pro‐inflammatory subtype, which showed high expression of inflammation‐associated markers like CXCR3 and PLAC8. No subtype‐specific characteristics of TAN‐5 were found.

GSVA was subsequently conducted to assess the variation of pathway activity between different TAN subtypes (Figure 5C). A significant up‐regulation of AP pathway activity of TAN‐0 was observed. Significant genes in TAN‐1 were enriched into IL‐2 and IFN‐γ‐mediated signalling pathways with immunostimulating competence. Enhanced pathway activity in the glycerolipid catabolic process and positively modulating angiogenesis and macrophage proliferation of TAN‐2 was found. TAN‐3 and TAN‐4 were similarly correlated with pro‐inflammatory processes like positively regulating macrophage proliferation.

### 3.10 Pseudotime trajectories unveiled the terminally differentiated state of the pro‐tumour TAN‐2 subset

To understand the dynamic transitional processes of TANs in TME, we further performed a pseudotime analysis to trace the transcriptional trajectory. The trajectory was designated to start with TAN‐1, through TAN‐3 and TAN‐4 as the intermediate states and eventually reached a terminal differentiation state characterised as TAN‐2 (Figure 5F). Features of TAN‐0 maintained along the pseudotime track. Similar findings that TAN‐0 was in the initiation phase, followed by TAN‐3 and TAN‐4 and terminally reached TAN‐2 state were validated by the PAGA method (Figures 5H and I). Expression of GBP1 and TNFRSF9 gradually decreased along the pseudotime trajectory, whereas the expression of VEGFA and LGALS3 showed the opposite trend (Figures 5D and E). Then GSVA was conducted to evaluate the enriched pathways of these genes in the TCGA–LUAD cohort. Higher expression levels of CD74 and HLA‐DRA were associated with higher AP pathway activity (Figures S10A and B). High expression of GBP1 was involved in IFN‐α and IFN‐γ production, and up‐regulation of TNFRSF9 was associated with lymphocyte activation, demonstrating immunostimulating effects (Figures S10C and D). In contrast, up‐regulation of VEGFA was enriched into angiogenesis‐related pathways, indicating pro‐tumour effects (Figures 10E and F).

We then identified 934 genes with notable expression alterations along the trajectory, and they could be classified into four patterns (Figure 5G). Expression of genes in cluster 1 gradually decreased along the trajectory, which was enriched in the innate immune reaction of neutrophils, like mediating acute inflammatory response and regulating intercellular adhesion, displaying the ontogeny from peripheral blood leucocytes to TANs.^58^ Cluster 2 contained genes highly activated in the early stage while down‐regulated in the late stage. They participated in anti‐tumour immune processes like AP, leukocyte activation and cellular response to IFN‐γ. In contrast, genes in clusters 3 and 4 were activated in the late stage and were associated with angiogenesis, myeloid leukocyte chemotaxis and EC proliferation. Moreover, the fatty acid metabolic process was also augmented. Taken together, neutrophils were composed of phenotypically and functionally heterogeneous populations in positive TDLN, and loss of IFN‐stimulated function and growing angiogenesis function with hyperactivated lipometabolism activity were the important events along the transition track.

**FIGURE 6:**
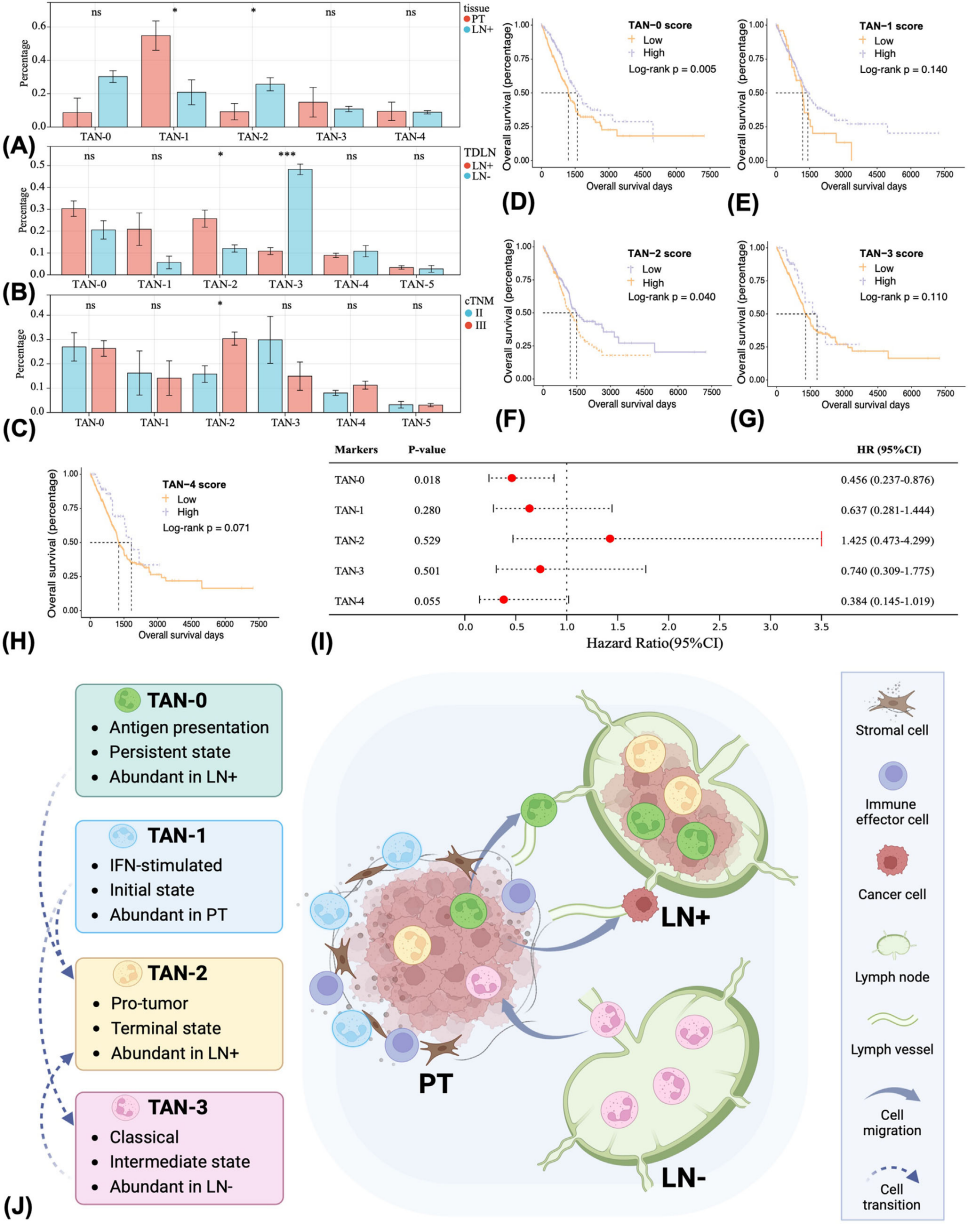
Infiltrating patterns and prognostic significance of different tumour‐associated neutrophil (TAN) subclusters in the non‐small cell lung cancer microenvironment. Comparing the contents of different TAN subtypes in different tissue types (A and B) and cTNM stages (C). Prognostic effects of different TAN signatures as evaluated by the log‐rank test (D–H) and multivariate Cox regression (I) analysis. The proposed model summarises the spatial and evolutionary heterogeneity of TANs (J). TAN‐1 with interferon‐stimulated function was abundant in the stroma of primary tumour, while TAN‐2 with pro‐tumour functions was abundant in the tumour nest. TAN‐3 with classical neutrophil features was the dominant TAN subtype in the negative tumour‐draining lymph node (TDLN), while TAN‐2 and TAN‐0 subtypes were abundant in the tumour‐invaded TDLN. The evolutionary trajectory was designated to start with TAN‐1, through TAN‐3 as the intermediate states, and eventually reached a terminal differentiation state characterised as TAN‐2. Features of TAN‐0 maintain along the trajectory. Comparison of two‐group data by Wilcoxon *t*‐test, *p < 0.05; ***p < 0.001; ns, non‐significant.

Finally, given the dramatic functional divergence among different neutrophil subpopulations, we were interested in exploring whether their amounts varied between tissue samples or cTNM stages. A total of 1097, 987 and 801 neutrophils in PT, positive and negative TDLNs, were identified, respectively. Results showed that the abundance of TAN‐1 significantly reduced in tumour‐invaded TDLN than PT, whereas amounts of TAN‐2 were strikingly higher (Figure 6A). Similarly, the abundance of TAN‐2 was significantly higher in positive than negative TDLN, whereas amounts of TAN‐3 were lower (Figure 6B). We further found that compared with the early‐stage, the abundance of TAN‐2 in positive TDLN increased in the late‐stage (Figure 6C).

### 3.11 Prognostic effects of different TAN signatures

Gene signatures of different TAN subsets were derived from the scRNA‐seq data and were used as the target gene sets (Table S9). Simple‐sample GSEA, an approach analysing the enrichment score (ES) of the pairing of each sample and target gene sets, was conducted to calculate the ES for each TAN subtype per patient in the TCGA–LUAD cohort.^59^ The cohort was divided into the high and low ES groups based on the optimal cutoff value via the X‐tile method. We found that patients with high TAN‐0 scores have significantly better OS than low ones (Log‐rank *p* = 0.005), whereas high TAN‐2 scores predicted the opposite outcome (Log‐rank *p* = 0.040) (Figures 6D–H). The prognostic effects of TAN‐0 signature remained significant in the multivariate Cox regression analysis (HR 0.456, 95%CI 0.237–0.876, *p* = 0.018) (Figure 6I), indicating the clinical relevance of the TAN signatures.

### 3.12 Establishment of a polygenic risk model based on neutrophil differentiation expression genes

To investigate whether the transcriptional changes of neutrophils would lead to a worse prognosis, we evaluated the prognostic effects of DEGs between PT, positive and negative TDLNs and built a polygenic risk model, namely the neutrophil differentiation expression gene score (NDEGS).

**FIGURE 7:**
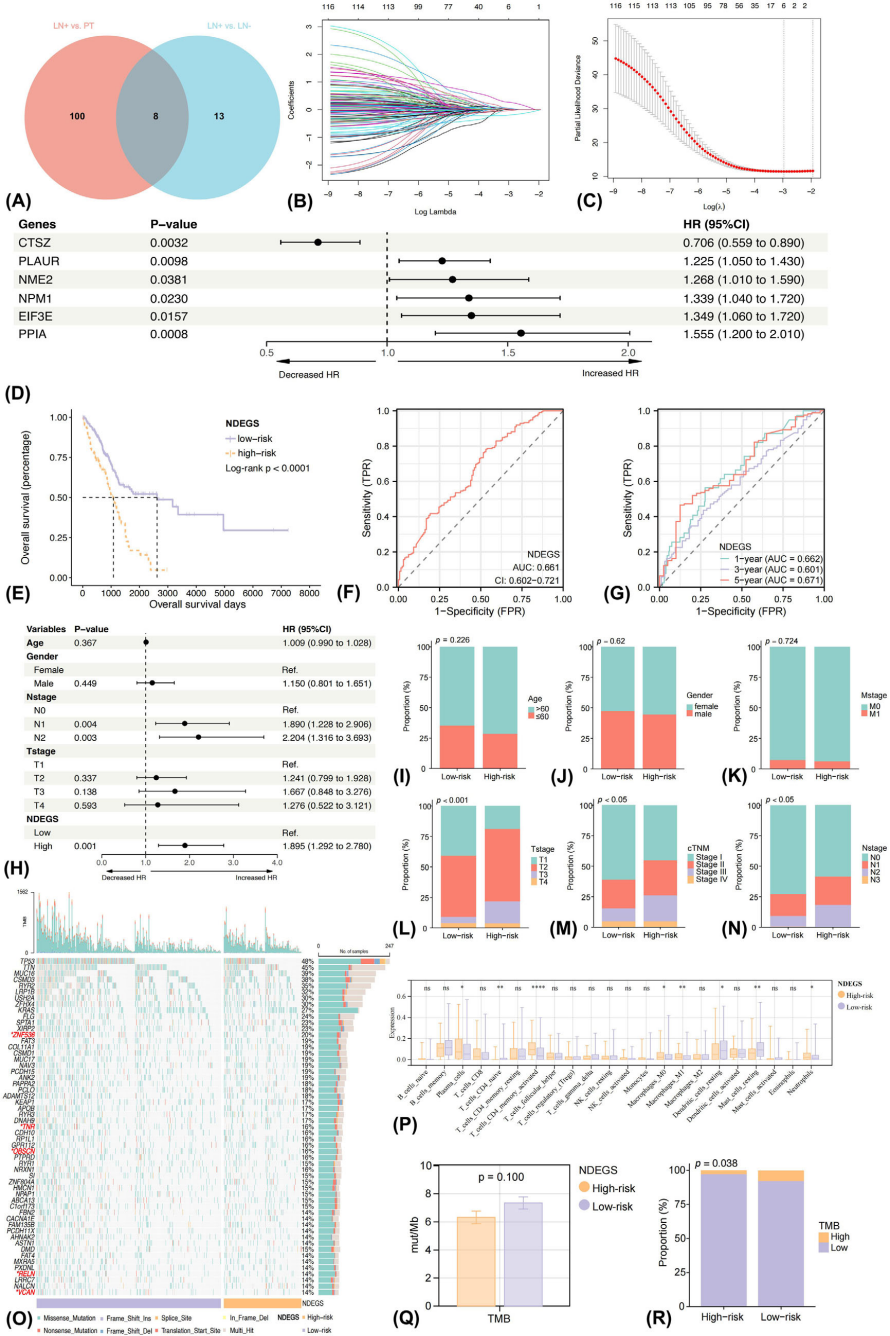
Construction of the neutrophil differentiation expressed gene score (NDEGS) model in the training cohort. Venn plot demonstrating the intersection and combination of neutrophil differentially expressed genes (NDEGs) among primary tumour, positive and negative tumour‐draining lymph node (A). Six robust NDEGs, including CTSZ, PLAUR, NME2, NPM1, EIF3E and PPIA, were selected by the LASSO Cox regression model (B and C), with prognostic effects in overall survival (OS) as evaluated by the univariate Cox regression analysis (D). Kaplan–Meier curve showing the overall survival (OS) rate differences between high and low‐NDEGS groups (E). Time‐dependent ROC curves and AUC values evaluate the prognostic performance of the NDEGS model at 1, 3 and 5 years (F and G). Forest plot implying the prognostic effects of the NDEGS model, as evaluated by the multivariate Cox regression analysis (H). Differences in clinicopathologic features (I–N), mutational (O) and immune infiltrating landscapes (P) between high and low‐NDEGS groups. Tumour mutational burden differences between low and high‐NDEGS groups (Q and R). *p* Values of the ANOVA and chi‐square tests between different groups. *p < 0.05; **p < 0.01; ****p < 0.0001; ns, non‐significant.

Group 1 included 100 genes differentially expressed in positive TDLN than PT, and the number of significant genes between positive and negative TDLN in group 2 was 13 (Figure 7A). Eight genes were intersected between group 1 and group 2, and the combination was 121. The TCGA–LUAD cohort was split into a training (*n* = 344) and a validating (*n* = 148) cohort at a ratio of 7:3. Then the expression data of candidate genes were extracted, of which five genes were absent in the TCGA–LUAD dataset, including RACK1, SNHG29, YBX3, ATP5MC2 and CXCL8, and hence were excluded. Eventually, 116 genes were used as the candidate genes and were recruited into the Least Absolute Shrinkage and Selection Operator (LASSO) Cox regression model to screen out the robust prognosticators in the training cohort (Figures 7B and C). A total of six genes, of which five were up‐regulated in positive TDLN than PT (CTSZ, NME2, NPM1, EIF3E and PPIA) and one was up‐regulated in positive than negative TDLN (PLAUR), were ultimately identified (Figure 7D). Both univariate and multivariate Cox analyses confirmed their prognostic effects (Figure S11).

Next, to better comprehend their functions, we investigated the corresponding pathway alterations between high and low expression of these six genes. A high expression level of CTSZ predicted better OS (Figure S10G) and was associated with normal immune responses of neutrophils like degranulation and respiratory burst (Figure S10M). Moreover, AP and lymphocyte aggregation processes were also enriched. Up‐regulation of PLAUR was associated with a dismal prognosis (Figure S10H) and was involved in lymphangiogenesis and negative regulation of Th1 immune responses (Figure S10N). Strongly expressed NME2 (Figure S10I) and NPM1 (Figure S10J) correlated with poor prognosis, and the pathways were enriched into the assembly of ribosome and proteasome, which were involved in the growth of cells (Figures S10O and P). High‐EIF3E (Figure S10K) and PPIA (Figure S10L) expression predicted worse OS and was involved in mitochondrial function and lipid metabolism pathways (Figure S10Q and R). Overexpression of PPIA was additionally associated with cellular copper ion homeostasis.

Then the risk score per patient was determined as: NDEGS = 0.706 × CTSZ_Exp_ + 1.225 × PLAUR_Exp_ + 1.268 × NME2_Exp_ + 1.339 × NPM1_Exp_ + 1.349 × EIF3E_Exp_ + 1.555 × PPIA_Exp_ (Table S10). Upon the optimal cutoff value, patients were subsequently divided into a high‐ and low‐NDEGS group. Low‐NDEGS ones displayed significantly longer OS than those of high‐NDEGS (Log‐rank *p* < 0.0001) (Figure 7E). Multivariate Cox regression analysis further illustrated that the NDEGS model was an independent prognosticator of OS (HR 1.895, 95%CI 1.292−2.780, *p* = 0.001) (Figure 7H), supported by the validating (Figure S12A–J) (HR 1.783, 95%CI 1.007−3.158, *p* = 0.047) and the entire (HR 1.811, 95%CI 1.326−2.475, *p* < 0.001) (Figures S12K–T) cohort.

The NDEGS model was also a relatively stable and strong predictor of NSCLC survival since the AUC was all greater than 0.65 among the training (AUC 0.661, 95%CI 0.602−0.721), validating (AUC 0.660, 95%CI 0.569−0.751) and entire (AUC 0.661, 95%CI 0.612−0.711) cohort (Figure 7F). Moreover, the AUC for predicting 1‐year, 3‐year and 5‐year OS in the training cohort was 0.662, 0.601 and 0.671, respectively (Figure 7G). Additionally, patients in the high‐NDEGS group tended to be in a more advanced T stage, N stage and cTNM stage (Figures 7I–N). Comparable findings were demonstrated in the validating and entire cohorts. Collectively, the NDEGS model was effective and stable, with the potential to predict survival.

### 3.13 Mutation and immune infiltration landscape between low and high NDEGS group

A total of 5 genes with significantly higher mutation rates, including ZNF536 (24.8 vs. 15.3%), TNR (19.9 vs. 15.4%), OBSCN (19.3 vs. 12.4%), RELN (17.4 vs. 10.9%) and VCAN (17.1 vs. 10.2%), were found in the low‐NDEGS group compared with high‐NDEGS group (Figure 7O). And it has been recently reported that mutation of OBSCN,^60^ RELN^61^ and VACN^62^ favour higher infiltrating levels of immune cells, which was also an indicator for better immunotherapy benefit than those of wild‐type. CIBERSORT approach further implied that the abundance of CD4+ T cells and memory B cells were dramatically higher, while neutrophils were significantly lower in the low‐NDEGS group (Figure 7P). Inversely, the abundance of monocytes and eosinophils significantly increased in the high‐NDEGS group, indicating the TME was dominated by myeloid‐derived suppressor cells (MDSCs) and possibly held an immunosuppressive milieu.

### 3.14 Association between the NDEGS and immunotherapy response

We further estimated the relationship between NDEGS and ICI efficiency via TMB, a widely adopted immunotherapy predictive marker. A trend of higher TMB in low‐NDEGS patients than high ones was observed (mean value: 7.35mut/Mb vs. 6.32 mut/Mb, *p* = 0.100) (Figure 7Q). Then 20mut/Mb was used as the cutoff value to define TMB‐H and TMB‐L groups as previously suggested.^63^ We found that the proportion of TMB‐H patients was significantly higher in the low‐NDEGS group (7.89 vs. 2.82%, *p* = 0.038) (Figure 7R). Therefore, with a higher TMB, patients with low‐NDEGS were more likely to benefit from immunotherapy.

## 4 DISCUSSION

In the present work, we comprehensively profiled the cellular and molecular changes in PT and TDLN microenvironments along with cancer progression, highlighting the spatial and evolutionary heterogeneity of neutrophils.

Reduced infiltration of neutrophils, CD4+ T cells and CSCs with tumour progression was found, accompanied by increased TAMs infiltration. Moreover, spatial distances of CD4+ T cells–CD38+ T cells, CD4+ T cells–neutrophils and CD38+ T cells–neutrophils prolonged at the late‐stage, implying attenuated interactions between them. The remodelling of immune infiltrating patterns could contribute to immune dysfunction and immune escape of tumour. Although former studies have reported changes in cell content along with cancer progression, their findings were concluded based on a small sample^64^ or indirect estimation by scRNA‐seq data,^9^ thus raising inevitable bias. To our knowledge, we depicted the most comprehensive cellular infiltration and spatial location changes in PT between early and mid‐to‐late‐stage NSCLC by a large‐scale mIF cohort.

Then the single‐cell transcriptomic atlas of PT and TDLN was depicted, and molecular and functional alterations of cells were compared, highlighting a series of crucial modulations along with lymphatic metastasis. The invasion and malignant transformation ability of ECs were enhanced in tumour‐invaded TDLN. However, the tumour AP capability of DCs was attenuated, while lymphangiogenesis was augmented, contributing to immune incompetence and tumour metastasis. Augmented negative immune regulatory functions of Tregs were also delivered in tumour‐invaded TDLN. Moreover, the activity of Th1 cells, which serve as the key player in initiating and amplifying adaptive immunity, was suppressed. The formation of GC B cells, which synergistically control tumour, was also attenuated in tumour‐invaded TDLN and PT.^65^ Collectively, positive TDLN displayed a highly immunosuppressive milieu, which may be more conducive to tumour cell survival than PT. The impaired anti‐tumour immunity also offered treatment options to reverse the aberrant factors into productive ones by ICI or targeting a specific cell lineage.

Intriguingly, neutrophils exhibited unique stage and location‐dependent prognostic effects among various cell populations. A higher abundance of intrastromal neutrophils was associated with a better prognosis in the early stages. In contrast, higher intratumoural neutrophil infiltrates predicted an unfavourable prognosis in metastatic stages. We further found that the different IFN production and angiogenesis‐related pathway activities in different disease stages and spatial regions could account for its dual roles, suggesting the transition from ‘antitumour’ TANs to ‘protumour’ TANs. A recent study by Hirschhorn et al.^66^ supported similar hypothesis that TANs exhibited direct tumouricidal effects in the acute inflammation setting whereas suppressing anti‐tumour immunity in the chronic inflammation situation. This may also partly explain why most retrospective studies found that high neutrophil infiltrates were an unfavourable prognosticator of cancer patients because chronic inflammation is one of the hallmarks of cancers.^67^ A recent study also revealed similar prognostic effects of neutrophils in a small cohort of head and neck cancer (HNC) patients.^68^ However, none of the studies have reported such phenomenon in LC. Likewise, despite researchers previously found that neutrophils could active T cells by presenting tumour antigens^69^ and releasing IFN‐γ^70^ in PT of early‐stage LC, none of them have investigated the immunoregulatory roles of neutrophils in TDLN, which centralises lymphocytes for synergistic anti‐tumour immunity.

Therefore, we subsequently parsed the functional spectrums of neutrophils in TDLN. Strikingly, neutrophils in tumour‐invaded TDLN elucidated seemingly conflicting immunomodulatory roles that both immune‐stimulating and immunosuppressive pathways were enriched. Furthermore, intimate interplays mediated by ligand–receptor pairs among neutrophils, Tregs and macrophages were observed in positive TDLN. Likewise, TANs and macrophages were reported to synergistically promote hepatocellular carcinoma development and drug resistance.^71^ In contrast, intimate crosstalk among neutrophils, Th1 and CTL, mainly enriching in the AP pathway, was found in negative TDLN. And it was supported by the latest evidence in HNC that neutrophils promote anti‐tumour immunity by sampling tumour antigens in PT and presenting them to T cells through migration to LNs.^68^

Going further, five phenotypically and functionally heterogeneous neutrophil subsets were identified in positive TDLN. And several distinct points are noteworthy. First, the expression of AP‐related genes reserved at a high level across the five neutrophil subtypes in TDLN, different from previous studies reporting that the AP competence of neutrophils blunted or even disappeared along with tumour progression in PT.^72^ Second, the expression of IFN‐stimulated genes like GBP1 and costimulatory markers like TNFRSF9 were significantly higher in TAN‐1 than in other subtypes while gradually decreasing along the pseudotime trajectory. In contrast, the expression of VEGFA was up‐regulated within the transition track and was highest in the terminal state, indicating the terminally differentiated role of the pro‐tumour TAN‐2 subpopulation. Third, hyperactivated lipometabolism activity was observed along the transition track. And overexpressed fatty acid transport protein‐2 has been reported to mediate the reprogramming of neutrophils into immunosuppressive directions in the murine tumour model, hinting at the potential therapeutic target.^73^ Fourth, the depletion of TAN‐1 with IFN‐stimulated function in PT and the emergence of pro‐tumour TAN‐2 in positive TDLN was observed, unveiling dynamic cellular composition changes during the lymphatic invasion. Moreover, the prognostic efficacy of the TAN‐0 and TAN‐2 signatures was demonstrated. Figure 6J summarises the above major findings concerning the spatial and evolutionary heterogeneity of TANs.

Zilionis et al.^74^ and Salcher et al.^19^ have previously decoded the diverse neutrophil subsets in PT of LC, and several common patterns could be drawn. The subset expressing classical neutrophil genes like S100A9 and S100A12 is like TAN‐3 as we proposed, while the subset with AP feature corresponds to TAN‐0, and the pro‐inflammatory subset was like TAN‐4 in our study. Except for similarities with prior work, our study showed that neutrophils in TDLN microenvironment additionally displayed higher diversity.

In other disease conditions, Xie and colleagues have previously dissected the heterogeneity of neutrophils during bacterial infection and identified eight subpopulations with different molecular signatures, among which a subtype with IFN‐stimulated function is found to develop from mature neutrophils in bone marrow directly.^75^ Interestingly, such a group of IFN‐stimulated TANs has also been identified in LC^74^ and pancreatic cancer,^72^ suggesting the conserved neutrophil subtypes in different disease conditions. Besides, Lecot et al.^76^ reported that a subgroup of peripheral neutrophils that bind to neutrophil–platelet aggregates hold a higher capacity for chemotaxis and trans‐endothelial migration, which predicts dismal prognosis in solid cancers.

Through in‐depth characterising of TAN subsets, our findings indicated that TANs were highly plastic and the conflicting roles of TANs might be attributed to their different subsets. Possible mechanisms underneath the transition between different TAN subtypes have been proposed. For instance, granulocyte‐macrophage colony‐stimulating factor (GM‐CSF) released by tumour cells could induce the formation of immunosuppressive neutrophils via activating GM‐CSF–PD‐L1 pathways in gastric cancer.^77^ In contrast, GM‐CSF is the requisite factor for generating neutrophils with AP ability in LC.^69^ Tumour cells could also hijack neutrophils and drive their pro‐tumour functions by secreting TGF‐β and chemokines like CXCL5.^17^, ^78^ Conversely, IFN‐β and IFN‐γ polarise neutrophils into an anti‐tumour status.^79^ As TAN‐0 and TAN‐1 are regarded as the anti‐tumour subtype and TAN‐2 as the pro‐tumour subset in the present study, to identify targets that might block the transition from TAN‐0 and TAN‐1 into TAN‐2 or reversal the terminal TAN‐2 state could be vital for future researches.

Finally, we developed a six‐gene prognostic and predictive model based on the neutrophil differentiation expression genes, which may provide a reference for clinical adoption. Among the six robust genes, a higher expression of CTSZ, a member of the cathepsin family, predicted better OS. CTSZ also mediates the degranulation and respiratory burst of neutrophils. In contrast, high expression of EIF3E and PPIA were involved in lipid metabolism pathways, and they were also reported to take part in the proliferation of tumour cells.^80^, ^81^ PLAUR is important in tissue rebuilding and has been shown to facilitate cancer metastasis.^82^ Accordingly, strong expression of PLAUR was found to associate with unfavourable OS and participated in negatively regulating Th1 response in the present investigation. Hence, deepening the understanding of the biological functions of these genes may pave the way for future novel therapies targeting TANs. The NDEGS model incorporating these six genes proposed relatively stable performance to predict the OS of NSCLC, which needs to be validated in future studies.

Low‐NDEGS patients harboured higher mutation rates of several genes that favour immune cell infiltration, further supported by the CIBERSORT method that the abundance of T and B lymphocytes was significantly higher while neutrophils were lower, implying a ‘hot’ TME. In contrast, the TME of high‐NDEGS is dominant by MDSCs, suggesting an immunosuppressive milieu. Low‐NDEGS group also held a higher TMB, suggesting higher immunogenicity, and may be more sensitive to immunotherapy than the high‐NDEGS group. It also conformed with the common patterns that higher neutrophil infiltration predicted treatment failure of cancers.^83^ A recent study also reported that high expression of neutrophil gene signatures was correlated with the dismal treatment benefits of ICI.^19^ In this sense, ICI plus targeting neutrophil therapies like CXCR1 and CXCR2 inhibitors, which antagonise the formation of neutrophil extracellular traps, may be feasible to reverse immunotherapy resistance.^84^, ^85^

Targeting neutrophil therapies, like impeding its recruitment to the tumour and depletion of neutrophils, have been proposed. For instance, Xue et al. recently reported that in vivo eliminating the pro‐tumour TANs, mainly CCL4+ or PDL1+ subtypes, by anti‐Ly6G antibodies attenuated liver tumour growth in mice models.^86^ On the contrary, activation of neutrophils by certain signals contributes to their tumouricidal effects and stimulates immune reactivity. For instance, Gungabeesoon et al.^87^ reported that IFN Regulatory Factor 1 was requisite for the tumour clearance effects of neutrophils. Moreover, Linde and colleagues discovered that combined therapies of CD40 agonist, tumour necrosis factor and tumour‐binding antibodies induced activation of neutrophils via C5a, which subsequently mediated oxidative damage and facilitated T cell‐independent cancer cell clearance.^88^ However, as neutrophils were heterogeneous and several subtypes proposed AP and IFN‐stimulated ability, gross elimination of neutrophils was unrecommended. Therefore, future studies should focus on developing means which quantitatively evaluate the effects of the diverse TAN subsets on anti‐tumour immunity and target a specific pro‐tumour subpopulation accordingly. Also, controlling and exploiting the subset with tumour‐killing competence may represent a great prospect. However, the fragility and low RNA content of neutrophils pose a major technical challenge to investigating them, and novel approaches are warranted.

Several strengths are worthy of the current study. First, taking advantage of the large‐scale mIF cohort (*n* = 553), we comprehensively profiled cellular composition and spatial distribution changes in TME from early to late‐stage NSCLC and uncovered spatial and temporal‐dependent prognostic effects of neutrophils. Second, scRNA‐seq on PT and paired TDLN microenvironments were applied to capture the features and differences in molecular and functional reprogramming of cells along with lymphatic metastasis. Third, diverse and continuously transitional TAN subsets were unveiled and validated by various bioinformatics analyses. Moreover, a prognostic and predictive model incorporating robust TANs differentiation‐related genes was established, showing potential for clinical application.

Simultaneously, we are aware of the limitations. First, the discrimination of TAN subtypes remains at the analytic and speculative phase based on transcriptomic data, whereas mRNA does not strictly correlate with the protein expressed. Consequently, future in vitro and in vivo experimental validation is necessary. Moreover, consisting of 2885 cells accompanied by low mRNA counts, the abundance of neutrophils was relatively lower than in prior studies, thus may lack robustness to some extent. Third, the genes used to conduct the NDEGS model were differentiation‐related rather than neutrophil‐specific. Moreover, albeit uncovering the associations between NDEGS and immunotherapeutic response, future prospective studies are needed to verify whether it could indeed predict ICI outcomes. Additionally, CD38 and CD133 used to annotate activated T cells and CSCs were not specific enough because they may also express on other cell types.

## 5 CONCLUSIONS

In brief, the cellular composition, spatial location, molecular and functional changes in PT and TDLN microenvironments along with cancer progression were deciphered, shedding light on the immunoregulatory roles and evolutionary heterogeneity of neutrophils. Our biological insights may be helpful for precision therapies by targeting rational elements in TME.

## CONFLICT OF INTEREST STATEMENT

The authors declare no potential conflict of interest.

## FUNDING INFORMATION

This work was supported by National Natural Science Foundation of China (Grant number 81871893, 62176067); Key Project of Guangzhou Scientific Research Project (Grant number 201804020030); Scientific and Technological Planning Project of Guangzhou (201903010041, 202103000040); Project Supported by Guangdong Province Universities and Colleges Pearl River Scholar Funded Scheme (2019); Cultivation of Guangdong College Students' Scientific and Technological Innovation (‘Climbing Program’ Special Funds) (Grant number pdjh2020a0480, pdjh2021a0407).

## CONSENT FOR PUBLICATION

All authors gave consent for publication.

## Supporting information


**Figure S1**. Graphical schematic of the experimental workflow and clinicopathological characteristics of samples. VPI, visceral pleural invasion; VTE, vascular tumour emboli; PT, primary tumour; LN, lymph node; NDEGS, neutrophil differentiation expression gene score.

**Figure S2**. Graphical schematic of the experimental design in segmenting tissue types and cells on multiplex immunofluorescence images. Workflow of segmenting tissue types and cells in the inForm software (A) and identifying spatial relationships between cells (B) by the StarDist deep learning algorithm.

**Figure S3**. Alterations in composition and spatial location of cells in the tumour microenvironment along with non‐small cell lung cancer (NSCLC) progression. Differences in cellular composition (A‐B) and spatial distribution (C) between early and mid‐to‐late‐stage NSCLC as evaluated by Wilcoxon t‐test. Prognostic effects of infiltrating levels of macrophages (D) and CD133+ cells (E), and spatial distances of CD4+ T cell‐CD38+ T cell pair and CD38+ T cell‐FOXP3+ T cell pair (F) in different cTNM stages as assessed by multivariate Cox regression analysis. *p < 0.05; ns, non‐significant.

**Figure S4**. Prognostic effects of cell infiltrating patterns in the primary tumours. Prognostic significance of different cell clusters in the overall group (A) and subgroups based on lung squamous cell carcinoma (LUSC) (B) and lung adenocarcinoma (LUAD) (C). Prognostic significance of cell infiltration ratio in the LUAD and LUSC subgroups (D) and overall group (E). Prognostic effects of cellular contents and spatial location of neutrophils in the overall group (F) and LUSC (G and I) and LUAD (H and J) subgroups.

**Figure S5**. Single‐cell RNA sequencing depicting the cellular composition in the primary tumour (PT) and tumour‐draining lymph node (TDLN) microenvironments. Canonical gene markers to label neutrophils in the GSE123904 (A) and E‐MTAB‐6149 (B) datasets. Classical gene markers to label cell types by UMAP plots, including GZMA for cytotoxic T cells, CD3D for T helper‐1 cells, CTLA4 for regulatory T cells, CD79A/TNFRSF13B for memory B cells, TXNDC5 for germinal centre B cells, IGHD/ TCL1A for Naive B cells, GNLY for natural killer cells, CD1C /IRF8 for dendritic cells, LYZ for macrophages, G0S2 for neutrophils, KRT8/KRT19 for epithelial cells, EMCN/ TEK for endothelial cells, COL1A2 for fibroblasts, and MS4A2/FCER1A for mast cells in PT (C), positive (D) and negative (E) TDLNs. Classification of tumour cells (MMP7/HOXB2/MOB1B/NDUFA4L2) and non‐malignant epithelial cells (KRT16/CLDN4/ACKR1) in PT (F) and tumour‐invaded TDLNs (G) by UMAP plots.

**Figure S6**. Evaluation of the purity of identified single‐cell clusters. The ROGUE approach assessed the purity of identified single‐cell populations in the positive tumour‐draining lymph nodes (TDLN) (A), negative TDLN (B), and primary tumours (PT) (C). The purity of different tumour‐associated neutrophil subclusters in positive TDLN (D), negative TDLN (E) and PT (F).

**Figure S7**. Molecular and functional reprogramming of immune cell lineages in the primary tumour (PT) and paired tumour‐draining lymph node (TDLN) microenvironments. Volcano plots displaying differentially expressed genes (DEGs) of T helper‐1 cells (Th1) (A), regulatory T cells (Treg) (E), and germinal center (GC) B cells (I) in tumour‐invaded TDLN than PT. Violin plots demonstrating the differences in expression of representative function genes of Th1 cells (B), Tregs (F) and GC B cells (J). Gene set variation analyses comparing pathway activity of Th1 cells (C), Tregs (G) and GC B cells (K) among PT, positive and negative TDLN by enrichment scores. Gene ontology analysis showing enriched biological process terms of DEGs in tumour‐invaded TDLN than PT of Th1 cells (D), Tregs (H) and GC B cells (L).

**Figure S8**. Molecular and functional reprogramming of other cell lineages in the primary tumour (PT) and paired tumour‐draining lymph node (TDLN) microenvironments. Volcano plots displaying differentially expressed genes (DEGs) of dendritic cells (DC) (A), fibroblasts (E), epithelial cells (EC) (H) and mast cells (K) in tumour‐invaded TDLN than PT. Violin plots demonstrating the differences in expression of representative function genes of DCs (B). Gene set variation analyses comparing pathway activity of DCs (C), fibroblasts (F), ECs (I) and mast cells (L) among PT, positive and negative TDLN by enrichment scores. Gene ontology analysis showing enriched biological process terms of DEGs in tumour‐invaded TDLN than PT of DCs (D), fibroblasts (G), ECs (J) and mast cells (M).

**Figure S9**. Intercellular and intracellular communication networks mediated by neutrophils in the primary tumour (PT) and paired tumour‐draining lymph node (TDLN) microenvironments. The iTALK approach evaluated the intercellular communication networks in the positive TDLN (A), negative TDLN (B) and PT (C) microenvironments by four categories: immune checkpoints, growth factors, cytokines, and others. The NicheNet analysis profiled the intracellular gene regulation effects and signal transductions of neutrophil and macrophage (D and F) and neutrophil‐endothelial cells (E and G) crosstalk.

**Figure S10**. Functional and survival analyses of important genes involved in the evolution of neutrophils based on the TCGA–LUAD cohort. Gene set variation analyses (GSVA) comparing pathway activities of featured genes in the pseudotime trajectory of neutrophils by enrichment scores (ES), including CD74 (A), HLA‐DRA (B), GBP1 (C), TNFRSF9 (D), LGALS3 (E), VEGFA (F). Kaplan–Meier curves comparing overall survival differences between high and low expression levels of the six neutrophil differentiation expression genes (NDEGs), including CTSZ (G), PLAUR (H), NME2 (I), NPM1 (J), EIF3E (K), PPIA (L). GSVA compared pathway activities among these six NDEGs by ES (M–R). *p* Values of the ANOVA test, *p < 0.05; **p < 0.01; ***p < 0.001.

**Figure S11**. Prognostic effects of neutrophil differentiation expression genes. Univariate Cox regression analysis evaluated the prognostic significance of 116 neutrophil differentiation expression genes (A). Multivariate Cox regression analysis adjusting for sex, age, Tstage and Nstage evaluated the prognostic effects of the six genes used to construct the neutrophil differentiation expression gene score model (B).

**Figure S12**. Testing the neutrophil differentiation expression gene score (NDEGS) model in the validating and entire cohorts. Kaplan–Meier curves implying the overall survival (OS) rate differences between high and low‐NDEGS groups in the validating (A) and entire (K) cohorts. Time‐dependent ROC curves and AUC values evaluate the prognostic performance of the NDEGS model at 1, 3 and 5 years in the validating (B and C) and entire (L and M) cohorts. Forest plot implying the prognostic effects of the NDEGS model in the validating (D) and entire (N) cohorts, assessed by the multivariate Cox regression analysis (D). Differences in clinicopathologic features between high and low‐NDEGS groups in the validating (E–J) and entire (O–T) cohorts.

**Data S1**. Multiplex immunofluorescence images used to train the algorithm in the inform software and the corresponding clinicopathologic characteristics of these patients.

**Table S1**. Information of reagents used in the current study.

**Table S2**. Details of primary antibodies utilised in the multiplex immunofluorescence detection.

**Table S3**. Details of Opal 7 colour multiplex immunofluorescence kit used in the present study.

**Table S4**. Information on the number of slides analysed per patient in the multiplex immunofluorescence test.

**Table S5**. Markers and corresponding cell lineages in multiplex immunofluorescence test.

**Table S6**. Details of sample used in the GSE123904 cohort from the Gene Expression Omnibus (GEO) database.

**Table S7**. Details of sample used in the E‐MTAB‐6149 cohort from the ArrayExpress database.

**Table S8**. Canonical marker genes for annotating from single‐cell RNA sequencing test on primary tumour and paired tumour‐draining lymph nodes.

**Table S9**. The gene signature of different tumour‐associated neutrophil (TAN) subsets derived from single‐cell RNA sequencing analyses.

**Table S10**. Clinicopathologic features, neutrophil differentiation expression genes score, and tumour mutational burden of patients in the TCGA–LUAD cohort.

## Data Availability

Data used to support the findings of this study are available from the corresponding author upon reasonable request.
