# Supplementary figures and images for: Cellular dynamics in tumour microenvironment along with lung cancer progression underscore spatial and evolutionary heterogeneity of neutrophil
Source: Clin Transl Med. 2023 Jul 25;13(7):e1340. doi: 10.1002/ctm2.1340 (PMC10368809; doi:10.1002/ctm2.1340)

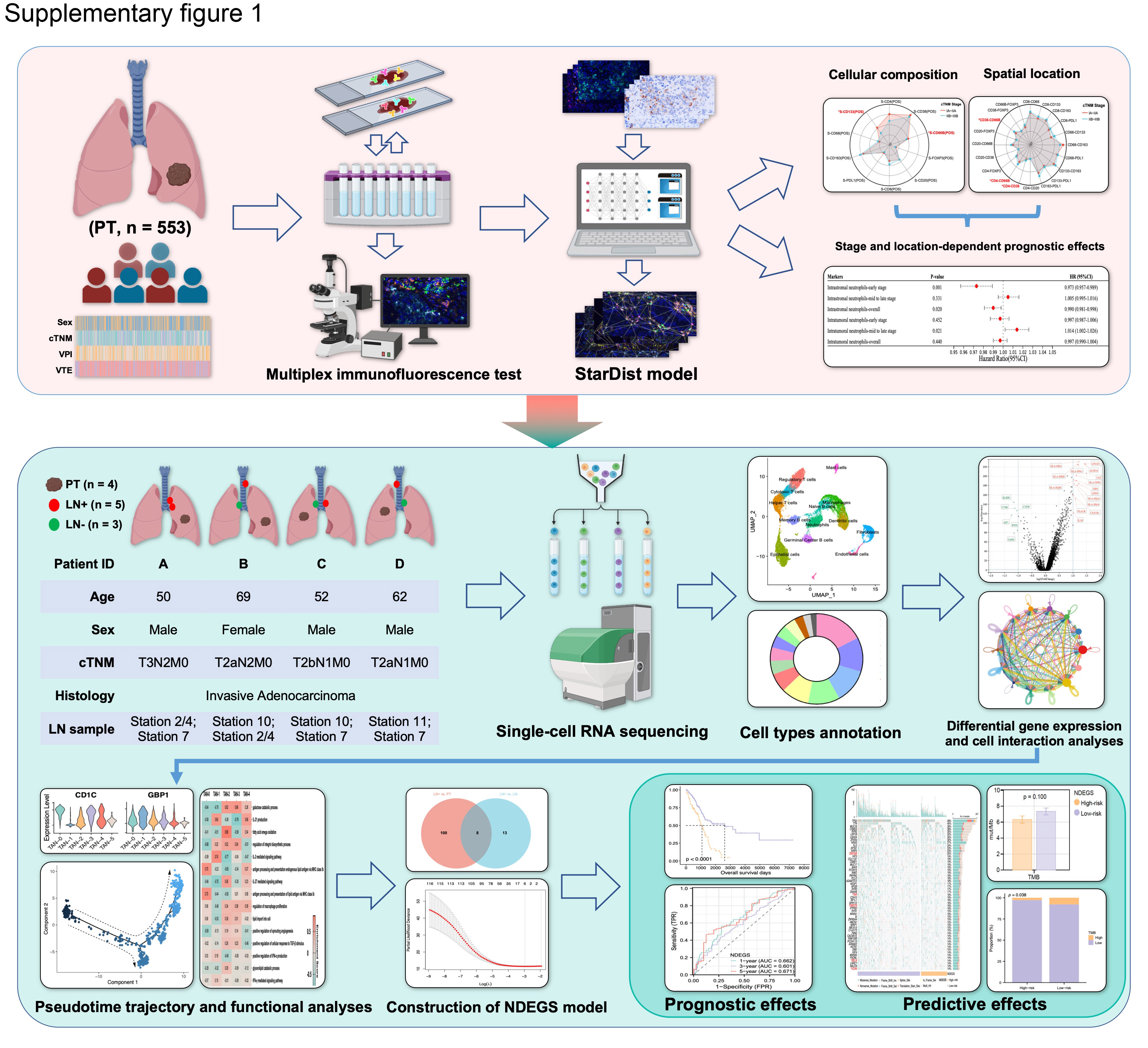

Supplement: Supplementary file 1 — Figure S1. Graphical schematic of the experimental workflow and clinicopathological characteristics of samples. VPI, visceral pleural invasion; VTE, vascular tumour emboli; PT, primary tumour; LN, lymph node; NDEGS, neutrophil differentiation expression gene score. [file CTM2-13-e1340-s017.jpg]

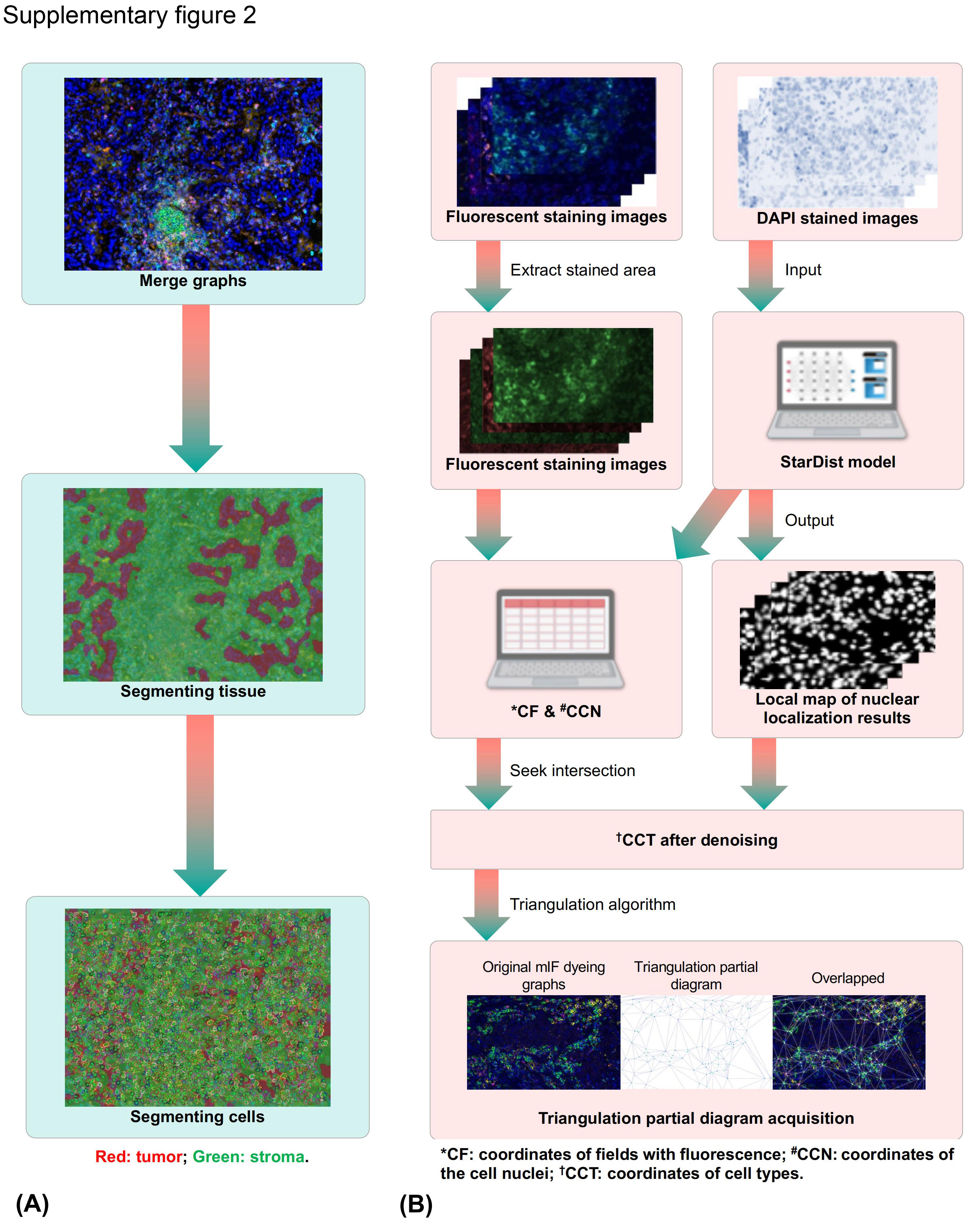

Supplement: Supplementary file 2 — Figure S2. Graphical schematic of the experimental design in segmenting tissue types and cells on multiplex immunofluorescence images. Workflow of segmenting tissue types and cells in the inForm software (A) and identifying spatial relationships between cells (B) by the StarDist deep learning algorithm. [file CTM2-13-e1340-s021.jpg]

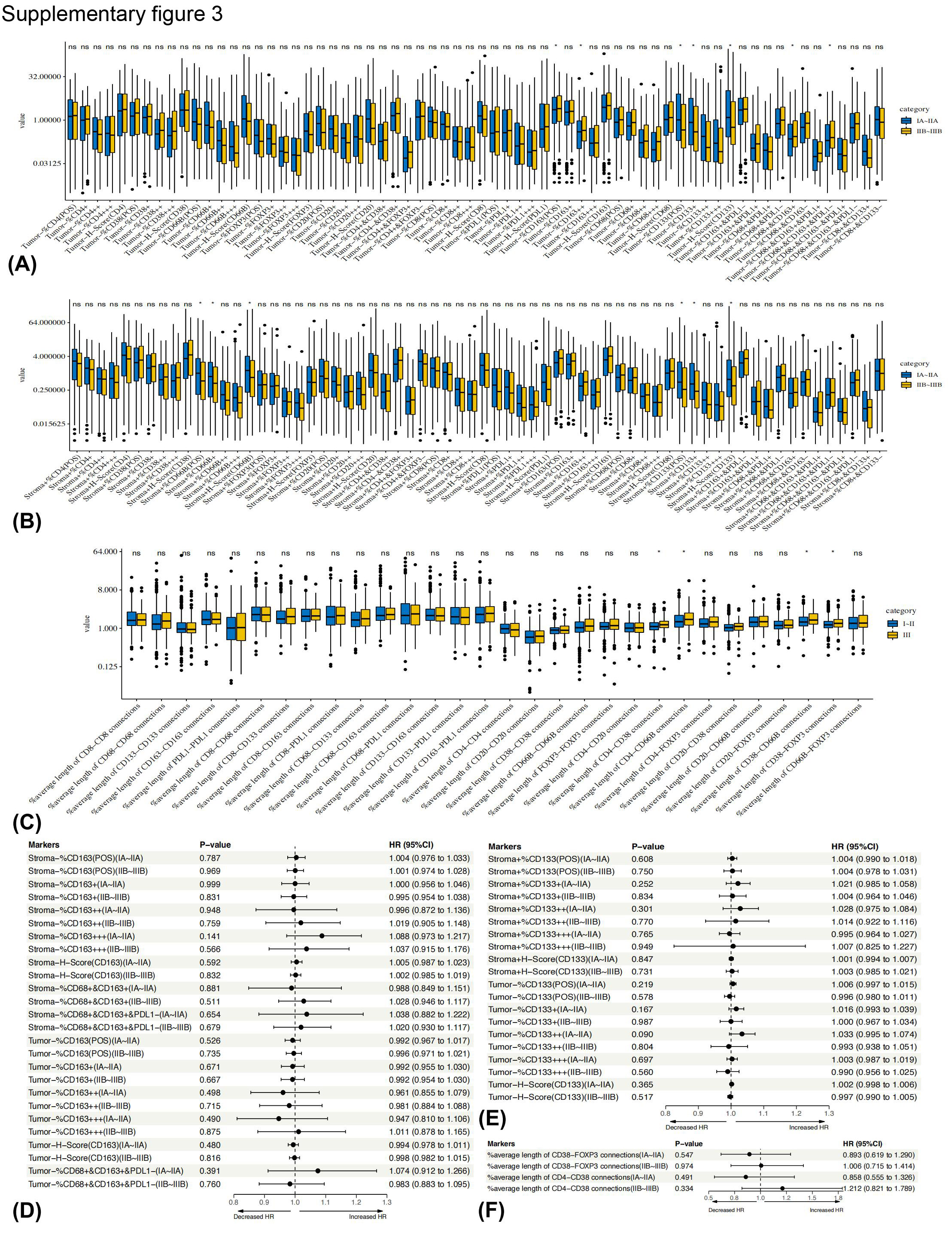

Supplement: Supplementary file 3 — Figure S3. Alterations in composition and spatial location of cells in the tumour microenvironment along with non‐small cell lung cancer (NSCLC) progression. Differences in cellular composition (A‐B) and spatial distribution (C) between early and mid‐to‐late‐stage NSCLC as evaluated by Wilcoxon t‐test. Prognostic effects of infiltrating levels of macrophages (D) and CD133+ cells (E), and spatial distances of CD4+ T cell‐CD38+ T cell pair and CD38+ T cell‐FOXP3+ T cell pair (F) in different cTNM stages as assessed by multivariate Cox regression analysis. *p < 0.05; ns, non‐significant. [file CTM2-13-e1340-s002.jpg]

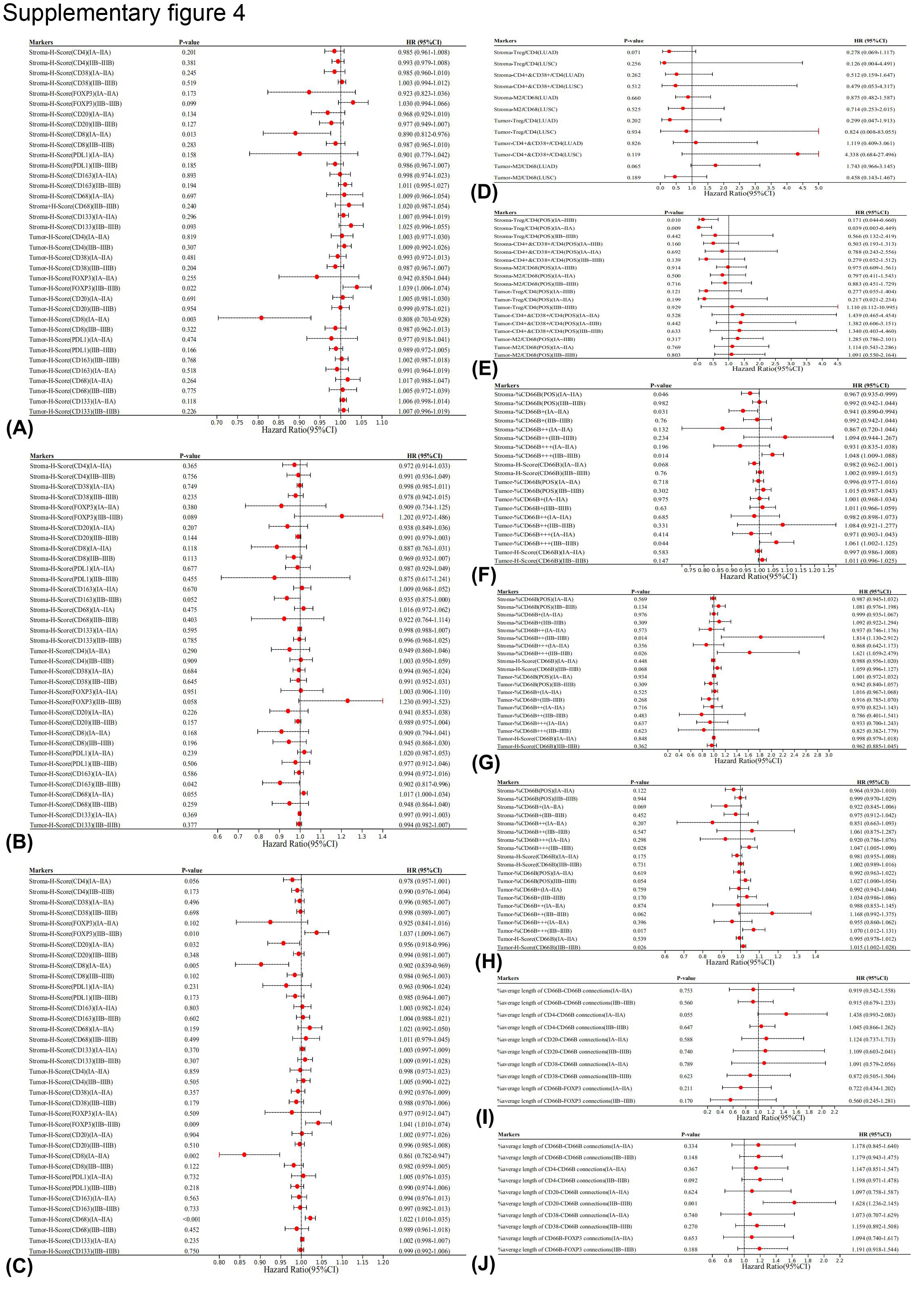

Supplement: Supplementary file 4 — Figure S4. Prognostic effects of cell infiltrating patterns in the primary tumours. Prognostic significance of different cell clusters in the overall group (A) and subgroups based on lung squamous cell carcinoma (LUSC) (B) and lung adenocarcinoma (LUAD) (C). Prognostic significance of cell infiltration ratio in the LUAD and LUSC subgroups (D) and overall group (E). Prognostic effects of cellular contents and spatial location of neutrophils in the overall group (F) and LUSC (G and I) and LUAD (H and J) subgroups. [file CTM2-13-e1340-s013.jpg]

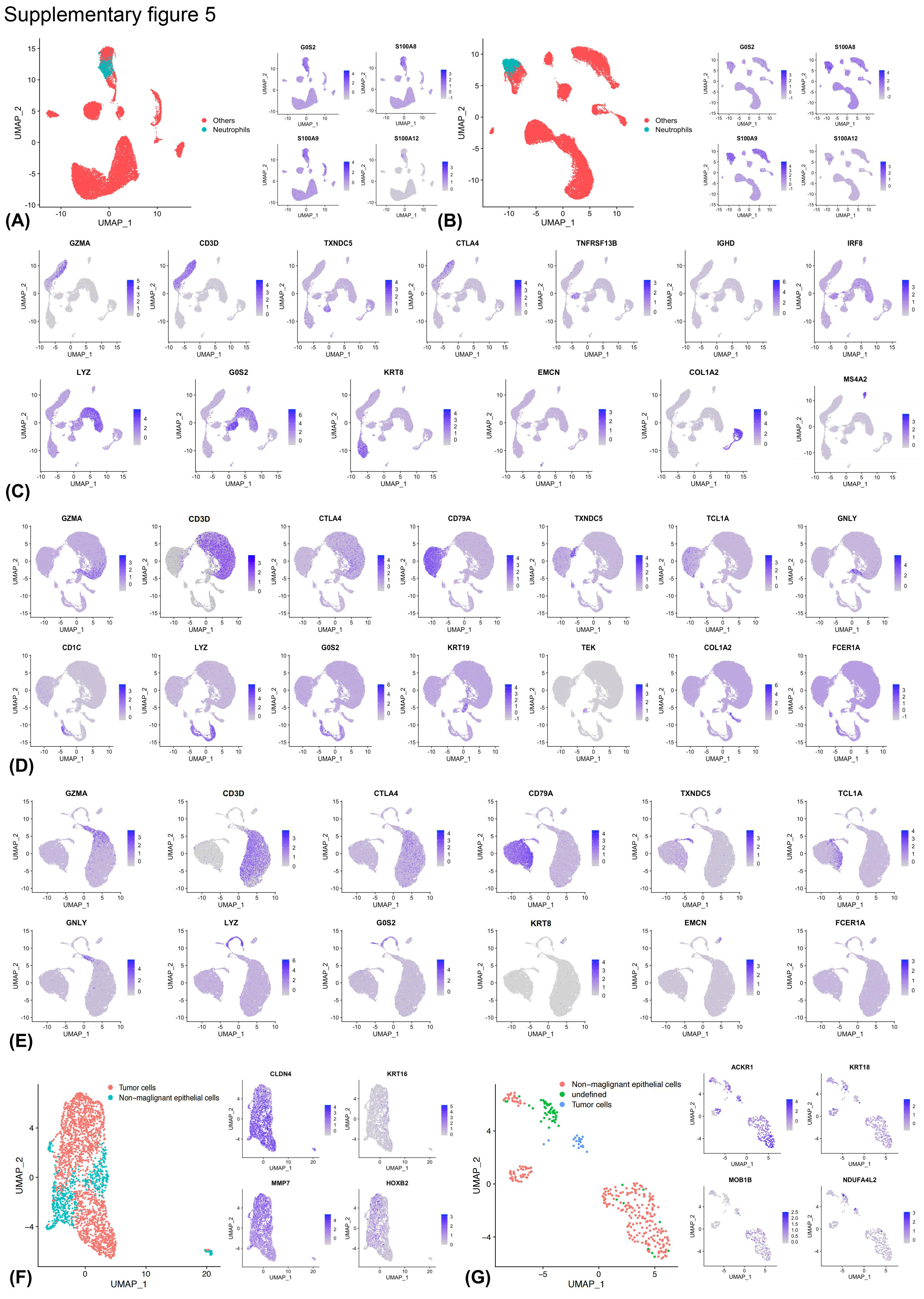

Supplement: Supplementary file 5 — Figure S5. Single‐cell RNA sequencing depicting the cellular composition in the primary tumour (PT) and tumour‐draining lymph node (TDLN) microenvironments. Canonical gene markers to label neutrophils in the GSE123904 (A) and E‐MTAB‐6149 (B) datasets. Classical gene markers to label cell types by UMAP plots, including GZMA for cytotoxic T cells, CD3D for T helper‐1 cells, CTLA4 for regulatory T cells, CD79A/TNFRSF13B for memory B cells, TXNDC5 for germinal centre B cells, IGHD/ TCL1A for Naive B cells, GNLY for natural killer cells, CD1C /IRF8 for dendritic cells, LYZ for macrophages, G0S2 for neutrophils, KRT8/KRT19 for epithelial cells, EMCN/ TEK for endothelial cells, COL1A2 for fibroblasts, and MS4A2/FCER1A for mast cells in PT (C), positive (D) and negative (E) TDLNs. Classification of tumour cells (MMP7/HOXB2/MOB1B/NDUFA4L2) and non‐malignant epithelial cells (KRT16/CLDN4/ACKR1) in PT (F) and tumour‐invaded TDLNs (G) by UMAP plots. [file CTM2-13-e1340-s004.jpg]

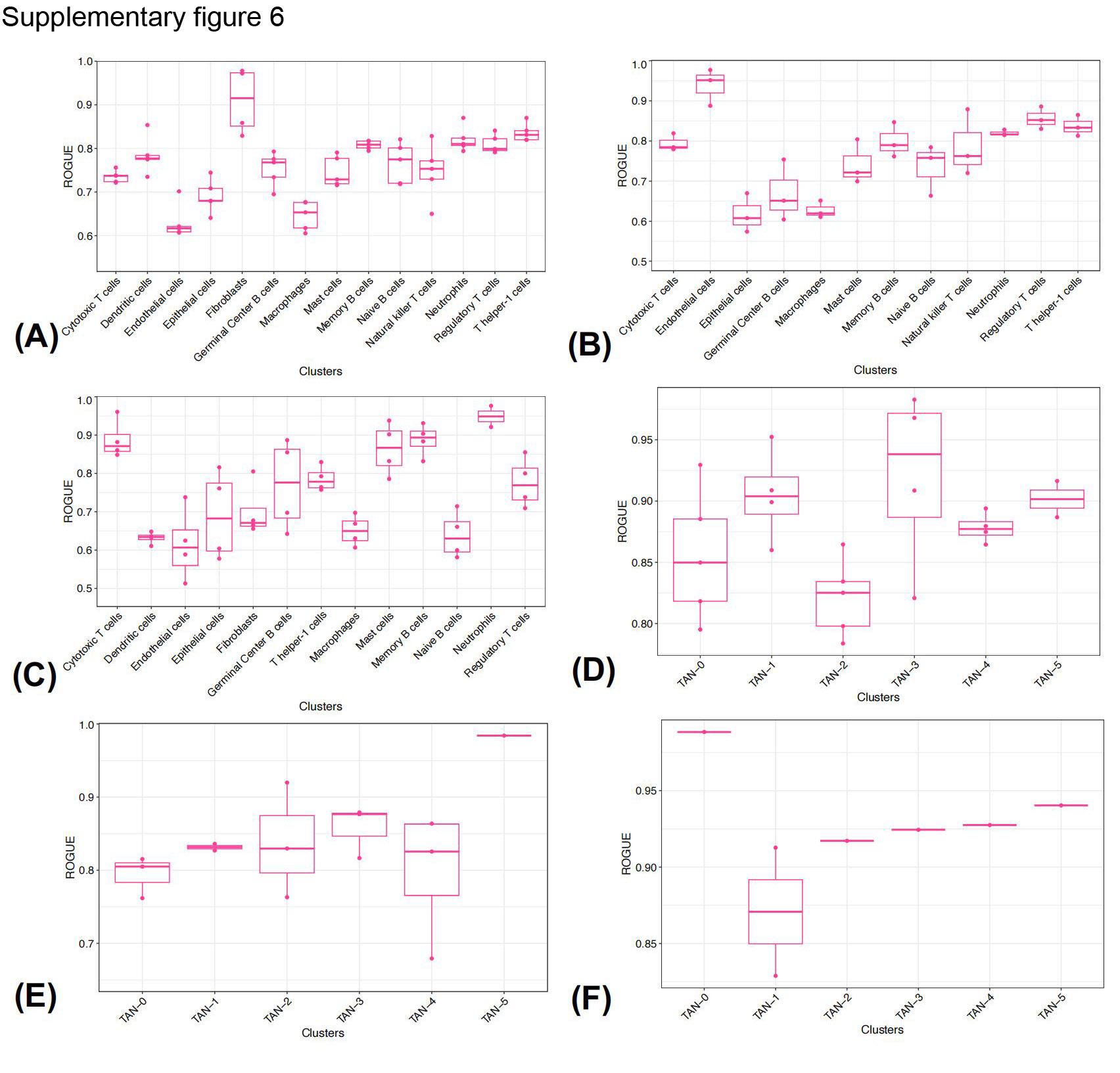

Supplement: Supplementary file 6 — Figure S6. Evaluation of the purity of identified single‐cell clusters. The ROGUE approach assessed the purity of identified single‐cell populations in the positive tumour‐draining lymph nodes (TDLN) (A), negative TDLN (B), and primary tumours (PT) (C). The purity of different tumour‐associated neutrophil subclusters in positive TDLN (D), negative TDLN (E) and PT (F). [file CTM2-13-e1340-s005.jpg]

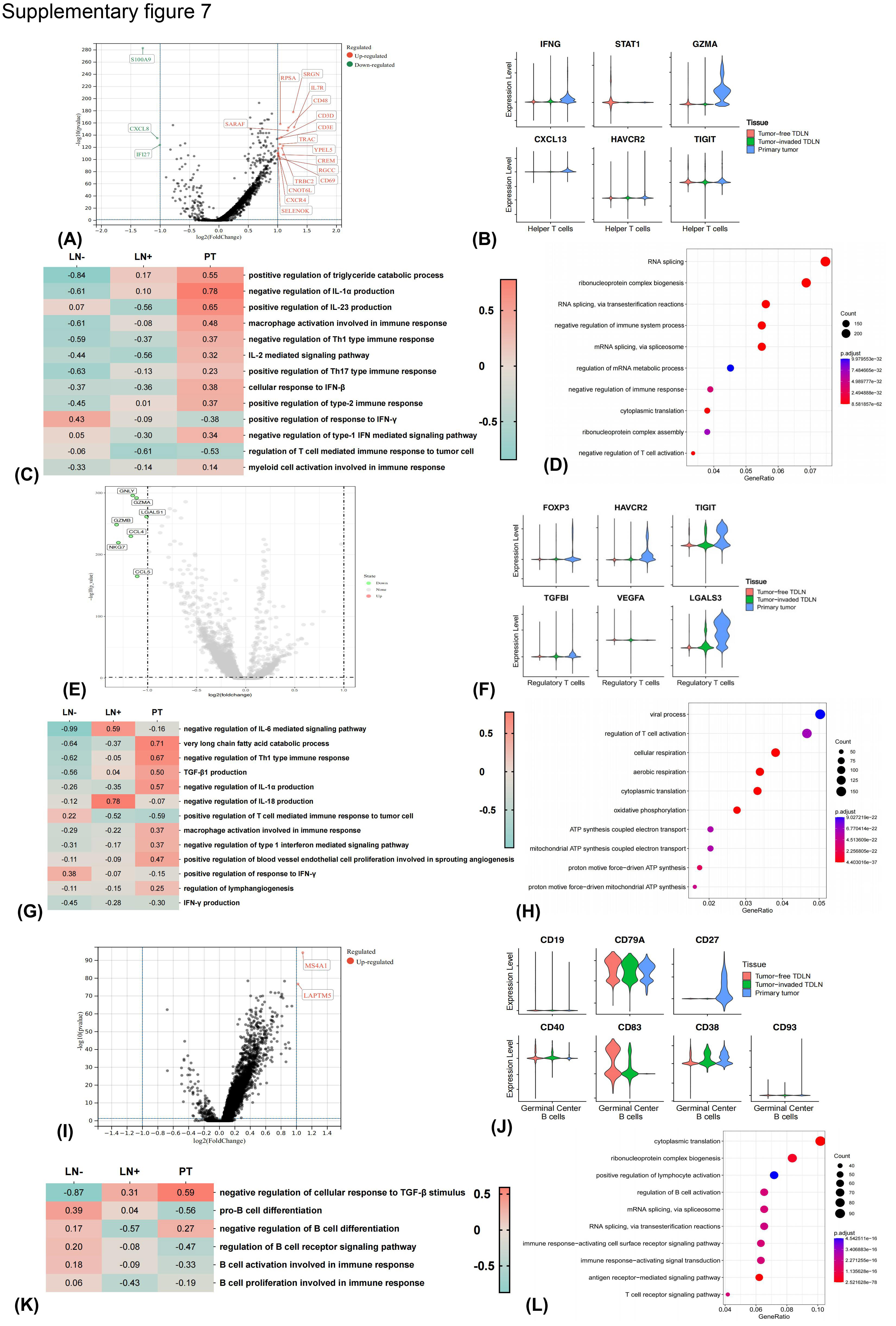

Supplement: Supplementary file 7 — Figure S7. Molecular and functional reprogramming of immune cell lineages in the primary tumour (PT) and paired tumour‐draining lymph node (TDLN) microenvironments. Volcano plots displaying differentially expressed genes (DEGs) of T helper‐1 cells (Th1) (A), regulatory T cells (Treg) (E), and germinal center (GC) B cells (I) in tumour‐invaded TDLN than PT. Violin plots demonstrating the differences in expression of representative function genes of Th1 cells (B), Tregs (F) and GC B cells (J). Gene set variation analyses comparing pathway activity of Th1 cells (C), Tregs (G) and GC B cells (K) among PT, positive and negative TDLN by enrichment scores. Gene ontology analysis showing enriched biological process terms of DEGs in tumour‐invaded TDLN than PT of Th1 cells (D), Tregs (H) and GC B cells (L). [file CTM2-13-e1340-s014.jpg]

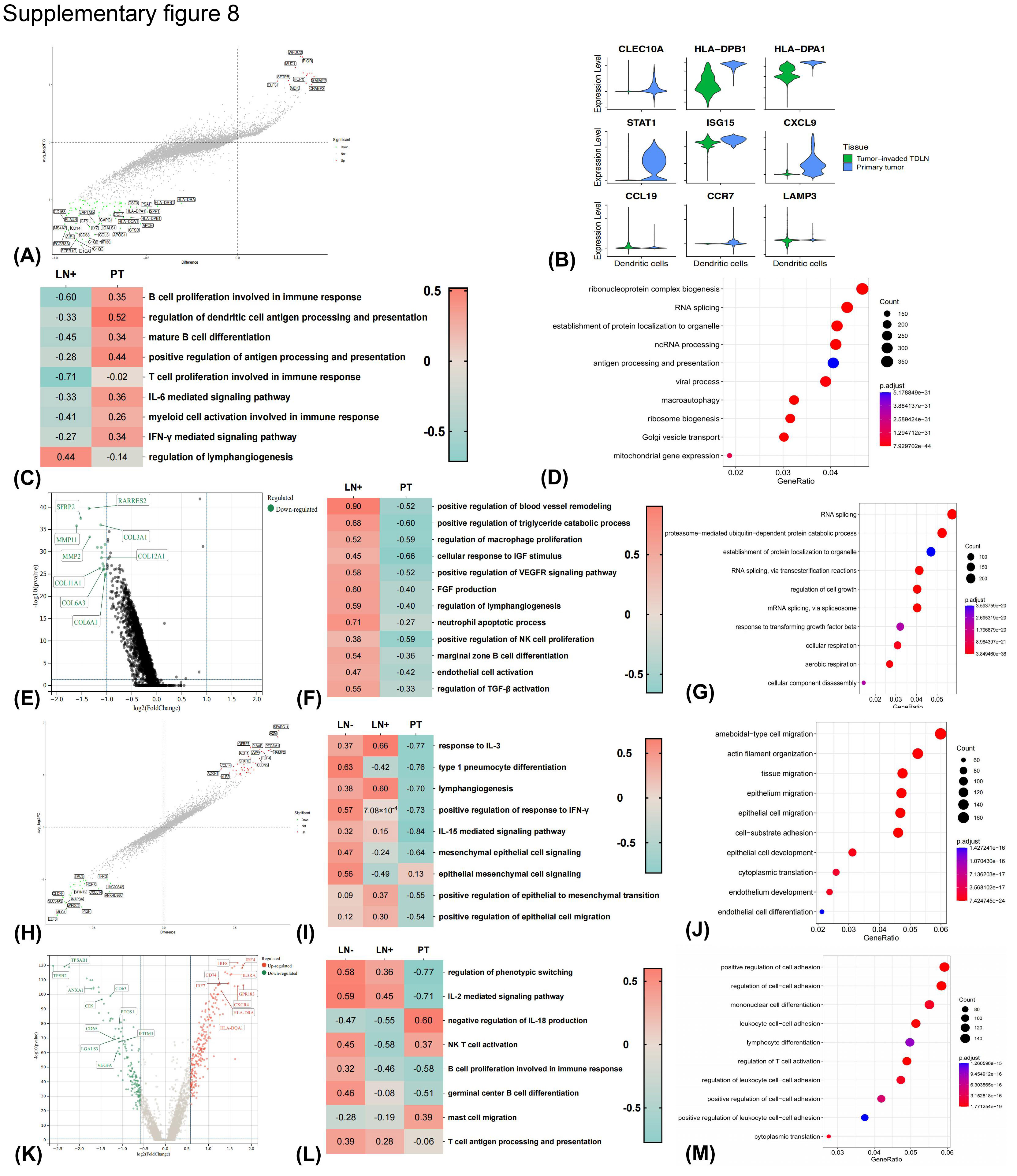

Supplement: Supplementary file 8 — Figure S8. Molecular and functional reprogramming of other cell lineages in the primary tumour (PT) and paired tumour‐draining lymph node (TDLN) microenvironments. Volcano plots displaying differentially expressed genes (DEGs) of dendritic cells (DC) (A), fibroblasts (E), epithelial cells (EC) (H) and mast cells (K) in tumour‐invaded TDLN than PT. Violin plots demonstrating the differences in expression of representative function genes of DCs (B). Gene set variation analyses comparing pathway activity of DCs (C), fibroblasts (F), ECs (I) and mast cells (L) among PT, positive and negative TDLN by enrichment scores. Gene ontology analysis showing enriched biological process terms of DEGs in tumour‐invaded TDLN than PT of DCs (D), fibroblasts (G), ECs (J) and mast cells (M). [file CTM2-13-e1340-s010.jpg]

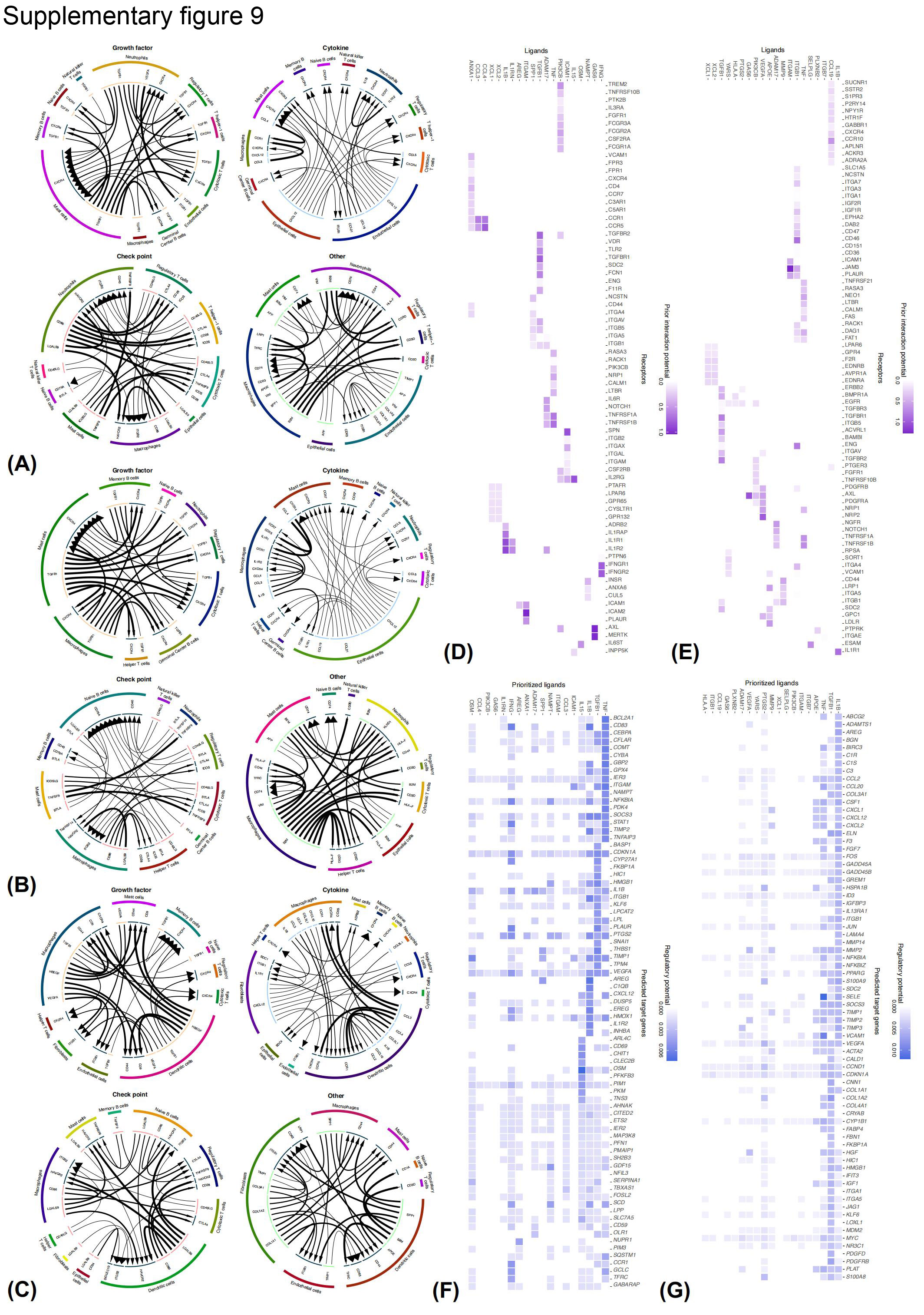

Supplement: Supplementary file 9 — Figure S9. Intercellular and intracellular communication networks mediated by neutrophils in the primary tumour (PT) and paired tumour‐draining lymph node (TDLN) microenvironments. The iTALK approach evaluated the intercellular communication networks in the positive TDLN (A), negative TDLN (B) and PT (C) microenvironments by four categories: immune checkpoints, growth factors, cytokines, and others. The NicheNet analysis profiled the intracellular gene regulation effects and signal transductions of neutrophil and macrophage (D and F) and neutrophil‐endothelial cells (E and G) crosstalk. [file CTM2-13-e1340-s018.jpg]

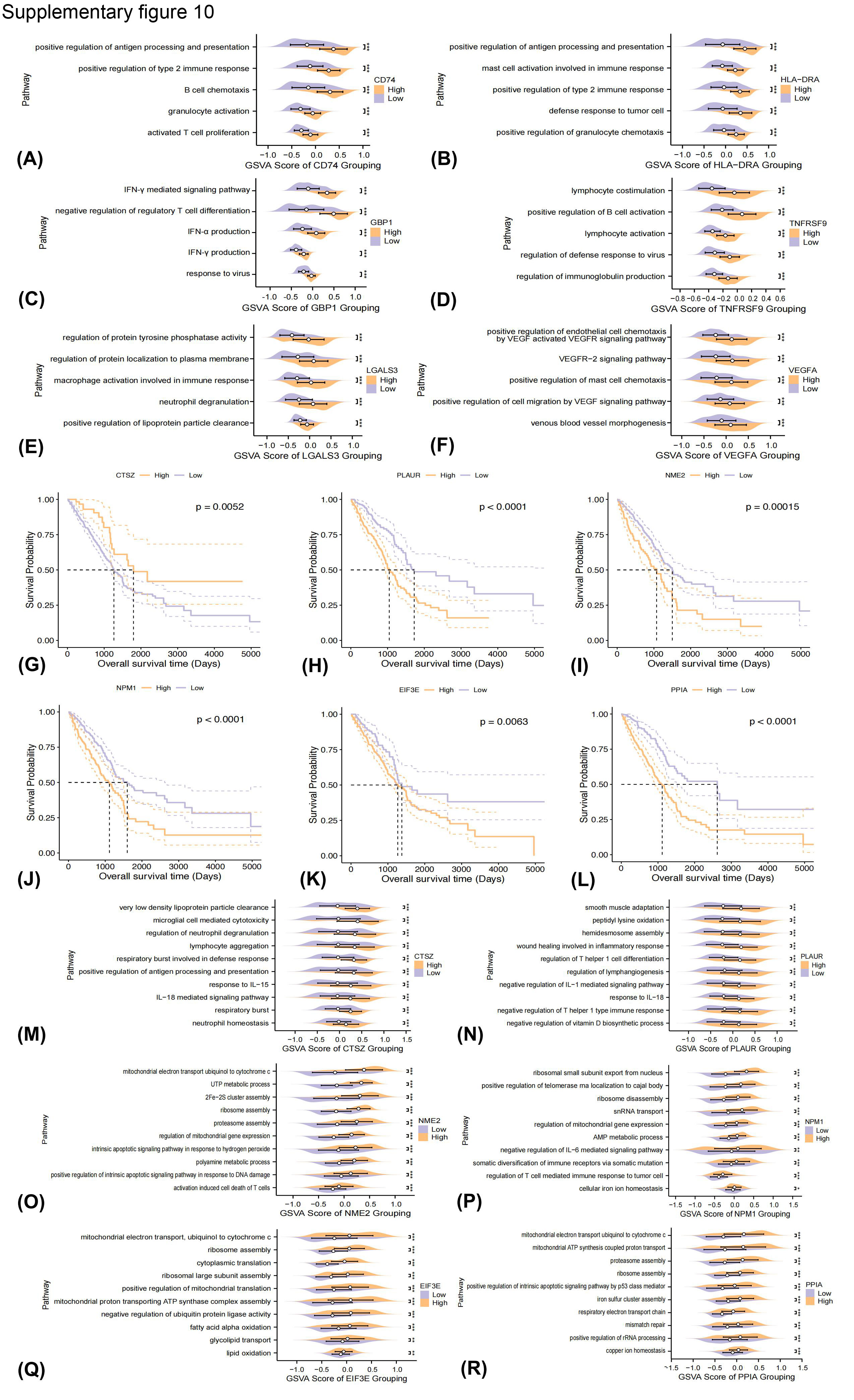

Supplement: Supplementary file 10 — Figure S10. Functional and survival analyses of important genes involved in the evolution of neutrophils based on the TCGA–LUAD cohort. Gene set variation analyses (GSVA) comparing pathway activities of featured genes in the pseudotime trajectory of neutrophils by enrichment scores (ES), including CD74 (A), HLA‐DRA (B), GBP1 (C), TNFRSF9 (D), LGALS3 (E), VEGFA (F). Kaplan–Meier curves comparing overall survival differences between high and low expression levels of the six neutrophil differentiation expression genes (NDEGs), including CTSZ (G), PLAUR (H), NME2 (I), NPM1 (J), EIF3E (K), PPIA (L). GSVA compared pathway activities among these six NDEGs by ES (M–R). p Values of the ANOVA test, *p < 0.05; **p < 0.01; ***p < 0.001. [file CTM2-13-e1340-s015.jpg]

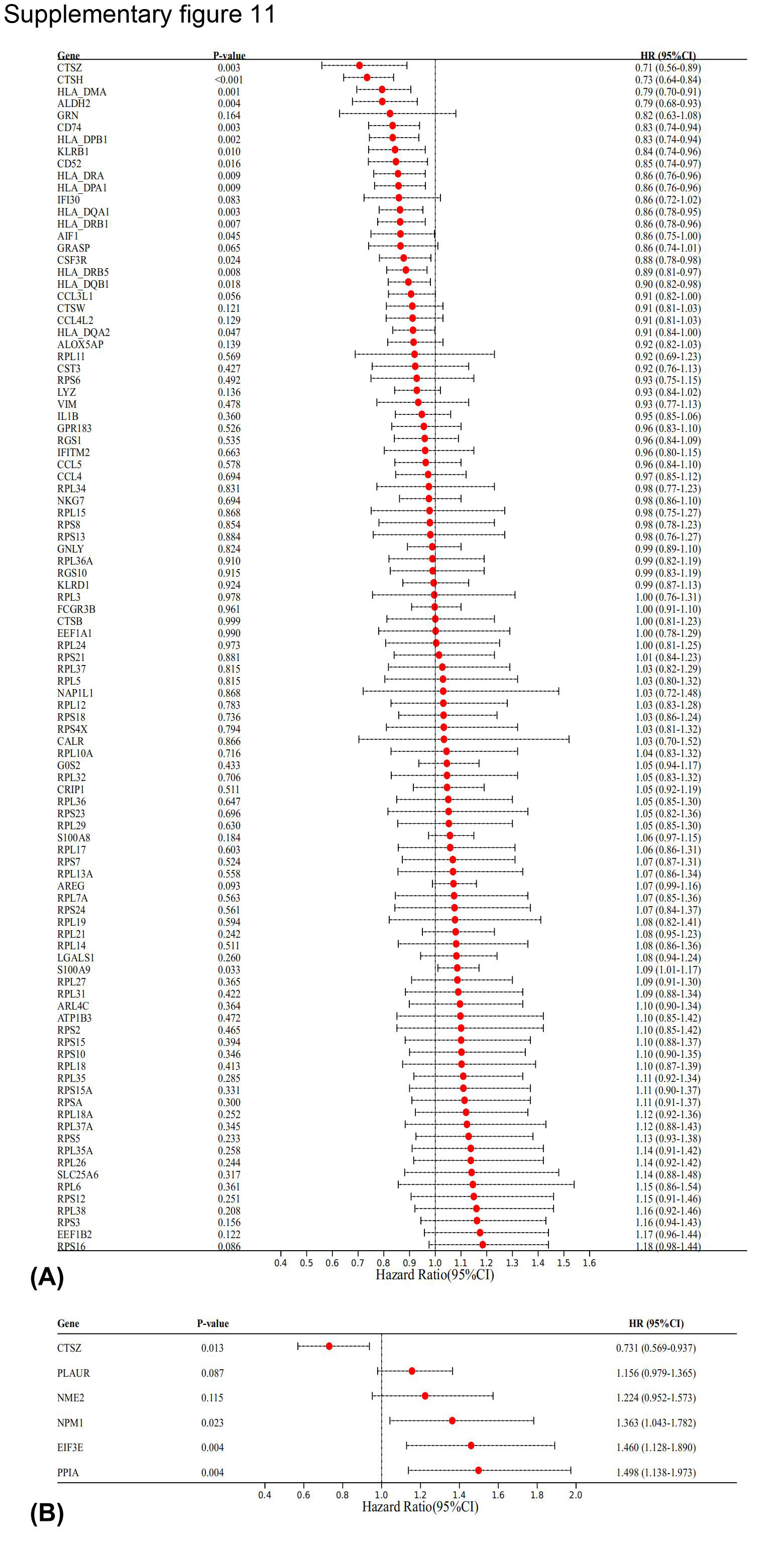

Supplement: Supplementary file 11 — Figure S11. Prognostic effects of neutrophil differentiation expression genes. Univariate Cox regression analysis evaluated the prognostic significance of 116 neutrophil differentiation expression genes (A). Multivariate Cox regression analysis adjusting for sex, age, Tstage and Nstage evaluated the prognostic effects of the six genes used to construct the neutrophil differentiation expression gene score model (B). [file CTM2-13-e1340-s003.jpg]

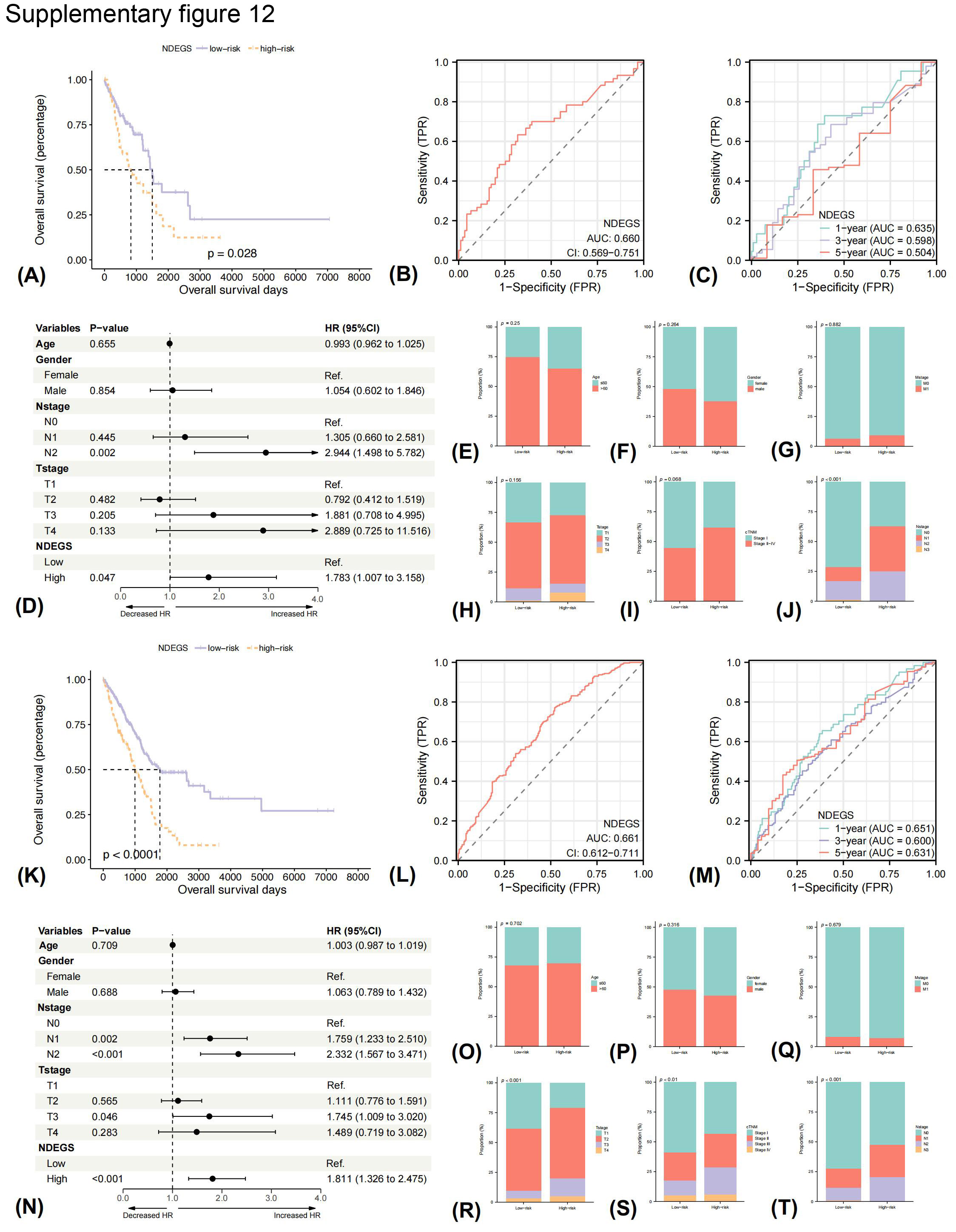

Supplement: Supplementary file 12 — Figure S12. Testing the neutrophil differentiation expression gene score (NDEGS) model in the validating and entire cohorts. Kaplan–Meier curves implying the overall survival (OS) rate differences between high and low‐NDEGS groups in the validating (A) and entire (K) cohorts. Time‐dependent ROC curves and AUC values evaluate the prognostic performance of the NDEGS model at 1, 3 and 5 years in the validating (B and C) and entire (L and M) cohorts. Forest plot implying the prognostic effects of the NDEGS model in the validating (D) and entire (N) cohorts, assessed by the multivariate Cox regression analysis (D). Differences in clinicopathologic features between high and low‐NDEGS groups in the validating (E–J) and entire (O–T) cohorts. [file CTM2-13-e1340-s009.jpg]
