# Supplementary material for: Cellular dynamics in tumour microenvironment along with lung cancer progression underscore spatial and evolutionary heterogeneity of neutrophil
Source: Clin Transl Med. 2023 Jul 25;13(7):e1340. doi: 10.1002/ctm2.1340 (PMC10368809; doi:10.1002/ctm2.1340)
Supplement: Supplementary file 14 — Table S1. Information of reagents used in the current study. [file CTM2-13-e1340-s007.docx]

**Supplementary table 1.** Information of reagents used in the current study.

| **Reagents** | **Details** | **Country** |
| --- | --- | --- |
| Tris-EDTA buffer | pH=9; Klinipath #643901 | Netherlands |
| Antibody Block/Diluent | PerkinElmer #72424205 | America |
| Anti-rabbit/mouse horseradish peroxidase antibodies | Zsbio # PV-6002 or PV-8000 | China |
| Pro-Long Diamond Antifade Mountant | Thermo Fisher | America |
