# Supplementary material for: Cellular dynamics in tumour microenvironment along with lung cancer progression underscore spatial and evolutionary heterogeneity of neutrophil
Source: Clin Transl Med. 2023 Jul 25;13(7):e1340. doi: 10.1002/ctm2.1340 (PMC10368809; doi:10.1002/ctm2.1340)
Supplement: Supplementary file 15 — Table S2. Details of primary antibodies utilised in the multiplex immunofluorescence detection. [file CTM2-13-e1340-s001.docx]

**Supplementary table 2.** Details of primary antibodies utilized in the multiplex immunofluorescence detection.

| **Marker** | **Number** | **Clone ID** | **Company** | **Dilution** |
| --- | --- | --- | --- | --- |
| **CD4** | ZM0418 | UMAB64 | Zsbio | 1:200 |
| **CD38** | ZM0422 | SPC32 | Zsbio | 1:400 |
| **CD8** | ZA-0508 | SP16 | Zsbio | 1:400 |
| **CD68** | ZM-0060 | KP1 | Zsbio | 1:400 |
| **CD20** | ab9475 | ab9475 | Zsbio | 1:100 |
| **FOXP3** | ab20034 | 236A/E7 | Zsbio | 1:100 |
| **CD163** | ZM-0428 | 10D6 | Zsbio | 1:100 |
| **CD133** | ab19898 | NA | abcam | 1:400 |
| **CD66b** | ab214175 | NA | abcam | 1:50 |
| **PD-L1** | 13684s | E1L3N | CST | 1:100 |

NA, no available for polyclonal antibody.
