# Supplementary material for: Cellular dynamics in tumour microenvironment along with lung cancer progression underscore spatial and evolutionary heterogeneity of neutrophil
Source: Clin Transl Med. 2023 Jul 25;13(7):e1340. doi: 10.1002/ctm2.1340 (PMC10368809; doi:10.1002/ctm2.1340)
Supplement: Supplementary file 16 — Table S3. Details of Opal 7 colour multiplex immunofluorescence kit used in the present study. [file CTM2-13-e1340-s023.docx]

**Supplementary table 3.** Details of Opal 7 color multiplex immunofluorescence kit used in the present study.

| **Product codes** | **Markers** |
| --- | --- |
| Opal 520 | CD20, CD163 |
| Opal 540 | CD38 |
| Opal 570 | PD-L1, CD4 |
| Opal 620 | CD8 |
| Opal 650 | CD66b, CD133 |
| Opal 690 | CD68, FOXP3 |
