# Supplementary material for: Cellular dynamics in tumour microenvironment along with lung cancer progression underscore spatial and evolutionary heterogeneity of neutrophil
Source: Clin Transl Med. 2023 Jul 25;13(7):e1340. doi: 10.1002/ctm2.1340 (PMC10368809; doi:10.1002/ctm2.1340)
Supplement: Supplementary file 18 — Table S5. Markers and corresponding cell lineages in multiplex immunofluorescence test. [file CTM2-13-e1340-s019.docx]

**Supplementary table 5.** Markers and corresponding cell lineages in multiplex immunofluorescence test.

| **Panel** | **Markers** | **Cell types** |
| --- | --- | --- |
| Panel 1 | CD4+ | CD4+ T cell |
|  | CD38+ | CD38 + T cell |
|  | CD66b+ | Neutrophil |
|  | FOXP3+ | FOXP3+ cell |
|  | CD20+ | CD20+ B cell |
|  | CD4+ CD38+ | CD4+ CD38+ T cell |
|  | CD4- CD38+ | CD4- CD38+ T cell |
|  | CD4+ FOXP3+ | CD4+ FOXP3+ Regulatory T cell |
|  | CD4+ FOXP3- | CD4+ FOXP3- T cell |
| Panel 2 | CD8+ | CD8+ T cell |
|  | PD-L1+ | PD-L1+ cell |
|  | CD163+ | CD163+ Macrophage |
|  | CD68+ | CD68+ Macrophage |
|  | CD133+ | CD133+ cell |
|  | CD8+ CD133+ | CD8+ CD133+ T cell |
|  | CD8+ CD133- | CD8+ CD133- T cell |
|  | CD68+ PD-L1+ | CD68+ PD-L1+ Macrophage |
|  | CD68+ PD-L1- | CD68+ PD-L1- Macrophage |
|  | CD163+ PD-L1+ | CD163+ PD-L1+ Macrophage |
|  | CD163+ PD-L1- | CD163+ PD-L1- Macrophage |
|  | CD68+ CD163- | M1 Macrophage |
|  | CD68+ CD163+ | M2 Macrophage |
|  | CD68+ CD163- PD-L1+ | PD-L1+ M1 Macrophage |
|  | CD68+ CD163- PD-L1- | PD-L1- M1 Macrophage |
|  | CD68+ CD163+ PD-L1+ | PD-L1+ M2 Macrophage |
|  | CD68+ CD163+ PD-L1- | PD-L1- M2 Macrophage |
