# Supplementary material for: Cellular dynamics in tumour microenvironment along with lung cancer progression underscore spatial and evolutionary heterogeneity of neutrophil
Source: Clin Transl Med. 2023 Jul 25;13(7):e1340. doi: 10.1002/ctm2.1340 (PMC10368809; doi:10.1002/ctm2.1340)
Supplement: Supplementary file 19 — Table S6. Details of sample used in the GSE123904 cohort from the Gene Expression Omnibus (GEO) database. [file CTM2-13-e1340-s022.docx]

**Supplementary table 6.** Details of sample used in the GSE123904 cohort from the Gene Expression Omnibus

(GEO) database.

| **Project ID** | **Sample ID** | **Tumor grade** | **Histological type** | **Tissue type** |
| --- | --- | --- | --- | --- |
| LUAD-003 | LUAD-003-01-1A | IA | Lung adenocarcinoma | Primary tumor |
| LUAD-003 | LUAD-003-02-1A | IA | Lung adenocarcinoma | Primary tumor |
| LUAD-003 | LUAD-003-04-1A | IV | Lung adenocarcinoma | Primary tumor |
| LUAD-003 | LUAD-003-05-1A | IA | Lung adenocarcinoma | Primary tumor |
| LUAD-003 | LUAD-003-07-1A | IIA | Lung adenocarcinoma | Primary tumor |
| LUAD-003 | LUAD-003-08-1A | IB | Lung adenocarcinoma | Primary tumor |
| LUAD-003 | LUAD-003-10-1A | IB | Lung adenocarcinoma | Primary tumor |
| LUAD-003 | LUAD-003-11-1A | IA | Lung adenocarcinoma | Primary tumor |
