# Supplementary material for: Cellular dynamics in tumour microenvironment along with lung cancer progression underscore spatial and evolutionary heterogeneity of neutrophil
Source: Clin Transl Med. 2023 Jul 25;13(7):e1340. doi: 10.1002/ctm2.1340 (PMC10368809; doi:10.1002/ctm2.1340)
Supplement: Supplementary file 20 — Table S7. Details of sample used in the E‐MTAB‐6149 cohort from the ArrayExpress database. [file CTM2-13-e1340-s012.docx]

**Supplementary table 7.** Details of sample used in the E-MTAB-6149 cohort from the ArrayExpress database.

| **Project ID** | **Sample ID** | **Sample name** | **Histological type** | **Tissue type** |
| --- | --- | --- | --- | --- |
| LUAD-004 | LUAD-004-01-1A | Sample 3a | Lung Adenocarcinoma | Primary-Tumor edge |
| LUAD-004 | LUAD-004-03-1A | Sample 3c | Lung Adenocarcinoma | Primary-Tumor core |
| LUAD-004 | LUAD-004-06-1A | Sample 4b | Lung Adenocarcinoma | Primary-Tumor edge |
| LUAD-004 | LUAD-004-08-1A | Sample 4d | Lung Adenocarcinoma | Primary-Tumor core |
| LUSC-005 | LUSC-005-01-1A | Sample 1a | Lung Squamous Cell Carcinoma | Primary-Tumor core |
| LUSC-005 | LUSC-005-03-1A | Sample 1c | Lung Squamous Cell Carcinoma | Primary-Tumor edge |
| LUSC-005 | LUSC-005-05-1A | Sample 2b | Lung Squamous Cell Carcinoma | Primary-Tumor edge |
| LUSC-005 | LUSC-005-06-1A | Sample 2c | Lung Squamous Cell Carcinoma | Primary-Tumor core |
| NSCLC-006 | NSCLC-006-01-1A | Sample 5a | Non-small Cell Lung Cancer | Primary-Tumor core |
| NSCLC-006 | NSCLC-006-02-1A | Sample 5b | Non-small Cell Lung Cancer | Primary-Tumor edge |
