# Supplementary material for: Cellular dynamics in tumour microenvironment along with lung cancer progression underscore spatial and evolutionary heterogeneity of neutrophil
Source: Clin Transl Med. 2023 Jul 25;13(7):e1340. doi: 10.1002/ctm2.1340 (PMC10368809; doi:10.1002/ctm2.1340)
Supplement: Supplementary file 21 — Table S8. Canonical marker genes for annotating from single‐cell RNA sequencing test on primary tumour and paired tumour‐draining lymph nodes. [file CTM2-13-e1340-s020.docx]

**Supplementary table 8.** Canonical marker genes for annotation of cell types from single-cell RNA sequencing test on primary tumor and paired tumor-draining lymph nodes.

| **Cell type** | **Marker gene 1** | **Marker gene 2** | **Marker gene 3** | **Clusters in PT** | **Clusters in positive TDLN** | **Clusters in negative TDLN** |
| --- | --- | --- | --- | --- | --- | --- |
| **Cytotoxic T cells** | GZMA | CD8A | CD8B | C7 | C5, C9 | C2 |
| **T helper-1 cells** | CD3D | CD40LG | ICOS | C1 | C0 | C3 |
| **Regulatory T cells** | FOXP3 | CTLA4 | TIGIT | C0 | C2 | C4 |
| **Memory B cell** | CD27 | CD79A | TNFRSF13B | C9 | C3 | C0, C5 |
| **Germinal Center B cells** | CD38 | TXNDC5 | - | C11 | C10 | C8 |
| **Naive B cells** | TCL1A | IGHD | - | C6 | C4 | C13 |
| **Natural killer cells** | GNLY | KLRD1 | - | - | C1 | C1 |
| **Dendritic cells** | CD1C | IRF8 | - | C2 | C7 | - |
| **Macrophages** | LYZ | CD68 | CD163 | C4, C15 | C6 | C6 |
| **Neutrophils** | G0S2 | S100A9 | S100A8 | C5 | C8 | C7, C10 |
| **Epithelial cells** | KRT8 | KRT19 | KRT18 | C3, C10, C12, C18 | C12 | C12 |
| **Endothelial cells** | EMCN | TEK | HAPLN1 | C14, C17 | C11 | C9 |
| **Fibroblasts** | COL1A2 | COL1A1 | COL3A1 | C8, C16 | C14 | - |
| **Mast cells** | MS4A2 | FCER1A | - | C13 | C13 | C11 |

PT, primary tumor; TDLN, tumor-draining lymph node.
