# Supplementary material for: Cellular dynamics in tumour microenvironment along with lung cancer progression underscore spatial and evolutionary heterogeneity of neutrophil
Source: Clin Transl Med. 2023 Jul 25;13(7):e1340. doi: 10.1002/ctm2.1340 (PMC10368809; doi:10.1002/ctm2.1340)
Supplement: Supplementary file 22 — Table S9. The gene signature of different tumour‐associated neutrophil (TAN) subsets derived from single‐cell RNA sequencing analyses. [file CTM2-13-e1340-s006.docx]

**Supplementary table 9.** Gene signature of different tumor-associated neutrophil (TAN) subsets derived from single-cell RNA sequencing analyses.

| **Cluster** | **Gene** | **P_val** | **Avg_log2FC** | **P_val_adj** |
| --- | --- | --- | --- | --- |
| TAN-0 | CD1C | 4.19E-74 | 1.517289415 | 8.37E-71 |
| TAN-0 | HLA-DRA | 8.29E-46 | 0.678621214 | 1.66E-42 |
| TAN-0 | HLA-DMB | 1.59E-34 | 0.661989744 | 3.17E-31 |
| TAN-0 | HLA-DPB1 | 3.18E-68 | 0.992469879 | 6.35E-65 |
| TAN-0 | CD74 | 8.19E-23 | 0.401013224 | 1.64E-19 |
| TAN-0 | FCER1A | 7.34E-47 | 1.003714792 | 1.47E-43 |
| TAN-0 | AREG | 6.51E-43 | 1.521579317 | 1.30E-39 |
| TAN-0 | CD300A | 1.46E-34 | 0.624965051 | 2.93E-31 |
| TAN-0 | CD1E | 3.35E-40 | 0.843040971 | 6.71E-37 |
| TAN-1 | TNFRSF9 | 7.48E-84 | 1.350494299 | 1.50E-80 |
| TAN-1 | GBP1 | 3.99E-10 | 0.736762707 | 7.98E-07 |
| TAN-1 | CCL17 | 2.78E-61 | 2.937665568 | 5.55E-58 |
| TAN-1 | CCL19 | 7.27E-67 | 2.973572824 | 1.45E-63 |
| TAN-1 | CCL22 | 5.51E-79 | 3.23475463 | 1.10E-75 |
| TAN-1 | IFNG | 2.92E-61 | 0.252233652 | 5.84E-58 |
| TAN-1 | CD40 | 6.02E-57 | 1.770974283 | 1.20E-53 |
| TAN-1 | IL15 | 5.25E-86 | 1.511972363 | 1.05E-82 |
| TAN-1 | IL12B | 2.45E-79 | 0.536346902 | 4.90E-76 |
| TAN-2 | CTSD | 1.96E-87 | 2.280178703 | 3.92E-84 |
| TAN-2 | CTSB | 7.19E-81 | 1.539201481 | 1.44E-77 |
| TAN-2 | LGALS3 | 1.45E-51 | 1.656007152 | 2.91E-48 |
| TAN-2 | VEGFA | 1.17E-08 | 0.436868982 | 2.33E-05 |
| TAN-2 | PSAP | 6.55E-94 | 1.843292107 | 1.31E-90 |
| TAN-2 | MMP9 | 3.94E-35 | 1.049864045 | 7.88E-32 |
| TAN-2 | IL10 | 2.80E-06 | 0.598832468 | 0.005596787 |
| TAN-2 | APOE | 6.78E-17 | 1.737942368 | 1.36E-13 |
| TAN-2 | CXCL2 | 1.58E-38 | 1.636334613 | 3.17E-35 |
| TAN-3 | FCER1A | 1.42E-17 | 0.78405571 | 2.84E-14 |
| TAN-3 | FCN1 | 5.13E-30 | 1.557884837 | 1.03E-26 |
| TAN-3 | S100A8 | 4.44E-08 | 0.600936158 | 8.87E-05 |
| TAN-3 | S100A9 | 5.99E-18 | 0.783578465 | 1.20E-14 |
| TAN-3 | MMP19 | 1.14E-16 | 0.574134176 | 2.28E-13 |
| TAN-3 | CD36 | 9.54E-19 | 0.52091138 | 1.91E-15 |
| TAN-3 | S100A4 | 7.63E-22 | 1.082118918 | 1.53E-18 |
| TAN-3 | LYZ | 1.42E-14 | 0.969540055 | 2.84E-11 |
| TAN-3 | FCN1 | 5.13E-30 | 1.557884837 | 1.03E-26 |
| TAN-4 | CXCR3 | 1.76E-27 | 0.802333589 | 3.52E-24 |
| TAN-4 | C12orf75 | 1.71E-12 | 1.117970298 | 3.42E-09 |
| TAN-4 | PLD4 | 8.24E-20 | 1.387919909 | 1.65E-16 |
| TAN-4 | CTSW | 2.88E-09 | 0.28715675 | 5.76E-06 |
| TAN-4 | IL22RA2 | 5.04E-08 | 0.38192057 | 0.000100708 |
| TAN-4 | VCAM1 | 1.04E-08 | 0.449796885 | 2.08E-05 |
| TAN-4 | HAMP | 6.17E-27 | 0.401225414 | 1.23E-23 |
| TAN-4 | CDH1 | 7.77E-17 | 0.67612905 | 1.55E-13 |
| TAN-4 | IL3RA | 1.81E-11 | 1.000141519 | 3.63E-08 |
