# Supplementary material for: Cellular dynamics in tumour microenvironment along with lung cancer progression underscore spatial and evolutionary heterogeneity of neutrophil
Source: Clin Transl Med. 2023 Jul 25;13(7):e1340. doi: 10.1002/ctm2.1340 (PMC10368809; doi:10.1002/ctm2.1340)
Supplement: Supplementary file 23 — Table S10. Clinicopathologic features, neutrophil differentiation expression genes score, and tumour mutational burden of patients in the TCGA–LUAD cohort. [file CTM2-13-e1340-s008.docx]

**Supplementary table 10.** Clinicopathologic features, neutrophil differentially expressed genes score, and tumor mutational burden of patients in the TCGA-LUAD cohort.

| **Sample ID** | **Overall survival** | **Overall survival status** | **Age** | **Gender** | **Tstage** | **Nstage** | **cTNM stage** | **NDEGS** | **NDEGS type** | **TMB** |
| --- | --- | --- | --- | --- | --- | --- | --- | --- | --- | --- |
| TCGA-49-4506-01 | 999 | 1 | 68 | female | T2 | N1 | Stage IIB | 14.66835345 | High-risk | 2.947368421 |
| TCGA-38-4632-01 | 1357 | 1 | 42 | male | T2 | N1 | Stage IV | 11.03566953 | High-risk | 12.76315789 |
| TCGA-35-4123-01 | 182 | 1 | 38 | male | T1 | N0 | Stage IA | 10.99964505 | High-risk | 6.157894737 |
| TCGA-64-1677-01 | 628 | 1 | 77 | female | T2 | N2 | Stage IIIA | 10.82236878 | High-risk | NA |
| TCGA-38-4629-01 | 864 | 1 | 68 | male | T3 | N0 | Stage IIB | 10.6323513 | High-risk | 6.684210526 |
| TCGA-50-5044-01 | 624 | 1 | 72 | female | T4 | N1 | Stage IIIB | 9.915253778 | High-risk | 3 |
| TCGA-50-5045-01 | 2174 | 1 | 57 | female | T2 | N1 | NA | 9.643613954 | High-risk | 7.578947368 |
| TCGA-64-5775-01 | 62 | 1 | 71 | male | T4 | N0 | Stage IIIA | 9.360188252 | High-risk | 11.10526316 |
| TCGA-64-1678-01 | 1189 | 1 | 70 | female | T2b | N0 | NA | 9.355209075 | High-risk | NA |
| TCGA-05-4250-01 | 121 | 1 | 79 | female | T3 | N1 | Stage IIIA | 8.898344783 | High-risk | 8.026315789 |
| TCGA-49-4486-01 | 2318 | 1 | 72 | male | T1 | N0 | Stage IA | 8.794973505 | High-risk | 3.605263158 |
| TCGA-49-4510-01 | 896 | 1 | 51 | female | T2 | N1 | Stage IIB | 8.647180044 | High-risk | 1.210526316 |
| TCGA-05-4418-01 | 274 | 1 | 69 | male | T3 | N2 | Stage IIIA | 8.629488505 | High-risk | 6.210526316 |
| TCGA-44-4112-01 | 808 | 1 | 60 | female | T2a | N0 | Stage IB | 8.572189172 | High-risk | 11.05263158 |
| TCGA-69-8253-01 | 426 | 0 | 59 | female | T1a | N1 | Stage IIA | 8.50974865 | High-risk | 3.421052632 |
| TCGA-49-4507-01 | 268 | 1 | 73 | female | T3 | N1 | Stage IIIA | 8.324132404 | High-risk | 4.605263158 |
| TCGA-50-5068-01 | 1499 | 1 | 59 | female | T2 | N1 | Stage IIB | 8.235139362 | High-risk | 2.210526316 |
| TCGA-49-4487-01 | 855 | 1 | 72 | female | T1 | N0 | Stage IA | 8.162430376 | High-risk | 8.473684211 |
| TCGA-49-4494-01 | 1081 | 1 | 77 | male | T3 | N2 | Stage IIIA | 8.151423711 | High-risk | 4.263157895 |
| TCGA-38-4631-01 | 354 | 1 | 72 | female | T2 | N0 | Stage IB | 8.119805917 | High-risk | 17.44736842 |
| TCGA-55-A493-01 | 28 | 1 | 54 | female | T2a | N0 | Stage IB | 8.099365952 | High-risk | 6.131578947 |
| TCGA-38-4625-01 | 2973 | 0 | 66 | female | T2a | N0 | Stage IB | 7.881042578 | High-risk | 13.86842105 |
| TCGA-73-4670-01 | 131 | 1 | 69 | female | T2 | N0 | Stage IV | 7.878025078 | High-risk | 8.210526316 |
| TCGA-95-A4VN-01 | 553 | 0 | 62 | female | T2a | N1 | Stage IIA | 7.874972054 | High-risk | 10.94736842 |
| TCGA-55-A490-01 | 99 | 1 | 78 | male | T2b | N0 | Stage IIA | 7.867688625 | High-risk | 30.89473684 |
| TCGA-50-6597-01 | 1268 | 1 | 79 | female | T2 | N0 | Stage IB | 7.574865487 | High-risk | 1.368421053 |
| TCGA-55-8085-01 | 904 | 0 | 64 | male | T1b | N0 | Stage IA | 7.491420705 | High-risk | 5.842105263 |
| TCGA-64-5778-01 | 1305 | 0 | 60 | male | T2 | N0 | Stage IB | 7.304748142 | High-risk | 11.02631579 |
| TCGA-78-7166-01 | 258 | 1 | 84 | male | T2 | N1 | Stage IIB | 7.304727385 | High-risk | 5 |
| TCGA-NJ-A4YG-01 | 2261 | 0 | 65 | male | T2 | N0 | Stage IB | 7.277261288 | High-risk | 3.421052632 |
| TCGA-95-8494-01 | 84 | 0 | 67 | male | T2a | N1 | Stage IIA | 7.192588321 | High-risk | 2.315789474 |
| TCGA-73-4676-01 | 281 | 1 | 45 | male | T2a | N1 | Stage IIA | 7.131981362 | High-risk | 2.131578947 |
| TCGA-55-7913-01 | 561 | 1 | 61 | female | T1b | N0 | Stage IA | 7.063525735 | High-risk | 7.947368421 |
| TCGA-67-3773-01 | 427 | 0 | 84 | female | T2 | N0 | Stage IB | 7.044409955 | High-risk | 2.473684211 |
| TCGA-50-5933-01 | 2393 | 1 | 72 | male | T4 | N2 | Stage IIIB | 7.01268026 | High-risk | 12.05263158 |
| TCGA-67-3770-01 | 610 | 0 | 70 | female | T1 | N0 | Stage IA | 6.997315589 | High-risk | 4.868421053 |
| TCGA-05-4415-01 | 91 | 1 | 57 | male | T4 | N2 | Stage IIIB | 6.878237452 | High-risk | 4.789473684 |
| TCGA-86-8672-01 | 19 | 1 | 59 | male | T3 | N0 | Stage IIB | 6.84324605 | High-risk | 6.815789474 |
| TCGA-44-2661-01 | 1159 | 0 | 69 | female | T1 | N0 | Stage IA | 6.836051453 | High-risk | 0.868421053 |
| TCGA-97-8177-01 | 499 | 0 | 59 | female | T2a | N0 | Stage IB | 6.83247055 | High-risk | 0.842105263 |
| TCGA-78-7152-01 | 1215 | 1 | 65 | male | T2 | N0 | Stage IB | 6.815676372 | High-risk | 4.631578947 |
| TCGA-69-7760-01 | 202 | 0 | 73 | male | T3 | N0 | Stage IIB | 6.70520151 | High-risk | 1.657894737 |
| TCGA-49-4488-01 | 869 | 1 | 74 | female | T1 | N0 | Stage IA | 6.661710713 | High-risk | 7.026315789 |
| TCGA-78-7535-01 | 949 | 1 | 45 | male | T2 | N0 | Stage IB | 6.616227409 | High-risk | 3.736842105 |
| TCGA-05-4426-01 | 791 | 0 | 71 | male | T2 | N0 | Stage IB | 6.58781993 | High-risk | 1.157894737 |
| TCGA-MP-A4SY-01 | 1501 | 1 | 61 | male | T2 | N1 | Stage IIB | 6.4570602 | High-risk | 4.131578947 |
| TCGA-64-1680-01 | 1126 | 0 | 63 | male | T2a | N2 | Stage IV | 6.444386592 | High-risk | 1.421052632 |
| TCGA-75-5147-01 | 1333 | 0 | NA | female | T2 | N0 | Stage IB | 6.396061996 | High-risk | 1.026315789 |
| TCGA-05-4434-01 | 457 | 1 | 67 | female | T4 | N1 | Stage IV | 6.342986541 | High-risk | 1.157894737 |
| TCGA-62-A472-01 | 910 | 0 | 70 | male | T3 | N0 | Stage IIB | 6.307487692 | High-risk | 1.394736842 |
| TCGA-73-7499-01 | 1531 | 1 | 81 | female | T2a | N0 | Stage IB | 6.284885188 | High-risk | 1.631578947 |
| TCGA-49-6745-01 | 522 | 0 | 82 | male | T2a | N2 | Stage IIIA | 6.269750219 | High-risk | 3.157894737 |
| TCGA-97-8176-01 | 468 | 1 | 63 | male | T3 | N1 | Stage IIIA | 6.198717964 | High-risk | 3.526315789 |
| TCGA-55-6972-01 | 1632 | 1 | 72 | male | T2 | N0 | Stage IB | 6.197030604 | High-risk | 6.342105263 |
| TCGA-50-6595-01 | 189 | 1 | 74 | female | T2 | N2 | Stage IIIA | 6.145536882 | High-risk | 2.447368421 |
| TCGA-49-4505-01 | 428 | 1 | 61 | female | T2 | N1 | Stage IIB | 6.114427533 | High-risk | 4.868421053 |
| TCGA-86-8359-01 | 444 | 1 | 52 | male | T3 | N2 | Stage IIIA | 6.107955168 | High-risk | 3.736842105 |
| TCGA-99-8025-01 | 1060 | 0 | 72 | female | T3 | N2 | Stage IIIA | 6.094208336 | High-risk | 11.42105263 |
| TCGA-91-6847-01 | 842 | 1 | 62 | female | T2 | N0 | Stage IB | 6.076255756 | High-risk | 1.578947368 |
| TCGA-50-5936-01 | 257 | 1 | 58 | male | T2 | N2 | Stage IIIA | 6.059833484 | High-risk | 2.789473684 |
| TCGA-05-5425-01 | 882 | 0 | 68 | male | T2b | N1 | Stage IIB | 6.044748297 | High-risk | 13.86842105 |
| TCGA-62-A46Y-01 | 414 | 1 | 70 | female | T2 | N2 | Stage IIIA | 6.025303669 | High-risk | 0.894736842 |
| TCGA-73-4659-01 | 711 | 1 | 66 | male | T2 | N2 | Stage IIIA | 6.020244442 | High-risk | 4.763157895 |
| TCGA-38-4628-01 | 1492 | 1 | 65 | female | T2 | N1 | Stage IIB | 6.00928061 | High-risk | 2.921052632 |
| TCGA-50-6594-01 | 370 | 1 | 79 | female | T3 | N2 | Stage IIIA | 5.983920986 | High-risk | 12.52631579 |
| TCGA-05-5423-01 | 151 | 0 | 65 | male | T2 | N1 | Stage IIB | 5.975595512 | High-risk | 3.605263158 |
| TCGA-J2-8194-01 | 724 | 0 | 69 | female | T3 | N0 | Stage IIB | 5.953165994 | High-risk | 3.710526316 |
| TCGA-73-4658-01 | 1600 | 1 | 80 | female | T2 | N0 | Stage IB | 5.913793627 | High-risk | 8.289473684 |
| TCGA-44-2668-01 | 761 | 1 | 51 | male | T2 | N0 | Stage IB | 5.86173685 | High-risk | 8.342105263 |
| TCGA-50-5055-01 | 1830 | 1 | 79 | female | T1 | N1 | Stage IIA | 5.836006803 | High-risk | 0.552631579 |
| TCGA-75-6212-01 | 1516 | 1 | NA | female | T2 | N1 | Stage IIB | 5.800238255 | High-risk | 0.5 |
| TCGA-05-4420-01 | 912 | 0 | 41 | male | T2 | N0 | Stage IB | 5.719103285 | High-risk | 6.5 |
| TCGA-55-6984-01 | 760 | 1 | 71 | female | T2 | N1 | Stage IIB | 5.669379975 | High-risk | 0.552631579 |
| TCGA-67-3772-01 | 573 | 0 | 82 | female | T2 | N0 | Stage IB | 5.642991357 | High-risk | NA |
| TCGA-05-4390-01 | 1126 | 0 | 58 | female | T2 | N0 | Stage IB | 5.602127409 | High-risk | 13.18421053 |
| TCGA-L9-A5IP-01 | 58 | 1 | 40 | female | T3 | N2 | Stage IV | 5.578989072 | High-risk | 6.684210526 |
| TCGA-73-4668-01 | 467 | 0 | 66 | female | T2 | N1 | Stage IIB | 5.567402983 | High-risk | 12.21052632 |
| TCGA-86-8076-01 | 993 | 0 | 42 | male | T1 | N0 | Stage IA | 5.521918915 | High-risk | 1.684210526 |
| TCGA-55-7725-01 | 442 | 0 | 68 | female | T1a | N0 | Stage IA | 5.516062098 | High-risk | 2.973684211 |
| TCGA-05-4403-01 | 578 | 0 | 76 | male | T2 | N0 | Stage IB | 5.422601537 | High-risk | 3.184210526 |
| TCGA-49-6767-01 | 677 | 0 | 46 | female | T3 | N0 | Stage IIB | 5.276810724 | High-risk | 10.68421053 |
| TCGA-91-A4BD-01 | 603 | 0 | 78 | male | T1b | N1 | Stage IIA | 5.271285924 | High-risk | 0.421052632 |
| TCGA-NJ-A55O-01 | 13 | 0 | 56 | female | T1b | N1 | Stage IIA | 5.264536593 | High-risk | 3.236842105 |
| TCGA-78-7145-01 | 826 | 1 | 52 | female | T4 | N1 | Stage IV | 5.214377591 | High-risk | 4.5 |
| TCGA-55-7281-01 | 872 | 0 | 70 | female | T1b | N0 | Stage IA | 5.20979093 | High-risk | 10.86842105 |
| TCGA-55-7227-01 | 952 | 1 | 77 | male | T3 | N1 | Stage IIIA | 5.150644837 | High-risk | 3.815789474 |
| TCGA-49-4490-01 | 385 | 1 | 45 | female | T3 | N2 | Stage IIIA | 5.120883644 | High-risk | 1.184210526 |
| TCGA-78-7158-01 | 179 | 1 | 59 | female | T4 | N2 | Stage IIIB | 5.115089613 | High-risk | 5.578947368 |
| TCGA-86-8673-01 | 862 | 0 | 61 | male | T2 | N0 | Stage IB | 5.087483022 | High-risk | 11.52631579 |
| TCGA-73-4666-01 | 800 | 0 | 52 | female | T1 | N0 | Stage IV | 5.084942253 | High-risk | 8.605263158 |
| TCGA-78-7153-01 | 3635 | 0 | 65 | female | T2 | N0 | Stage IB | 5.026674459 | High-risk | 2.973684211 |
| TCGA-MP-A4T8-01 | 161 | 1 | 68 | male | T2 | N2 | Stage IIIA | 5.013673468 | High-risk | 2.789473684 |
| TCGA-95-7567-01 | 568 | 0 | 61 | male | T2b | N1 | Stage IIB | 5.002735319 | High-risk | 21.73684211 |
| TCGA-50-6593-01 | 336 | 1 | 49 | female | T1 | N2 | Stage IIIA | 4.992452847 | High-risk | 7.394736842 |
| TCGA-55-8090-01 | 598 | 1 | 80 | male | T1a | N0 | Stage IA | 4.969171102 | High-risk | 2.026315789 |
| TCGA-50-5049-01 | 3094 | 0 | 70 | male | T2 | N0 | Stage IA | 4.93705048 | High-risk | 19.42105263 |
| TCGA-73-4677-01 | 38 | 1 | 74 | male | T2a | N0 | NA | 4.92394365 | High-risk | 5.710526316 |
| TCGA-99-8032-01 | 44 | 0 | 61 | male | T1a | N0 | Stage IA | 4.904669152 | High-risk | 9.631578947 |
| TCGA-44-2655-01 | 1324 | 0 | 65 | female | T1 | N0 | Stage IA | 4.903631563 | High-risk | 4.289473684 |
| TCGA-69-8453-01 | 813 | 0 | 77 | male | T3 | N0 | Stage IIB | 4.861628167 | High-risk | NA |
| TCGA-55-8302-01 | 478 | 0 | 54 | male | T2 | N0 | Stage IB | 4.830639509 | High-risk | 16.10526316 |
| TCGA-97-A4M3-01 | 540 | 0 | 69 | female | T1b | N0 | Stage IA | 4.816782582 | High-risk | 5 |
| TCGA-50-6590-01 | 1288 | 1 | 72 | female | T2 | N0 | Stage IB | 4.786497 | High-risk | 20.92105263 |
| TCGA-55-6979-01 | 237 | 1 | 59 | female | T2 | N1 | Stage IIB | 4.775367098 | High-risk | 3.421052632 |
| TCGA-55-6712-01 | 171 | 1 | 71 | male | T2a | N1 | Stage IIA | 4.773010536 | High-risk | 3.263157895 |
| TCGA-44-2665-01 | 1301 | 0 | 55 | female | T2 | N1 | Stage IIB | 4.739882645 | High-risk | 0.921052632 |
| TCGA-55-7903-01 | 567 | 0 | 64 | male | T1b | N0 | Stage IA | 4.732348424 | High-risk | 3.605263158 |
| TCGA-69-7974-01 | 184 | 0 | 54 | female | T2a | N2 | Stage IIIA | 4.724399429 | High-risk | 12.42105263 |
| TCGA-75-5125-01 | 2027 | 1 | NA | male | T2 | N1 | Stage IIB | 4.718493338 | High-risk | 4.157894737 |
| TCGA-38-4627-01 | 1147 | 1 | 64 | female | T1b | N1 | Stage IIA | 4.714566579 | High-risk | 0.763157895 |
| TCGA-44-7671-01 | 889 | 0 | 64 | male | T2a | N0 | Stage IB | 4.710079169 | High-risk | 5.210526316 |
| TCGA-91-6848-01 | 224 | 0 | 59 | male | T2 | N2 | Stage IIIA | 4.707273934 | High-risk | 13.73684211 |
| TCGA-55-8301-01 | 534 | 0 | 58 | male | T2a | N0 | Stage IB | 4.706883919 | High-risk | 6.815789474 |
| TCGA-05-4430-01 | 761 | 0 | 59 | female | T2 | N0 | Stage IB | 4.685178636 | High-risk | 6.526315789 |
| TCGA-75-6214-01 | 1115 | 1 | NA | female | T2 | N2 | Stage IIIA | 4.678390952 | High-risk | 16.81578947 |
| TCGA-4B-A93V-01 | 300 | 1 | 52 | female | T1b | N0 | Stage IA | 4.675803579 | High-risk | 3.236842105 |
| TCGA-86-8585-01 | 353 | 0 | 57 | male | T2a | N0 | Stage IB | 4.646307806 | High-risk | 13.86842105 |
| TCGA-91-8496-01 | 505 | 0 | 63 | female | T2a | NX | Stage IB | 4.633232706 | High-risk | 0.815789474 |
| TCGA-55-6982-01 | 995 | 1 | 79 | female | T2 | N1 | Stage IIB | 4.614077228 | High-risk | 5.105263158 |
| TCGA-L9-A443-01 | 193 | 1 | 63 | female | T1a | N0 | Stage IA | 4.585246871 | High-risk | 6.131578947 |
| TCGA-78-7537-01 | 1622 | 1 | 72 | male | T2 | N0 | Stage IB | 4.575619312 | High-risk | 3.473684211 |
| TCGA-MP-A4TI-01 | 429 | 1 | 72 | male | T2a | N1 | Stage IIA | 4.525607903 | High-risk | 5.315789474 |
| TCGA-44-7661-01 | 557 | 1 | 69 | female | T2a | N0 | Stage IB | 4.506525451 | High-risk | 6.394736842 |
| TCGA-55-6970-01 | 464 | 1 | 67 | female | T2 | N2 | Stage IIIA | 4.478719174 | High-risk | 4.289473684 |
| TCGA-MP-A4T7-01 | 167 | 1 | 75 | female | T2 | N0 | Stage IV | 4.468980121 | High-risk | 3.263157895 |
| TCGA-44-2662-01 | 1280 | 0 | 65 | male | T2 | N0 | Stage IB | 4.445465513 | High-risk | 4.763157895 |
| TCGA-55-7911-01 | 537 | 0 | 70 | female | T1a | N0 | Stage IA | 4.431475758 | High-risk | 5.315789474 |
| TCGA-91-6836-01 | 417 | 0 | 52 | female | T2 | N0 | Stage IB | 4.416549235 | High-risk | 10.63157895 |
| TCGA-55-A491-01 | 626 | 0 | 81 | female | T1b | N0 | Stage IA | 4.408575359 | High-risk | 8.710526316 |
| TCGA-78-8660-01 | 321 | 1 | 69 | male | T2 | N1 | Stage IIB | 4.403823321 | High-risk | 5.736842105 |
| TCGA-67-6215-01 | 174 | 0 | 52 | female | T2a | N0 | Stage IB | 4.400560249 | High-risk | 2.657894737 |
| TCGA-55-7283-01 | 609 | 0 | 76 | female | T3 | N2 | Stage IIIA | 4.345323631 | High-risk | 5.394736842 |
| TCGA-97-8175-01 | 551 | 0 | 55 | female | T2a | N0 | Stage IB | 4.340995608 | High-risk | 0.710526316 |
| TCGA-78-7154-01 | 593 | 1 | 72 | male | T3 | N2 | Stage IIIA | 4.308705359 | High-risk | 5.184210526 |
| TCGA-44-2659-01 | 1367 | 0 | 65 | female | T1 | N1 | Stage IIB | 4.291739834 | High-risk | 10.23684211 |
| TCGA-55-8205-01 | 599 | 0 | 76 | female | T2b | N0 | Stage IIA | 4.291343475 | High-risk | 12.34210526 |
| TCGA-55-8299-01 | 469 | 1 | 61 | female | T1b | N0 | Stage IA | 4.289269239 | High-risk | 5.052631579 |
| TCGA-44-6777-01 | 987 | 1 | 85 | female | T2 | NX | Stage IB | 4.288978414 | High-risk | 8.894736842 |
| TCGA-50-5072-01 | 250 | 1 | 74 | male | T2 | N2 | Stage IIIA | 4.280898637 | High-risk | 3.921052632 |
| TCGA-44-7667-01 | 1097 | 0 | 49 | female | T3 | N0 | Stage IIB | 4.254903244 | High-risk | 11.15789474 |
| TCGA-78-7146-01 | 173 | 1 | 71 | female | T2 | N2 | Stage IIIA | 4.24716911 | High-risk | 8.263157895 |
| TCGA-91-8499-01 | 36 | 0 | 76 | female | T1b | N0 | Stage IA | 4.243335919 | High-risk | 7.289473684 |
| TCGA-MP-A4TD-01 | 307 | 1 | 71 | male | T2 | N2 | Stage IIIA | 4.240588815 | High-risk | 1.815789474 |
| TCGA-05-5429-01 | 275 | 1 | 60 | male | T3 | N2 | Stage IIIA | 4.20911404 | High-risk | 0.868421053 |
| TCGA-49-AARE-01 | 1229 | 1 | 51 | female | T1 | N0 | Stage IA | 4.18271724 | High-risk | 31.13157895 |
| TCGA-62-A46U-01 | 2067 | 0 | 71 | female | T2 | N1 | Stage IIB | 4.131334211 | High-risk | 1.026315789 |
| TCGA-67-6216-01 | 141 | 0 | 57 | female | T1a | N0 | Stage IA | 4.114954047 | Low-risk | 0.973684211 |
| TCGA-49-AAQV-01 | 677 | 1 | 63 | female | T1 | N1 | Stage II | 4.109193756 | Low-risk | 1.763157895 |
| TCGA-99-8033-01 | 656 | 1 | 74 | female | TX | NX | Stage IV | 4.09137354 | Low-risk | 4.289473684 |
| TCGA-55-6978-01 | 176 | 1 | 81 | male | T2b | N0 | Stage IIA | 4.091239971 | Low-risk | 0.710526316 |
| TCGA-64-1679-01 | 2488 | 0 | 58 | female | T1 | N2 | Stage IIIA | 4.084512562 | Low-risk | 12.13157895 |
| TCGA-44-3396-01 | 1130 | 0 | 74 | female | T2 | N2 | Stage IIIA | 4.081428471 | Low-risk | 4.736842105 |
| TCGA-49-4501-01 | 1421 | 1 | 67 | female | T2 | N0 | Stage IB | 4.076735336 | Low-risk | 1.184210526 |
| TCGA-MP-A4TC-01 | 74 | 1 | 77 | male | T1 | N2 | Stage IIIA | 4.068655162 | Low-risk | 5.947368421 |
| TCGA-49-AAR3-01 | 1893 | 0 | 69 | male | T2 | N1 | Stage IIB | 4.057299684 | Low-risk | 3.368421053 |
| TCGA-49-4512-01 | 905 | 1 | 69 | female | T2 | N2 | Stage IIIA | 4.03479048 | Low-risk | 0.973684211 |
| TCGA-44-A47G-01 | 351 | 0 | 73 | female | T1 | N0 | Stage IA | 4.006998299 | Low-risk | 2.026315789 |
| TCGA-MP-A4TK-01 | 582 | 1 | 56 | female | T2 | N1 | Stage IIB | 3.994367486 | Low-risk | 8.184210526 |
| TCGA-93-A4JQ-01 | 526 | 0 | 49 | male | T1b | N0 | Stage IA | 3.967371882 | Low-risk | 2.710526316 |
| TCGA-73-4675-01 | 922 | 1 | 59 | male | T3 | N1 | Stage IIIA | 3.964390982 | Low-risk | 1.631578947 |
| TCGA-95-7039-01 | 1272 | 0 | 54 | female | T3 | N0 | Stage IIB | 3.95366106 | Low-risk | 29.94736842 |
| TCGA-55-6543-01 | 435 | 0 | 60 | female | T1b | N0 | Stage IA | 3.920739517 | Low-risk | 0.578947368 |
| TCGA-L9-A743-01 | 664 | 0 | 56 | male | T2a | N1 | Stage IIA | 3.911442199 | Low-risk | 3.026315789 |
| TCGA-86-8669-01 | 938 | 0 | 64 | male | T1b | N0 | Stage IA | 3.895445212 | Low-risk | 3.052631579 |
| TCGA-55-1595-01 | 1479 | 0 | 74 | female | T1 | N0 | Stage IA | 3.850831638 | Low-risk | 7.473684211 |
| TCGA-95-7562-01 | 87 | 1 | 71 | male | T2a | N1 | Stage IIA | 3.828687195 | Low-risk | 10.07894737 |
| TCGA-69-7763-01 | 690 | 0 | 69 | male | T1b | N0 | Stage IA | 3.801793954 | Low-risk | 1.684210526 |
| TCGA-75-7027-01 | 3059 | 0 | NA | male | T2 | N0 | Stage IB | 3.755958288 | Low-risk | 5.315789474 |
| TCGA-55-8094-01 | 541 | 0 | 51 | male | T2b | N0 | Stage IV | 3.750545841 | Low-risk | 10.34210526 |
| TCGA-55-8207-01 | 977 | 0 | 73 | male | T2a | N0 | Stage IB | 3.738451868 | Low-risk | 7.236842105 |
| TCGA-55-7726-01 | 652 | 0 | 72 | female | T1b | N0 | Stage IA | 3.728732396 | Low-risk | 2.815789474 |
| TCGA-78-7542-01 | 321 | 1 | 56 | male | T2 | N0 | Stage IB | 3.706403069 | Low-risk | 8.5 |
| TCGA-55-A48Y-01 | 630 | 0 | 69 | male | T2b | N0 | Stage IIA | 3.704527714 | Low-risk | 8 |
| TCGA-86-A4JF-01 | 737 | 1 | 56 | male | T3 | N0 | Stage IIB | 3.703591474 | Low-risk | 31.78947368 |
| TCGA-62-A46O-01 | 1454 | 1 | 65 | female | T2 | N0 | Stage IB | 3.676761337 | Low-risk | 20.57894737 |
| TCGA-50-5941-01 | 1474 | 0 | 55 | female | T2a | N2 | Stage IIIA | 3.665908403 | Low-risk | 9.105263158 |
| TCGA-50-6592-01 | 777 | 1 | 71 | female | T2 | N0 | Stage IB | 3.616161669 | Low-risk | 6.736842105 |
| TCGA-05-4417-01 | 455 | 0 | 51 | female | T2 | N0 | Stage IB | 3.572052595 | Low-risk | 6.763157895 |
| TCGA-05-4425-01 | 669 | 0 | 70 | female | T2 | N0 | Stage IV | 3.567432787 | Low-risk | 1 |
| TCGA-MP-A4T4-01 | 2617 | 1 | 68 | female | T2 | N1 | Stage IIB | 3.533196874 | Low-risk | 9.289473684 |
| TCGA-86-A4D0-01 | 116 | 1 | 48 | male | T2b | N0 | Stage IIA | 3.52826667 | Low-risk | NA |
| TCGA-55-7910-01 | 1040 | 0 | 50 | female | T2b | N0 | Stage IIA | 3.506300075 | Low-risk | 7.473684211 |
| TCGA-97-8547-01 | 657 | 0 | 78 | female | T2a | N2 | Stage IIIA | 3.499103029 | Low-risk | 1.394736842 |
| TCGA-44-6774-01 | 658 | 0 | 56 | female | T1 | N2 | Stage IIIA | 3.469398359 | Low-risk | 6.815789474 |
| TCGA-53-7813-01 | 424 | 0 | 51 | female | T4 | N0 | Stage IIIB | 3.468905947 | Low-risk | 3.763157895 |
| TCGA-49-AAR0-01 | 4765 | 0 | 57 | male | T1 | N0 | Stage IA | 3.462006073 | Low-risk | 3.157894737 |
| TCGA-44-2666-01 | 97 | 1 | 43 | male | T2 | N0 | Stage IB | 3.448578698 | Low-risk | 0.947368421 |
| TCGA-44-8117-01 | 385 | 0 | 54 | female | T2a | N0 | Stage IB | 3.428270274 | Low-risk | 17.57894737 |
| TCGA-78-7148-01 | 626 | 1 | 71 | male | T2 | N1 | Stage IIB | 3.414883399 | Low-risk | 5.315789474 |
| TCGA-78-7149-01 | 3940 | 0 | 71 | male | T4 | N0 | Stage IIIB | 3.381388008 | Low-risk | 3.578947368 |
| TCGA-MP-A4TF-01 | 336 | 1 | 58 | female | T2b | N0 | Stage IIA | 3.3773712 | Low-risk | 9.842105263 |
| TCGA-78-7167-01 | 2681 | 1 | 77 | male | T2 | N0 | Stage IV | 3.36272243 | Low-risk | 2.631578947 |
| TCGA-86-8674-01 | 806 | 0 | 50 | male | T2a | N1 | Stage IIA | 3.336462445 | Low-risk | 6.973684211 |
| TCGA-55-7907-01 | 343 | 1 | 77 | male | T2a | N1 | Stage IIA | 3.328705626 | Low-risk | 27.42105263 |
| TCGA-38-4626-01 | 3674 | 0 | 57 | female | T2b | N0 | NA | 3.319390993 | Low-risk | 6.157894737 |
| TCGA-69-7765-01 | 165 | 0 | 56 | male | T4 | N0 | NA | 3.305962392 | Low-risk | 14.13157895 |
| TCGA-86-8055-01 | 124 | 1 | 79 | male | T2a | N1 | Stage IIA | 3.300987205 | Low-risk | 1.078947368 |
| TCGA-93-7347-01 | 683 | 0 | 76 | female | T1a | N0 | Stage IA | 3.286288932 | Low-risk | 2.736842105 |
| TCGA-MN-A4N1-01 | 827 | 0 | 60 | male | T2a | N1 | Stage IIA | 3.284326161 | Low-risk | 10.63157895 |
| TCGA-78-7147-01 | 586 | 1 | 67 | female | T2 | N1 | Stage IIB | 3.28393912 | Low-risk | 7.763157895 |
| TCGA-55-A48Z-01 | 651 | 0 | 60 | female | T1a | N3 | Stage IIIB | 3.279086749 | Low-risk | 1.131578947 |
| TCGA-55-6969-01 | 1239 | 0 | 52 | male | T2 | N0 | Stage IB | 3.215204218 | Low-risk | 9.868421053 |
| TCGA-44-A47A-01 | 466 | 0 | 78 | female | T2a | N0 | Stage IB | 3.181564984 | Low-risk | 5.421052632 |
| TCGA-50-6673-01 | 22 | 1 | 84 | female | T1 | N0 | Stage I | 3.15918508 | Low-risk | 1.342105263 |
| TCGA-97-7937-01 | 564 | 0 | 65 | male | T2a | N0 | Stage IB | 3.12360707 | Low-risk | 11.47368421 |
| TCGA-05-4389-01 | 1369 | 0 | 70 | male | T1 | N0 | Stage IA | 3.090871346 | Low-risk | 5.342105263 |
| TCGA-97-A4M5-01 | 634 | 0 | 83 | male | T1b | N0 | Stage IA | 3.065309525 | Low-risk | 3.710526316 |
| TCGA-55-5899-01 | 930 | 0 | 58 | male | T1a | N1 | NA | 3.042595889 | Low-risk | 10.15789474 |
| TCGA-69-7978-01 | 134 | 0 | 59 | male | T2b | N1 | Stage IIB | 3.032355689 | Low-risk | 14.02631579 |
| TCGA-L4-A4E6-01 | 435 | 0 | 67 | male | T1 | N0 | Stage IA | 3.03174824 | Low-risk | 0.236842105 |
| TCGA-62-A46V-01 | 2199 | 0 | 78 | female | T2 | N0 | Stage IB | 3.007528812 | Low-risk | 1.368421053 |
| TCGA-50-5939-01 | 460 | 1 | 85 | male | T2 | N0 | Stage IB | 2.990053763 | Low-risk | 1.894736842 |
| TCGA-55-7728-01 | 704 | 0 | 64 | female | T2a | N0 | Stage IB | 2.957801432 | Low-risk | 2.263157895 |
| TCGA-50-8460-01 | 829 | 0 | 74 | male | T1a | N0 | Stage IA | 2.915525748 | Low-risk | 0.763157895 |
| TCGA-95-7948-01 | 476 | 0 | 42 | female | T2a | N0 | Stage IB | 2.908694395 | Low-risk | 3.736842105 |
| TCGA-62-A46P-01 | 594 | 1 | 65 | male | T2 | N0 | Stage IB | 2.90280458 | Low-risk | 6.605263158 |
| TCGA-97-7554-01 | 775 | 0 | 83 | female | T2a | N2 | Stage IIIA | 2.817361475 | Low-risk | 10.47368421 |
| TCGA-78-7539-01 | 791 | 0 | 75 | female | T2b | N0 | Stage IIA | 2.797805726 | Low-risk | 11.02631579 |
| TCGA-86-8054-01 | 1148 | 0 | 61 | male | T2b | N1 | Stage IIB | 2.766526608 | Low-risk | 5.210526316 |
| TCGA-86-7953-01 | 997 | 0 | 69 | female | T1b | N0 | Stage IA | 2.759904135 | Low-risk | 1.263157895 |
| TCGA-62-8398-01 | 444 | 1 | 55 | male | T2 | N2 | Stage IIIA | 2.748752017 | Low-risk | 1.894736842 |
| TCGA-86-A456-01 | 896 | 0 | 78 | female | T1a | N0 | Stage IA | 2.745861752 | Low-risk | 8.5 |
| TCGA-62-A471-01 | 1246 | 0 | 64 | male | T2b | N1 | Stage IIB | 2.729128842 | Low-risk | 2.263157895 |
| TCGA-NJ-A4YF-01 | 2161 | 0 | 50 | female | T1 | N0 | Stage IA | 2.72369949 | Low-risk | 14.18421053 |
| TCGA-93-A4JO-01 | 33 | 1 | 70 | male | T1a | N0 | Stage IA | 2.66578489 | Low-risk | 1.368421053 |
| TCGA-55-A492-01 | 596 | 0 | 70 | female | T1a | N0 | Stage IA | 2.654861993 | Low-risk | 6.684210526 |
| TCGA-44-7662-01 | 218 | 0 | 61 | male | T2a | N0 | Stage IB | 2.638582466 | Low-risk | 13.94736842 |
| TCGA-83-5908-01 | 824 | 0 | 59 | female | T1 | N0 | Stage IA | 2.633415262 | Low-risk | 5.526315789 |
| TCGA-50-5051-01 | 478 | 1 | 42 | female | T2 | N2 | Stage IIIA | 2.622191264 | Low-risk | 2.631578947 |
| TCGA-95-8039-01 | 830 | 0 | 72 | male | T1 | N0 | Stage IA | 2.620831754 | Low-risk | 2.263157895 |
| TCGA-50-6591-01 | 119 | 1 | 63 | female | T2 | N0 | Stage IV | 2.586720069 | Low-risk | 1.421052632 |
| TCGA-MP-A4TE-01 | 896 | 1 | 56 | male | T2b | N0 | Stage IIA | 2.580196431 | Low-risk | 3.236842105 |
| TCGA-05-4382-01 | 607 | 0 | 68 | male | T2 | N0 | Stage IB | 2.577040003 | Low-risk | 40.34210526 |
| TCGA-49-6744-01 | 1683 | 0 | 64 | female | T2a | N1 | Stage IIA | 2.571876534 | Low-risk | 5.578947368 |
| TCGA-MP-A4SV-01 | 2620 | 1 | 67 | male | T2 | N0 | Stage IB | 2.564777564 | Low-risk | 7.736842105 |
| TCGA-55-6642-01 | 2449 | 0 | 63 | male | T2 | N0 | Stage IB | 2.534802202 | Low-risk | 2.947368421 |
| TCGA-78-8662-01 | 3361 | 1 | 53 | female | T2 | N0 | Stage IB | 2.51244745 | Low-risk | 26.10526316 |
| TCGA-62-8397-01 | 1289 | 0 | 70 | female | T3 | N0 | Stage IIB | 2.505417822 | Low-risk | 0.631578947 |
| TCGA-97-8171-01 | 568 | 0 | 81 | male | T2a | N2 | Stage IV | 2.502136035 | Low-risk | 1.315789474 |
| TCGA-91-7771-01 | 492 | 0 | 62 | male | T3 | N0 | Stage IIB | 2.489855126 | Low-risk | 4.868421053 |
| TCGA-78-7159-01 | 1974 | 0 | 60 | female | T1 | NX | Stage IA | 2.489590284 | Low-risk | 7.157894737 |
| TCGA-95-A4VP-01 | 605 | 0 | 66 | female | T2b | N2 | Stage IIIA | 2.465695328 | Low-risk | 2.894736842 |
| TCGA-55-8089-01 | 702 | 1 | 56 | male | T1a | N0 | Stage IA | 2.451821724 | Low-risk | 27.97368421 |
| TCGA-55-7914-01 | 187 | 1 | 71 | female | T1b | N1 | Stage IIA | 2.410013122 | Low-risk | 4.236842105 |
| TCGA-97-A4M0-01 | 652 | 0 | 60 | female | T2a | N0 | Stage IB | 2.402947996 | Low-risk | 9.078947368 |
| TCGA-50-8459-01 | 1119 | 0 | 68 | male | T3 | N0 | Stage IIB | 2.359986497 | Low-risk | 2 |
| TCGA-78-8648-01 | 1209 | 1 | 58 | female | T3 | N0 | Stage IIB | 2.295281131 | Low-risk | 0.342105263 |
| TCGA-55-8096-01 | 719 | 1 | 67 | female | T2a | N0 | Stage IB | 2.26511879 | Low-risk | 3.473684211 |
| TCGA-55-7574-01 | 995 | 1 | 64 | female | T2a | N0 | Stage IB | 2.215196646 | Low-risk | 6.236842105 |
| TCGA-38-7271-01 | 800 | 1 | 72 | female | T1 | N0 | Stage IA | 2.199021756 | Low-risk | 2.289473684 |
| TCGA-L4-A4E5-01 | 578 | 0 | 48 | female | T1 | N0 | Stage I | 2.191990152 | Low-risk | 7.631578947 |
| TCGA-78-8655-01 | 2360 | 0 | 77 | female | T1 | N0 | Stage IA | 2.178723296 | Low-risk | 2.473684211 |
| TCGA-44-7660-01 | 592 | 0 | 72 | male | T2 | N0 | Stage IB | 2.144640672 | Low-risk | 8.605263158 |
| TCGA-91-6831-01 | 310 | 0 | 66 | male | T2 | N0 | Stage IB | 2.131155632 | Low-risk | 6.710526316 |
| TCGA-49-AARN-01 | 1135 | 1 | 56 | female | T1 | N0 | Stage IA | 2.12102229 | Low-risk | 18.07894737 |
| TCGA-78-7536-01 | 244 | 1 | 69 | male | T2 | N2 | Stage IIIA | 2.114680084 | Low-risk | 13.63157895 |
| TCGA-62-8394-01 | 139 | 1 | 65 | female | T4 | N2 | Stage IIIB | 2.112465405 | Low-risk | 3.026315789 |
| TCGA-44-3919-01 | 1026 | 1 | 71 | female | T1 | N0 | Stage IA | 2.108270924 | Low-risk | 1.342105263 |
| TCGA-78-7633-01 | 1528 | 1 | 67 | male | T2 | N0 | Stage IB | 2.077995788 | Low-risk | 2.552631579 |
| TCGA-55-8091-01 | 600 | 0 | 74 | male | T2 | N0 | Stage IB | 2.072180128 | Low-risk | 0.710526316 |
| TCGA-55-8204-01 | 515 | 0 | 87 | female | T2a | N0 | Stage IB | 2.06530538 | Low-risk | 5 |
| TCGA-55-7576-01 | 670 | 0 | 54 | male | T2a | N0 | Stage IB | 2.064929603 | Low-risk | 9.289473684 |
| TCGA-50-5931-01 | 434 | 1 | 75 | female | T2 | N0 | Stage IB | 2.050113687 | Low-risk | 7.236842105 |
| TCGA-55-7727-01 | 119 | 0 | 70 | male | T1a | N2 | Stage IIIA | 2.037983866 | Low-risk | 5.710526316 |
| TCGA-64-5774-01 | 2676 | 0 | 60 | male | T2 | N0 | Stage IB | 2.020151953 | Low-risk | 3.263157895 |
| TCGA-78-7143-01 | 4961 | 1 | 62 | female | T2 | N0 | Stage IB | 2.018098185 | Low-risk | NA |
| TCGA-49-AARO-01 | 3759 | 0 | 39 | female | T1a | N0 | Stage IA | 2.010878458 | Low-risk | 14.05263158 |
| TCGA-MP-A4TA-01 | 950 | 1 | 75 | female | T1 | N0 | Stage IA | 1.995913566 | Low-risk | 8.157894737 |
| TCGA-MP-A4T9-01 | 1265 | 1 | 54 | female | T2 | N2 | Stage IIIA | 1.986026609 | Low-risk | 0.763157895 |
| TCGA-62-A46R-01 | 1725 | 1 | 54 | female | T2 | N0 | Stage IB | 1.985122801 | Low-risk | 4.421052632 |
| TCGA-50-5946-02 | 1617 | 0 | 62 | male | T1 | N0 | Stage IA | 1.980568633 | Low-risk | NA |
| TCGA-67-3771-01 | 610 | 0 | 77 | female | T1 | N0 | Stage IA | 1.968830124 | Low-risk | 24.26315789 |
| TCGA-05-5715-01 | 62 | 0 | 69 | female | T2a | N0 | Stage IB | 1.964021408 | Low-risk | 2.710526316 |
| TCGA-05-4432-01 | 761 | 0 | 66 | male | T2 | N1 | Stage IIB | 1.929219028 | Low-risk | 13.57894737 |
| TCGA-55-7994-01 | 603 | 0 | 81 | male | T3 | N0 | Stage IIB | 1.908954681 | Low-risk | 36.10526316 |
| TCGA-44-8120-01 | 260 | 0 | 58 | male | T2a | N0 | Stage IB | 1.864241487 | Low-risk | 13.97368421 |
| TCGA-78-7160-01 | 697 | 1 | 61 | male | T4 | N2 | Stage IV | 1.855779692 | Low-risk | 0.973684211 |
| TCGA-50-5932-01 | 1235 | 1 | 75 | male | T2 | N1 | Stage IIB | 1.809546576 | Low-risk | 1.789473684 |
| TCGA-NJ-A7XG-01 | 617 | 0 | 49 | male | T4 | N1 | Stage IIIA | 1.773141041 | Low-risk | 0.710526316 |
| TCGA-49-AAR2-01 | 2224 | 0 | 64 | male | T2 | N0 | Stage IB | 1.77292308 | Low-risk | 9.473684211 |
| TCGA-55-6983-01 | 2823 | 0 | 81 | male | T2 | N1 | Stage IIB | 1.765614997 | Low-risk | 2.631578947 |
| TCGA-55-8510-01 | 539 | 0 | 55 | female | T2a | N0 | Stage IB | 1.738439793 | Low-risk | 7.131578947 |
| TCGA-91-6849-01 | 35 | 0 | 75 | female | T2 | N2 | Stage IIIA | 1.735698504 | Low-risk | 2.578947368 |
| TCGA-62-A46S-01 | 1653 | 1 | 73 | male | T2 | N0 | Stage IB | 1.733173106 | Low-risk | 2.868421053 |
| TCGA-55-8511-01 | 552 | 0 | 73 | female | T2a | N0 | Stage IB | 1.722820889 | Low-risk | 13.44736842 |
| TCGA-50-5930-01 | 282 | 1 | 47 | male | T2 | N2 | Stage IIIA | 1.708810824 | Low-risk | 25.5 |
| TCGA-50-7109-01 | 308 | 1 | 60 | male | T1 | N0 | Stage IA | 1.691022674 | Low-risk | 5.947368421 |
| TCGA-50-5066-02 | 1442 | 0 | 72 | male | T2 | N0 | Stage IB | 1.66866915 | Low-risk | NA |
| TCGA-L9-A444-01 | 307 | 0 | 60 | female | T1a | N0 | Stage IA | 1.629181654 | Low-risk | 12.78947368 |
| TCGA-67-3774-01 | 385 | 0 | 73 | female | T2 | N0 | Stage IB | 1.615130704 | Low-risk | 2.421052632 |
| TCGA-55-8508-01 | 617 | 0 | 60 | female | T2a | N1 | Stage IIA | 1.613968907 | Low-risk | 4.236842105 |
| TCGA-44-8119-01 | 285 | 0 | 73 | male | T3 | N0 | Stage IIB | 1.609063508 | Low-risk | 14.89473684 |
| TCGA-75-6206-01 | 2590 | 0 | NA | male | T2 | N0 | Stage IB | 1.606602772 | Low-risk | 2.131578947 |
| TCGA-55-8208-01 | 674 | 0 | 73 | female | T1b | N0 | Stage IA | 1.597663117 | Low-risk | 8.210526316 |
| TCGA-49-6742-01 | 488 | 1 | 70 | male | T2a | N1 | Stage IIA | 1.596980482 | Low-risk | 6 |
| TCGA-05-4402-01 | 244 | 1 | 57 | female | T2 | NX | Stage IV | 1.588957562 | Low-risk | 3.078947368 |
| TCGA-97-A4LX-01 | 614 | 0 | 81 | male | T2a | N0 | Stage IB | 1.566257586 | Low-risk | 3.842105263 |
| TCGA-55-7815-01 | 773 | 0 | 76 | male | T2a | N0 | Stage IB | 1.553235321 | Low-risk | 3.263157895 |
| TCGA-86-8075-01 | 694 | 1 | 66 | female | T2 | N0 | Stage IB | 1.50450798 | Low-risk | 1.263157895 |
| TCGA-62-8399-01 | 2696 | 0 | 62 | male | T2 | N2 | Stage IIIA | 1.502130807 | Low-risk | 12.10526316 |
| TCGA-05-4405-01 | 610 | 0 | 74 | female | T2 | N0 | Stage IB | 1.482087479 | Low-risk | 9.184210526 |
| TCGA-78-7156-01 | 976 | 1 | 62 | male | T4 | N1 | Stage IV | 1.475074543 | Low-risk | 7.763157895 |
| TCGA-50-8457-01 | 1125 | 0 | 63 | female | T1a | N0 | Stage IA | 1.467669244 | Low-risk | 1.394736842 |
| TCGA-38-A44F-01 | 133 | 0 | 80 | male | T2a | N0 | Stage IB | 1.4593902 | Low-risk | 0.289473684 |
| TCGA-44-6775-01 | 705 | 0 | 72 | female | T2a | N0 | Stage IB | 1.457465164 | Low-risk | 1.631578947 |
| TCGA-78-7540-01 | 1197 | 1 | 66 | female | T2 | N0 | Stage IB | 1.453409518 | Low-risk | 0.657894737 |
| TCGA-55-8507-01 | 418 | 0 | 53 | male | T1a | N0 | Stage IA | 1.443905467 | Low-risk | 22.73684211 |
| TCGA-NJ-A4YQ-01 | 1432 | 0 | 69 | female | T1b | N0 | Stage IA | 1.435897903 | Low-risk | 26.05263158 |
| TCGA-J2-A4AE-01 | 1079 | 0 | 77 | female | T1a | N0 | Stage IA | 1.35392832 | Low-risk | 1.078947368 |
| TCGA-97-A4M2-01 | 624 | 0 | 66 | male | T1a | N0 | Stage IA | 1.339205264 | Low-risk | 0.684210526 |
| TCGA-MN-A4N4-01 | 1175 | 0 | 57 | male | T1b | N0 | Stage IA | 1.336125328 | Low-risk | 35.02631579 |
| TCGA-05-4424-01 | 913 | 0 | 70 | male | T3 | N0 | Stage IIB | 1.30617891 | Low-risk | 17.10526316 |
| TCGA-55-8615-01 | 446 | 0 | 67 | male | T3 | N2 | Stage IIIA | 1.302414555 | Low-risk | 2.578947368 |
| TCGA-97-7938-01 | 18 | 1 | 76 | female | T1a | N0 | Stage IA | 1.282726514 | Low-risk | 9.447368421 |
| TCGA-MP-A4TJ-01 | 339 | 1 | 62 | female | T1 | N0 | Stage IA | 1.278262471 | Low-risk | 2.210526316 |
| TCGA-49-AAR4-01 | 879 | 1 | 51 | male | T2 | N2 | Stage IIIA | 1.260474299 | Low-risk | 13.92105263 |
| TCGA-86-8074-01 | 24 | 0 | 62 | female | T1b | N1 | Stage IIA | 1.253677727 | Low-risk | 1.657894737 |
| TCGA-44-7669-01 | 574 | 1 | 59 | male | T1b | N1 | Stage IIA | 1.247102592 | Low-risk | 10.97368421 |
| TCGA-78-7150-01 | 666 | 1 | 59 | male | T2 | N1 | Stage IIB | 1.222053959 | Low-risk | 9.368421053 |
| TCGA-62-A470-01 | 1194 | 1 | 84 | male | T2 | N0 | Stage IB | 1.218768913 | Low-risk | 2.763157895 |
| TCGA-55-6975-01 | 118 | 1 | 61 | male | T2 | N1 | Stage IIB | 1.180248549 | Low-risk | 2.026315789 |
| TCGA-MN-A4N5-01 | 84 | 0 | 63 | male | T1a | N0 | Stage IA | 1.170339072 | Low-risk | 10.5 |
| TCGA-91-A4BC-01 | 44 | 0 | 59 | male | T2b | N0 | Stage IIA | 1.168591019 | Low-risk | 10.5 |
| TCGA-MP-A4SW-01 | 1778 | 1 | 53 | male | T2 | N1 | Stage IIB | 1.133846346 | Low-risk | 1.526315789 |
| TCGA-05-4433-01 | 730 | 0 | 82 | male | T2 | N0 | Stage IB | 1.100853337 | Low-risk | 0.842105263 |
| TCGA-64-1681-01 | 1167 | 1 | 61 | female | T1 | N0 | Stage IA | 1.085063358 | Low-risk | 2 |
| TCGA-55-6985-01 | 1233 | 0 | 58 | female | T2 | N0 | Stage IB | 1.056136101 | Low-risk | 10.71052632 |
| TCGA-91-6830-01 | 60 | 0 | 65 | female | T1 | N1 | Stage IIA | 1.019867865 | Low-risk | 5.605263158 |
| TCGA-44-6779-01 | 500 | 1 | 50 | female | T2 | N1 | Stage IIB | 1.001376081 | Low-risk | 2.315789474 |
| TCGA-97-8179-01 | 435 | 0 | 72 | male | T1a | N0 | Stage IA | 0.955452007 | Low-risk | 3.921052632 |
| TCGA-50-5944-01 | 1750 | 0 | 69 | female | T1 | N0 | Stage IA | 0.943664804 | Low-risk | 1.368421053 |
| TCGA-L9-A8F4-01 | 476 | 0 | 64 | female | T2a | N0 | Stage IB | 0.923115368 | Low-risk | 17.94736842 |
| TCGA-62-8402-01 | 1498 | 1 | 73 | female | T2 | N2 | Stage IIIA | 0.898598123 | Low-risk | 1.026315789 |
| TCGA-78-7162-01 | 3169 | 1 | 75 | male | T1 | N0 | Stage IA | 0.882518153 | Low-risk | 1.894736842 |
| TCGA-44-7659-01 | 691 | 0 | 70 | male | T1b | N0 | Stage IA | 0.834362603 | Low-risk | 3.552631579 |
| TCGA-05-4397-01 | 731 | 1 | 65 | male | T2 | N1 | Stage IIB | 0.824770341 | Low-risk | 19.28947368 |
| TCGA-55-6981-01 | 1379 | 1 | 53 | female | T1 | N2 | Stage IIIA | 0.765380595 | Low-risk | 1.315789474 |
| TCGA-L9-A7SV-01 | 565 | 0 | 69 | male | T2a | N1 | Stage IIA | 0.720285385 | Low-risk | 36.15789474 |
| TCGA-55-8505-01 | 440 | 0 | 62 | male | T1a | N2 | Stage IIIA | 0.714278102 | Low-risk | 0.736842105 |
| TCGA-55-8616-01 | 48 | 0 | 58 | female | T2a | N0 | Stage IB | 0.706613646 | Low-risk | 11.21052632 |
| TCGA-49-AARQ-01 | 6732 | 0 | 41 | female | T2 | N0 | Stage I | 0.703359316 | Low-risk | 16.71052632 |
| TCGA-J2-8192-01 | 739 | 0 | 65 | female | T2a | N1 | Stage IIA | 0.698518275 | Low-risk | 1.789473684 |
| TCGA-55-7724-01 | 705 | 0 | 76 | female | T2a | N0 | Stage IB | 0.672462754 | Low-risk | 5.473684211 |
| TCGA-69-7973-01 | 230 | 0 | 42 | female | T2a | N0 | Stage IB | 0.621881854 | Low-risk | 6.157894737 |
| TCGA-44-5643-01 | 1013 | 0 | 53 | male | T2b | N2 | Stage IIIA | 0.619611773 | Low-risk | 2.868421053 |
| TCGA-86-8279-01 | 949 | 0 | 46 | male | T2a | N1 | Stage IIA | 0.619211313 | Low-risk | 11.78947368 |
| TCGA-05-4422-01 | 365 | 0 | 68 | male | T2 | N0 | Stage IB | 0.59066195 | Low-risk | 1.026315789 |
| TCGA-55-8514-01 | 520 | 0 | 70 | female | T2a | N0 | Stage IB | 0.570082402 | Low-risk | 6.447368421 |
| TCGA-J2-A4AG-01 | 988 | 0 | 66 | female | T1b | N0 | Stage IA | 0.538516799 | Low-risk | 2.157894737 |
| TCGA-86-8073-01 | 740 | 0 | 58 | male | T2a | N0 | Stage IB | 0.5261338 | Low-risk | 34.68421053 |
| TCGA-67-6217-01 | 422 | 0 | 73 | female | T2a | N1 | Stage IIA | 0.507016275 | Low-risk | 4.552631579 |
| TCGA-55-8621-01 | 515 | 0 | 75 | female | T1a | N0 | Stage IA | 0.502933904 | Low-risk | 1.789473684 |
| TCGA-44-7670-01 | 882 | 0 | 47 | female | T1b | N1 | Stage IIA | 0.480336373 | Low-risk | 25.47368421 |
| TCGA-97-7553-01 | 1870 | 0 | 58 | female | T1 | N0 | Stage IA | 0.45749656 | Low-risk | 1.105263158 |
| TCGA-53-7624-01 | 1043 | 1 | 40 | female | T2 | N0 | Stage IV | 0.452342662 | Low-risk | 19 |
| TCGA-71-6725-01 | 256 | 0 | 48 | female | T2 | N0 | Stage IB | 0.408596552 | Low-risk | 1.210526316 |
| TCGA-44-A4SS-01 | 415 | 0 | 73 | male | T1b | N0 | Stage IA | 0.388691081 | Low-risk | 11.60526316 |
| TCGA-91-6835-01 | 79 | 0 | 81 | female | T1 | N0 | Stage IA | 0.370185691 | Low-risk | 2.052631579 |
| TCGA-97-7941-01 | 484 | 0 | 72 | female | T1b | N0 | Stage IA | 0.362188324 | Low-risk | 2.210526316 |
| TCGA-44-6145-01 | 595 | 0 | 62 | female | T1 | N0 | Stage IA | 0.347446278 | Low-risk | 7.552631579 |
| TCGA-38-4630-01 | 1073 | 1 | 75 | female | T2 | N0 | Stage IB | 0.342259509 | Low-risk | 2.921052632 |
| TCGA-S2-AA1A-01 | 513 | 0 | 68 | female | T1b | N0 | Stage IA | 0.341308955 | Low-risk | 2.710526316 |
| TCGA-86-7701-01 | 947 | 0 | 66 | male | T2 | N0 | Stage IV | 0.247246377 | Low-risk | 4.684210526 |
| TCGA-38-6178-01 | 448 | 0 | 70 | female | T2b | N2 | Stage IIIA | 0.214711236 | Low-risk | 1.684210526 |
| TCGA-67-4679-01 | 448 | 0 | 69 | male | T3 | N0 | NA | 0.211780417 | Low-risk | 6.657894737 |
| TCGA-91-6829-01 | 1258 | 1 | 78 | male | T2 | N0 | Stage IB | 0.171729056 | Low-risk | 15.02631579 |
| TCGA-44-2657-01 | 1351 | 0 | 74 | female | T2 | NX | Stage IB | 0.170183542 | Low-risk | 9.447368421 |
| TCGA-55-8619-01 | 416 | 0 | 72 | female | T3 | N0 | Stage IIB | 0.166731212 | Low-risk | 0.526315789 |
| TCGA-73-A9RS-01 | 340 | 1 | 41 | male | T3 | N0 | Stage IIB | 0.136507276 | Low-risk | 21.94736842 |
| TCGA-99-AA5R-01 | 658 | 0 | 70 | female | T1a | N0 | Stage IA | 0.132885693 | Low-risk | 1.421052632 |
| TCGA-91-6828-01 | 323 | 0 | 70 | male | T1a | N0 | Stage IA | 0.089958078 | Low-risk | 6.894736842 |
| TCGA-55-1594-01 | 1178 | 1 | 68 | male | T2 | N2 | Stage IIIA | 0.071550818 | Low-risk | 4.236842105 |
| TCGA-55-A494-01 | 481 | 0 | 61 | female | T2a | N0 | Stage IB | 0.049051494 | Low-risk | 5.421052632 |
| TCGA-MP-A4TH-01 | 741 | 0 | 70 | female | T1a | N0 | Stage IA | 0.004128034 | Low-risk | 2.789473684 |
| TCGA-05-4398-01 | 1431 | 1 | 47 | female | T4 | N3 | Stage IIIB | 0 | Low-risk | 17.07894737 |
| TCGA-05-5420-01 | 457 | 0 | 67 | male | T2 | N2 | Stage IIIA | 0 | Low-risk | 2.131578947 |
| TCGA-35-4122-01 | 225 | 1 | 69 | male | T1 | N0 | Stage IA | 0 | Low-risk | 7.578947368 |
| TCGA-44-2656-01 | 1429 | 0 | 59 | male | T2 | N0 | Stage IB | 0 | Low-risk | 19.57894737 |
| TCGA-44-3918-01 | 1036 | 0 | 60 | female | T1 | N0 | Stage IA | 0 | Low-risk | 22.81578947 |
| TCGA-44-7672-01 | 719 | 0 | 52 | female | T1b | N0 | Stage IA | 0 | Low-risk | 3.868421053 |
| TCGA-49-4514-01 | 1700 | 0 | 79 | female | T1 | N0 | Stage IA | 0 | Low-risk | 7.421052632 |
| TCGA-55-6971-01 | 1400 | 0 | 59 | female | T2 | N0 | Stage IB | 0 | Low-risk | 2.078947368 |
| TCGA-55-6980-01 | 2109 | 0 | 56 | male | T1 | N0 | Stage IA | 0 | Low-risk | 0.394736842 |
| TCGA-55-6987-01 | 2137 | 0 | 77 | male | T1 | N0 | Stage IA | 0 | Low-risk | 5 |
| TCGA-55-8203-01 | 547 | 0 | 69 | female | T1b | N0 | Stage IA | 0 | Low-risk | 9.947368421 |
| TCGA-55-8512-01 | 607 | 1 | 41 | male | T1a | N1 | Stage IV | 0 | Low-risk | 0.868421053 |
| TCGA-64-5781-01 | 1559 | 0 | 55 | female | T2 | N0 | Stage IB | 0 | Low-risk | 26.39473684 |
| TCGA-64-5815-01 | 866 | 0 | 74 | male | T2 | N1 | Stage IIB | 0 | Low-risk | 3.789473684 |
| TCGA-69-8255-01 | 129 | 1 | 71 | male | T1a | N0 | Stage IA | 0 | Low-risk | 6.973684211 |
| TCGA-78-7161-01 | 291 | 1 | 69 | female | T3 | N0 | Stage IIB | 0 | Low-risk | 4.210526316 |
| TCGA-78-8640-01 | 7062 | 0 | 59 | male | T1 | N1 | Stage IIA | 0 | Low-risk | 16.71052632 |
| TCGA-80-5608-01 | 2832 | 0 | NA | female | T1 | N0 | Stage IA | 0 | Low-risk | 3.894736842 |
| TCGA-80-5611-01 | 2595 | 0 | NA | male | T2 | N0 | Stage IB | 0 | Low-risk | 4.921052632 |
| TCGA-93-8067-01 | 186 | 0 | 77 | male | T2a | N0 | Stage IB | 0 | Low-risk | 11.73684211 |
| TCGA-95-7043-01 | 503 | 1 | 63 | female | T1a | N0 | Stage IA | 0 | Low-risk | 26.05263158 |
| TCGA-95-7944-01 | 377 | 1 | 71 | male | T1a | N0 | Stage IA | 0 | Low-risk | 5.315789474 |
| TCGA-97-8174-01 | 164 | 1 | 67 | male | T2b | N0 | Stage IIA | 0 | Low-risk | 4.078947368 |
| TCGA-99-7458-01 | 747 | 0 | 74 | female | T4 | N0 | Stage IIIA | 0 | Low-risk | 11.26315789 |
| TCGA-NJ-A4YI-01 | 4 | 1 | 87 | female | T2 | N2 | Stage IIIA | 0 | Low-risk | 10.28947368 |
| TCGA-86-8280-01 | 701 | 0 | 54 | female | T2b | N0 | Stage IIA | -0.010064279 | Low-risk | 1.131578947 |
| TCGA-55-1596-01 | 2065 | 0 | 55 | male | T2 | N1 | Stage IIB | -0.020298413 | Low-risk | 4.5 |
| TCGA-73-4662-01 | 2515 | 0 | 65 | female | T1 | N0 | Stage IA | -0.033012833 | Low-risk | 5.184210526 |
| TCGA-86-8278-01 | 944 | 0 | 63 | female | T2 | N1 | Stage IIB | -0.044841096 | Low-risk | 2.421052632 |
| TCGA-44-6778-01 | 1864 | 0 | 59 | male | T1 | N0 | Stage IA | -0.067886977 | Low-risk | 8.947368421 |
| TCGA-49-AAR9-01 | 260 | 1 | 61 | male | T3 | N0 | Stage IIB | -0.127550815 | Low-risk | 14.65789474 |
| TCGA-86-7711-01 | 1046 | 1 | 70 | male | T2a | N1 | Stage IIA | -0.131297918 | Low-risk | 5 |
| TCGA-44-A479-01 | 486 | 0 | 73 | female | T2 | N0 | Stage IB | -0.217620338 | Low-risk | 7.289473684 |
| TCGA-86-6562-01 | 376 | 1 | 52 | male | T2a | N1 | Stage IIA | -0.254370081 | Low-risk | 1.315789474 |
| TCGA-50-5946-01 | 1617 | 0 | 62 | male | T1 | N0 | Stage IA | -0.262176532 | Low-risk | 15.10526316 |
| TCGA-05-4249-01 | 1523 | 0 | 67 | male | T2 | N0 | Stage IB | -0.266029262 | Low-risk | 7.605263158 |
| TCGA-64-5779-01 | 864 | 0 | 61 | male | T2 | N2 | Stage IIIA | -0.323149822 | Low-risk | 8.394736842 |
| TCGA-44-6147-01 | 845 | 0 | 67 | female | T1b | NX | Stage IA | -0.345362941 | Low-risk | 2.578947368 |
| TCGA-97-A4M1-01 | 601 | 0 | 52 | female | T1a | N0 | Stage IA | -0.348855776 | Low-risk | 1.026315789 |
| TCGA-86-7955-01 | 1072 | 0 | 62 | male | T2a | N0 | Stage IB | -0.399374372 | Low-risk | 7.236842105 |
| TCGA-44-6776-01 | 2616 | 0 | 60 | female | T1 | N0 | Stage IA | -0.475418543 | Low-risk | 4.736842105 |
| TCGA-95-A4VK-01 | 651 | 0 | 74 | female | T2b | N2 | Stage IIIA | -0.581444013 | Low-risk | 5.815789474 |
| TCGA-55-7570-01 | 824 | 0 | 60 | male | T1a | N0 | Stage IA | -0.600296194 | Low-risk | 8.921052632 |
| TCGA-55-7816-01 | 468 | 1 | 49 | female | TX | NX | Stage IV | -0.631920027 | Low-risk | 0.052631579 |
| TCGA-86-6851-01 | 179 | 0 | 73 | female | T1b | N1 | Stage IIA | -0.636194327 | Low-risk | 21.10526316 |
| TCGA-97-8552-01 | 626 | 0 | 55 | female | T1a | N0 | Stage I | -0.678155877 | Low-risk | 0.605263158 |
| TCGA-53-7626-01 | 929 | 1 | 76 | female | T1 | N1 | Stage IIA | -0.707823266 | Low-risk | 8.578947368 |
| TCGA-86-7713-01 | 1157 | 0 | 70 | male | T2b | N0 | Stage IIA | -0.708026042 | Low-risk | 3.657894737 |
| TCGA-55-8092-01 | 154 | 1 | 75 | male | T3 | N0 | Stage IIB | -0.710778279 | Low-risk | 12.65789474 |
| TCGA-86-A4P7-01 | 415 | 0 | 63 | female | T2a | N0 | Stage IB | -0.727271632 | Low-risk | 1.710526316 |
| TCGA-69-A59K-01 | 591 | 0 | 60 | female | T3 | N0 | Stage IIB | -0.752393714 | Low-risk | 11.36842105 |
| TCGA-86-7714-01 | 625 | 1 | 61 | female | T1b | N2 | Stage IIIA | -0.774128357 | Low-risk | 1.789473684 |
| TCGA-L9-A50W-01 | 442 | 1 | 75 | male | T1b | N1 | Stage IIA | -0.795557998 | Low-risk | 0.815789474 |
| TCGA-44-A47B-01 | 287 | 0 | 79 | male | T2a | N0 | Stage IB | -0.84557838 | Low-risk | 1.026315789 |
| TCGA-69-7980-01 | 411 | 0 | 70 | female | T1b | N0 | Stage I | -0.859808077 | Low-risk | 19 |
| TCGA-J2-A4AD-01 | 550 | 1 | 61 | female | T1a | N0 | Stage IA | -0.877621722 | Low-risk | 7.710526316 |
| TCGA-05-4427-01 | 791 | 0 | 65 | female | T2 | N1 | Stage IIB | -0.917431861 | Low-risk | 24 |
| TCGA-69-8254-01 | 409 | 0 | 85 | male | T2b | NA | NA | -1.00288041 | Low-risk | 1.947368421 |
| TCGA-69-7761-01 | 186 | 0 | 84 | male | T2a | N0 | Stage IB | -1.006882007 | Low-risk | 0.763157895 |
| TCGA-55-8614-01 | 536 | 0 | 76 | male | T2a | N0 | Stage IB | -1.012041959 | Low-risk | 5.315789474 |
| TCGA-55-A4DF-01 | 440 | 1 | 88 | male | T1b | N0 | Stage IA | -1.019123769 | Low-risk | 17.47368421 |
| TCGA-55-6968-01 | 1293 | 1 | 61 | male | T1 | N0 | Stage IV | -1.019276971 | Low-risk | 11.76315789 |
| TCGA-97-A4M7-01 | 629 | 0 | 74 | male | T1b | N0 | Stage IA | -1.092256812 | Low-risk | 7.157894737 |
| TCGA-49-6743-01 | 1621 | 0 | 81 | female | T1 | N2 | Stage IIIA | -1.092702452 | Low-risk | 17.39473684 |
| TCGA-50-5935-01 | 653 | 1 | 86 | female | T1 | N0 | Stage IA | -1.108850039 | Low-risk | 2.368421053 |
| TCGA-53-A4EZ-01 | 1071 | 0 | 63 | male | T2a | N1 | Stage IIA | -1.111520447 | Low-risk | 11.63157895 |
| TCGA-86-8056-01 | 139 | 0 | 63 | female | T4 | N0 | Stage IIIA | -1.1255606 | Low-risk | 6.736842105 |
| TCGA-55-8206-01 | 888 | 0 | 56 | male | T1b | N0 | Stage IA | -1.131556345 | Low-risk | 0.710526316 |
| TCGA-55-7284-01 | 243 | 1 | 74 | male | T3 | N0 | Stage IIB | -1.195667641 | Low-risk | 1.026315789 |
| TCGA-55-8506-01 | 11 | 0 | 62 | female | T3 | N0 | Stage IIB | -1.198575105 | Low-risk | 41.10526316 |
| TCGA-86-8671-01 | 839 | 0 | 72 | female | T2b | N1 | Stage IIB | -1.204592238 | Low-risk | 0.868421053 |
| TCGA-69-7764-01 | 414 | 0 | 75 | male | T1b | N0 | Stage IA | -1.231434306 | Low-risk | 2.026315789 |
| TCGA-50-5942-01 | 1847 | 0 | 67 | female | T1 | N0 | Stage IA | -1.246267286 | Low-risk | 1.5 |
| TCGA-86-8668-01 | 423 | 0 | 61 | female | T1b | N0 | Stage IA | -1.250545561 | Low-risk | 0.868421053 |
| TCGA-75-7025-01 | 3305 | 0 | NA | male | T2 | N0 | Stage IB | -1.376978868 | Low-risk | 0.710526316 |
| TCGA-86-A4P8-01 | 805 | 0 | 59 | female | T1b | N2 | Stage IIIA | -1.410152724 | Low-risk | 0 |
| TCGA-55-8513-01 | 791 | 0 | 77 | female | T3 | N0 | Stage IIB | -1.437669545 | Low-risk | 0.157894737 |
| TCGA-05-4384-01 | 426 | 0 | 66 | male | T2 | N2 | Stage IIIA | -1.468582002 | Low-risk | 2.973684211 |
| TCGA-44-5644-01 | 863 | 0 | 51 | female | T2a | N0 | Stage IB | -1.477168681 | Low-risk | 22.76315789 |
| TCGA-78-7220-01 | 807 | 1 | 53 | female | T2 | N2 | Stage IIIA | -1.528705425 | Low-risk | 19.02631579 |
| TCGA-NJ-A55R-01 | 603 | 0 | 67 | male | T1b | N0 | Stage IA | -1.562915439 | Low-risk | 6.026315789 |
| TCGA-75-5146-01 | 2368 | 0 | NA | male | T2 | N0 | Stage IB | -1.570074898 | Low-risk | 4.447368421 |
| TCGA-55-1592-01 | 701 | 1 | 65 | male | T2 | N0 | Stage IA | -1.584976617 | Low-risk | 12.63157895 |
| TCGA-73-7498-01 | 1189 | 0 | 58 | female | T1b | N0 | Stage IA | -1.643981526 | Low-risk | 2.5 |
| TCGA-62-8395-01 | 1216 | 0 | 80 | female | T3 | N0 | Stage IIB | -1.678893806 | Low-risk | 1.842105263 |
| TCGA-97-7552-01 | 1932 | 0 | 70 | male | T2 | N0 | Stage IB | -1.753491659 | Low-risk | 0.763157895 |
| TCGA-55-A48X-01 | 689 | 0 | 63 | female | T1b | N1 | Stage IIA | -1.808875241 | Low-risk | 9.421052632 |
| TCGA-69-7979-01 | 408 | 0 | 71 | female | T2a | N0 | Stage IB | -1.81722904 | Low-risk | 34.18421053 |
| TCGA-44-A4SU-01 | 409 | 1 | 67 | female | T1a | N0 | Stage IA | -1.835380873 | Low-risk | 3.447368421 |
| TCGA-71-8520-01 | 210 | 1 | 60 | female | T2 | N0 | Stage IB | -1.880788415 | Low-risk | 2.078947368 |
| TCGA-55-A4DG-01 | 608 | 0 | 71 | male | T1b | N0 | Stage IA | -1.901055577 | Low-risk | 16.36842105 |
| TCGA-93-7348-01 | 531 | 0 | 75 | female | T1a | N0 | Stage IA | -1.910572316 | Low-risk | 2.605263158 |
| TCGA-86-8358-01 | 653 | 0 | 44 | male | T2a | N0 | Stage IB | -1.973558164 | Low-risk | 24.78947368 |
| TCGA-91-8497-01 | 434 | 1 | 75 | female | T1a | N0 | Stage IA | -1.981628863 | Low-risk | 1 |
| TCGA-97-7547-01 | 1965 | 0 | 67 | female | T2 | N0 | Stage IB | -1.996267438 | Low-risk | 4 |
| TCGA-93-A4JP-01 | 578 | 0 | 64 | male | TX | NX | Stage IV | -2.050040476 | Low-risk | 1.421052632 |
| TCGA-86-7954-01 | 605 | 0 | 68 | female | T2 | N0 | Stage IB | -2.088831292 | Low-risk | 4.868421053 |
| TCGA-O1-A52J-01 | 1798 | 1 | 74 | female | T1 | N0 | Stage IA | -2.121668209 | Low-risk | 3.947368421 |
| TCGA-55-A57B-01 | 546 | 0 | 80 | female | T1b | N0 | Stage IA | -2.199710765 | Low-risk | 1.026315789 |
| TCGA-55-8620-01 | 375 | 1 | 60 | male | T1a | N1 | Stage IV | -2.234565976 | Low-risk | 11.28947368 |
| TCGA-97-8172-01 | 545 | 0 | 75 | female | T2a | N0 | Stage IB | -2.403134605 | Low-risk | 6.473684211 |
| TCGA-97-7546-01 | 1285 | 0 | 76 | female | T1 | N0 | Stage IA | -2.577726298 | Low-risk | 6.5 |
| TCGA-MP-A5C7-01 | 2248 | 0 | 76 | female | T2 | N0 | Stage IB | -2.63534988 | Low-risk | 1.5 |
| TCGA-78-7163-01 | 7248 | 0 | 60 | male | T2 | N0 | Stage IB | -2.756033512 | Low-risk | 0.578947368 |
| TCGA-55-8097-01 | 476 | 0 | 60 | female | T1a | N0 | Stage IA | -2.799593955 | Low-risk | 2.578947368 |
| TCGA-97-A4M6-01 | 568 | 0 | 45 | female | T1a | N0 | Stage IA | -2.842948191 | Low-risk | 0.763157895 |
| TCGA-78-7155-01 | 1171 | 1 | 68 | male | T2 | N0 | Stage IB | -2.928620043 | Low-risk | 36.15789474 |
| TCGA-95-7947-01 | 477 | 0 | 67 | male | T1a | N0 | Stage IA | -3.131001955 | Low-risk | 12.28947368 |
| TCGA-44-6146-01 | 728 | 0 | 64 | male | T3 | N0 | Stage IIB | -3.209457493 | Low-risk | 0.947368421 |
| TCGA-91-6840-01 | 372 | 0 | 59 | female | T1b | N0 | Stage IA | -3.420210885 | Low-risk | 2.657894737 |
| TCGA-93-A4JN-01 | 718 | 0 | 71 | male | T2a | N0 | Stage IV | -3.449663832 | Low-risk | 1.868421053 |
| TCGA-49-AARR-01 | 4992 | 0 | 68 | male | T1 | N0 | Stage IA | -3.478609978 | Low-risk | 0.052631579 |
| TCGA-44-5645-01 | 852 | 0 | 61 | female | T1 | NX | Stage IA | -3.530111588 | Low-risk | 0.947368421 |
| TCGA-55-7995-01 | 889 | 0 | 73 | female | T1b | N0 | Stage IA | -3.572377785 | Low-risk | 15.52631579 |
| TCGA-NJ-A55A-01 | 15 | 0 | 76 | female | T2 | N0 | Stage IB | -3.652786812 | Low-risk | 2.552631579 |
| TCGA-55-7573-01 | 487 | 0 | 72 | female | T1b | N0 | Stage IA | -3.998044641 | Low-risk | 1.263157895 |
| TCGA-44-6148-01 | 704 | 0 | 60 | male | T1b | N0 | Stage IA | -4.440900521 | Low-risk | 0.157894737 |
| TCGA-55-8087-01 | 462 | 0 | 59 | female | T2a | N0 | Stage IB | -4.584030825 | Low-risk | 0.789473684 |

NDEGS, neutrophil differentially expressed genes score; TMB, tumor mutational burden.
